# Supplementary material for: Exploiting the Chiral Ligands of Bis(imidazolinyl)- and Bis(oxazolinyl)thiophenes—Synthesis and Application in Cu-Catalyzed Friedel–Crafts Asymmetric Alkylation
Source: Molecules. 2021 Dec 6;26(23):7408. doi: 10.3390/molecules26237408 (PMC8658940; doi:10.3390/molecules26237408)
Supplement: Supplementary file 1 [file molecules-26-07408-s001.zip › molecules-1468716-supplementary.pdf]

Electronic Supplementary file (ESI)

Exploiting the Chiral Ligands of Bis(imidazolinyl)- and Bis(oxazolinyl)thiophenes - Synthesis and Application in Cu-Catalyzed Friedel-Crafts Asymmetric Alkylation

Mohammad Shahidul Islam [1,*] Abdullah Saleh Alammari [1], Assem Barakat [1], Saeed Alshahrani [1] Matti Haukka[2], and Abdullah Mohammed Al-Majid [1,*]

[1] Department of Chemistry, College of Science, King Saud University, P.O. Box 2455, Riyadh 11451, Saudi Arabia; mislam@ksu.edu.sa (M.S.I.); alamary1401@yahoo.com or 436106737@student.ksu.edu.sa (A.S.A.); ambarakat@ksu.edu.sa (A.B.); chemistry99y@gmail.com or 436106738@student.ksu.edu.sa (S.A.); amajid@ksu.edu.sa (A.M.A.-M.)

[2] Department of Chemistry, University of Jyväskylä, P.O. Box 35, FI-40014 Jyväskylä, Finland. matti.o.haukka@jyu.fi (M.H.)

* Correspondence: mislam@ksu.edu.sa (M.S.I.); amajid@ksu.edu.sa (A.M.A-M.)

Contents

1. Generals: **2**

2. NMR Spectra **3**

^{1}H NMR and ^{13}C-NMR for thiophene-2,5-dicarboxamide alcohol-4a 3
^{1}H NMR and ^{13}C-NMR for thiophene-2,5-dicarboxamide alcohol-4b 4
^{1}H NMR and ^{13}C-NMR for thiophene-2,5-dicarboxamide alcohol-4c 5
^{1}H NMR and ^{13}C-NMR for thiophene-2,5-bisimidazoline ligand-L1 6
^{1}H NMR and ^{13}C-NMR for thiophene-2,5-bisimidazoline ligand-L2 7
^{1}H NMR and ^{13}C-NMR for thiophene-2,5-bisimidazoline ligand-L3 8
^{1}H NMR and ^{13}C-NMR for thiophene-2,5-bisoxazoline ligand-L4 9
^{1}H NMR and ^{13}C-NMR for thiophene-2,5-bisoxazoline ligand-L5 10
^{1}H NMR and ^{13}C-NMR for Friedel Craft product-10a 11
^{1}H NMR and ^{13}C-NMR for Friedel Craft product-10b 12
^{1}H NMR and ^{13}C-NMR for Friedel Craft product-10c 13
^{1}H NMR and ^{13}C-NMR for Friedel Craft product-10d 14
^{1}H NMR and ^{13}C-NMR for Friedel Craft product-10e 15
^{1}H NMR and ^{13}C-NMR for Friedel Craft product-10f 16
^{1}H NMR and ^{13}C-NMR for Friedel Craft product-10g 17
^{1}H NMR and ^{13}C-NMR for Friedel Craft product-10h 18
^{1}H NMR and ^{13}C-NMR for Friedel Craft product-10i 19
^{1}H NMR and ^{13}C-NMR for Friedel Craft product-10j 20
^{1}H NMR and ^{13}C-NMR for Friedel Craft product-10k 21
^{1}H NMR and ^{13}C-NMR for Friedel Craft product-10l 22
^{1}H NMR and ^{13}C-NMR for Friedel Craft product-10m 23
^{1}H NMR and ^{13}C-NMR for Friedel Craft product-10n 24
^{1}H NMR and ^{13}C-NMR for Friedel Craft product-10o 25
^{1}H NMR and ^{13}C-NMR for Friedel Craft product-10p 26
^{1}H NMR and ^{13}C-NMR for Friedel Craft product-10q 27
^{1}H NMR and ^{13}C-NMR for Friedel Craft product-10r 28
^{1}H NMR and ^{13}C-NMR for Friedel Craft product-10s 29
^{1}H NMR and ^{13}C-NMR for Friedel Craft product-10t 30

3. Chiral HPLC Analysis **31**

Chiral HPLC purity of ligand L1 31
Chiral HPLC purity of ligand L2 31
Chiral HPLC purity of ligand L3 32
Chiral HPLC purity of ligand L4 32

Chiral HPLC purity of ligand L5 33
Chiral HPLC for Friedel Craft product-10a 33
Chiral HPLC for Friedel Craft product-10b 35
Chiral HPLC for Friedel Craft product-10c 36
Chiral HPLC for Friedel Craft product-10d 37
Chiral HPLC for Friedel Craft product-10e 38
Chiral HPLC for Friedel Craft product-10f 39
Chiral HPLC for Friedel Craft product-10g 40
Chiral HPLC for Friedel Craft product-10h 41
Chiral HPLC for Friedel Craft product-10i 42
Chiral HPLC for Friedel Craft product-10i 43
Chiral HPLC for Friedel Craft product-10k 44
Chiral HPLC for Friedel Craft product-10l 45
Chiral HPLC for Friedel Craft product-10m 46
Chiral HPLC for Friedel Craft product-10n 47
Chiral HPLC for Friedel Craft product-10o 48
Chiral HPLC for Friedel Craft product-10p 49
Chiral HPLC for Friedel Craft product-10q 50
Chiral HPLC for Friedel Craft product-10r 51
Chiral HPLC for Friedel Craft product-10s 52
Chiral HPLC for Friedel Craft product-10t 53

4. X-Ray structure determinations for SI 54

1. Generals:

Reagents obtained from commercial suppliers were used without further purification. Preparation of bis(imidazoline) and bis(oxazoline) ligands was performed in flask dried glassware under a static pressure of nitrogen. Solvents were dried prior to use following standard procedures. Reactions were monitored by thin layer chromatography using Merck silica gel 60 Kieselgel F254 TLC (Merck, Kenilworth, NJ, USA) and column chromatography was performed on silica gel 100–200 (40–63 μm, ASTM) from Merck using the indicated solvents. 1H and 13C-NMR spectra were recorded in CDCl3 and DMSO-d6 on a Jeol Spectrometer (Jeol, Tokyo, Japan) (400 MHz and 500 MHz). The chemical shifts are reported in ppm relative to CDCl3 (δ = 7.26) or d6-DMSO (δ = 2.50) for 1H-NMR. For the 13C-NMR spectra, the residual CDCl3 (δ = 77.16) or d6-DMSO (δ = 39.5). All the racemic products were freshly prepared as per the method reported in the literature [1]. Infrared spectra were recorded on a Thermo Scientific Nicolet iS10 FT-IR spectrometer (Thermo Fisher Scientific, Waltham, MA, USA). Enantiomeric ratios were determined by analytical chiral HPLC analysis on a Shimadzu LC-20A (Shimadzu, Kuoto, Japan) Prominence instrument with a chiral stationary phase using Daicel OD-H columns (Chiral Technologies Europe, Illkirch Graffenstaden, France) and 70-75% n-hexane/iso-propanol as eluents (Supplementary Materials). Optical rotations were obtained with a Perkin-Elmer 343 Polarimeter (Perkin-Elmer, Waltham, MA, USA). Melting points (m.p.) were recorded on a Thomas-Hoover capillary melting point apparatus (Thomas–Hoover, Texas City, USA) and were not corrected. Mass spectrometric analysis was done using ESI mode on AGILENT Technologies 6410-triple quad LC/MS instrument (Agilent, Santa Clara, CA, USA). Elemental analysis were performed on 'Perkin-Elmer PE 2400 CHN Elemental Analyzer with autosampler, CHN mode. X-ray diffraction data were collected on a Rigaku Oxford Diffraction Supernova diffrac-tometer and processed with CrysAlisPro software v. 1.171.41.93a (Rigaku Oxford Diffrac-tion, Yarnton, UK, 2020) using Cu K_ radiation".

2. NMR Spectra

^{1}H NMR and ^{13}C-NMR for thiophene-2,5-dicarboxamide alcohol-4a

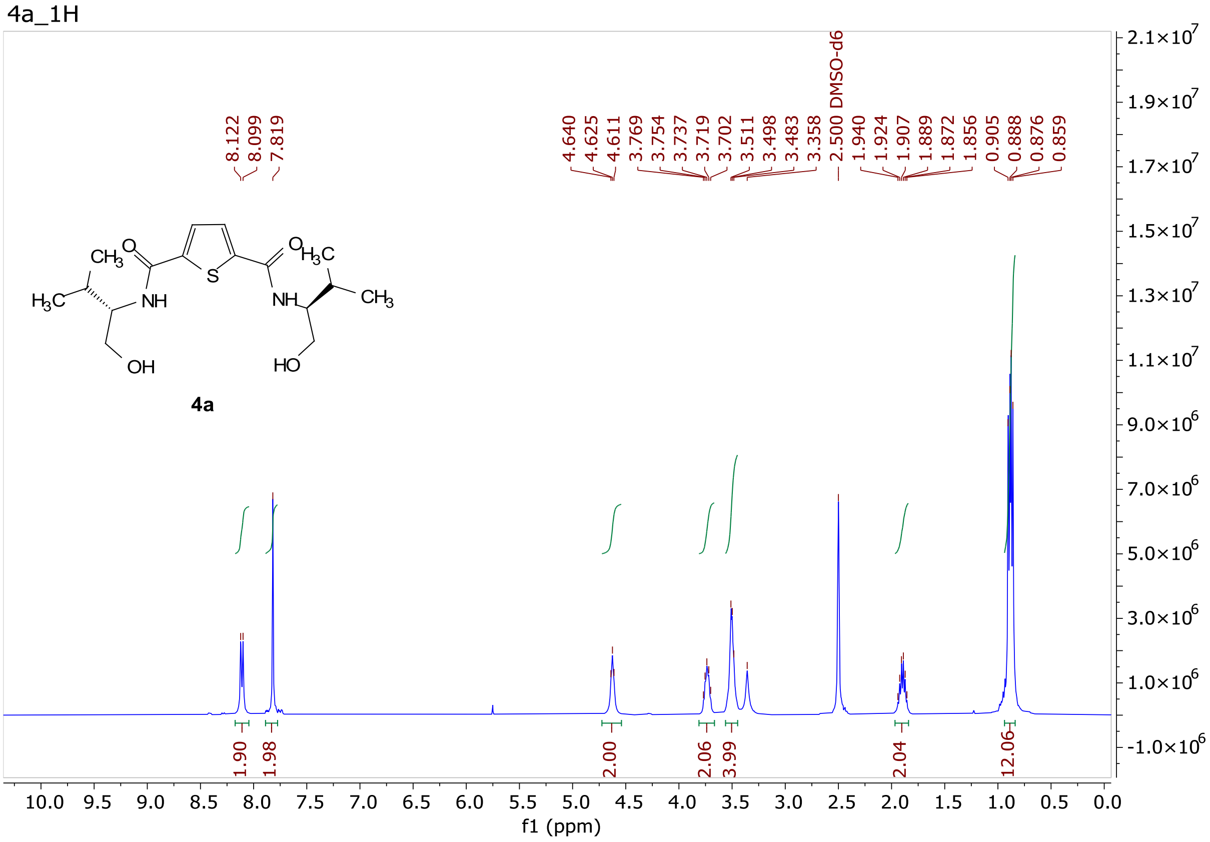

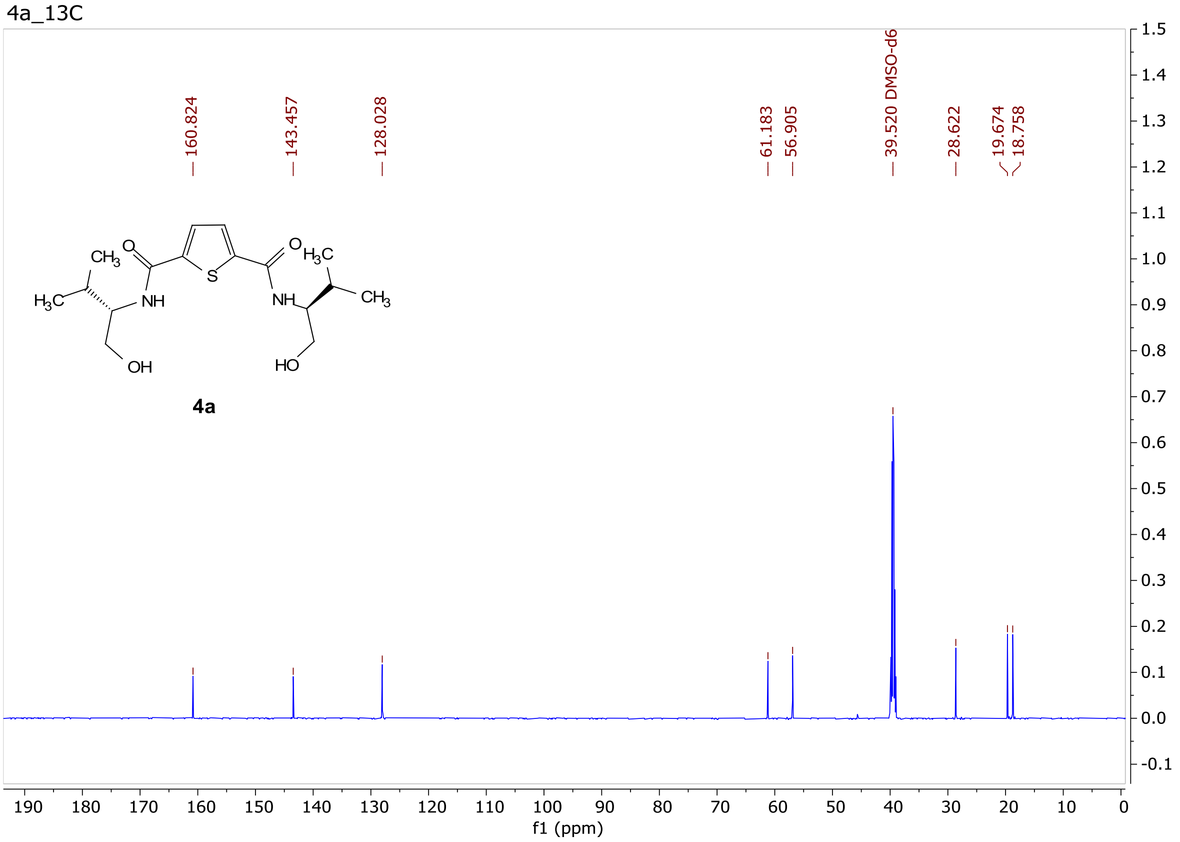

Electronic Supplementary file (ESI)

^{1}H NMR and ^{13}C-NMR for thiophene-2,5-dicarboxamide alcohol-4b

4b_1H

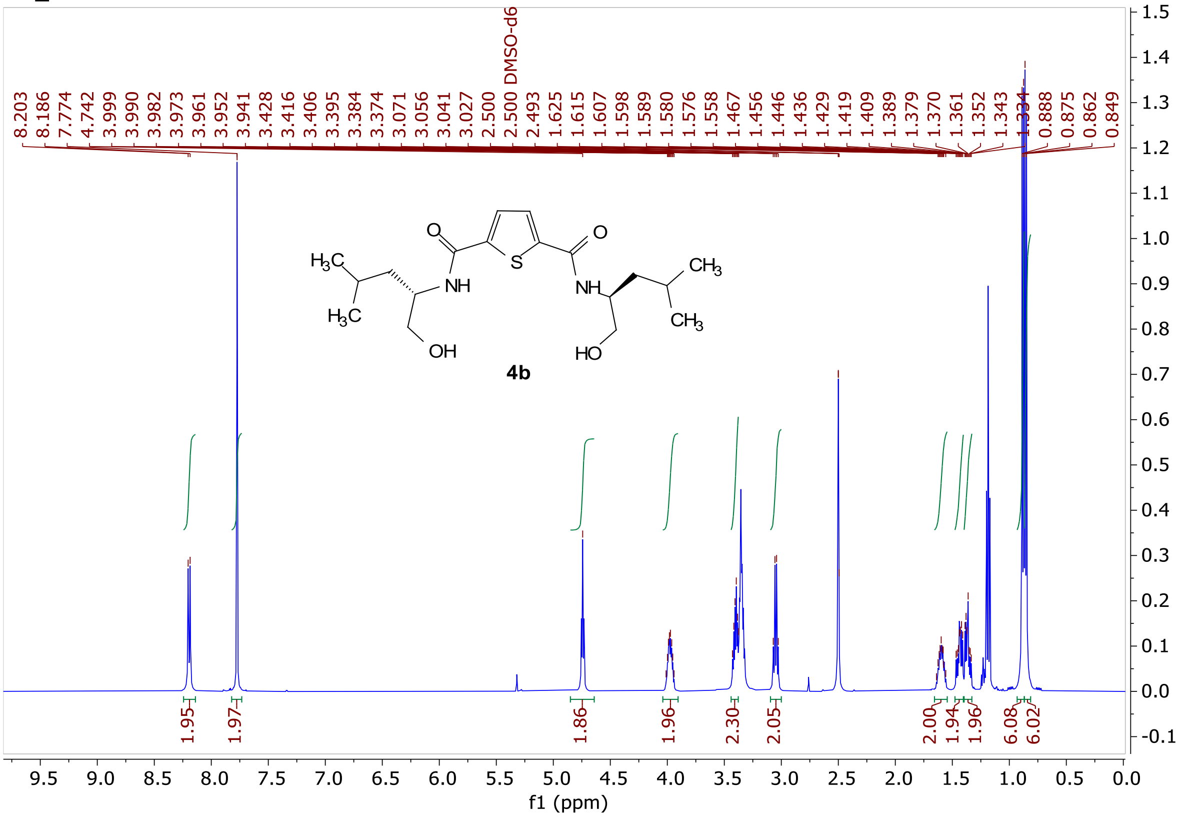

4b_13C

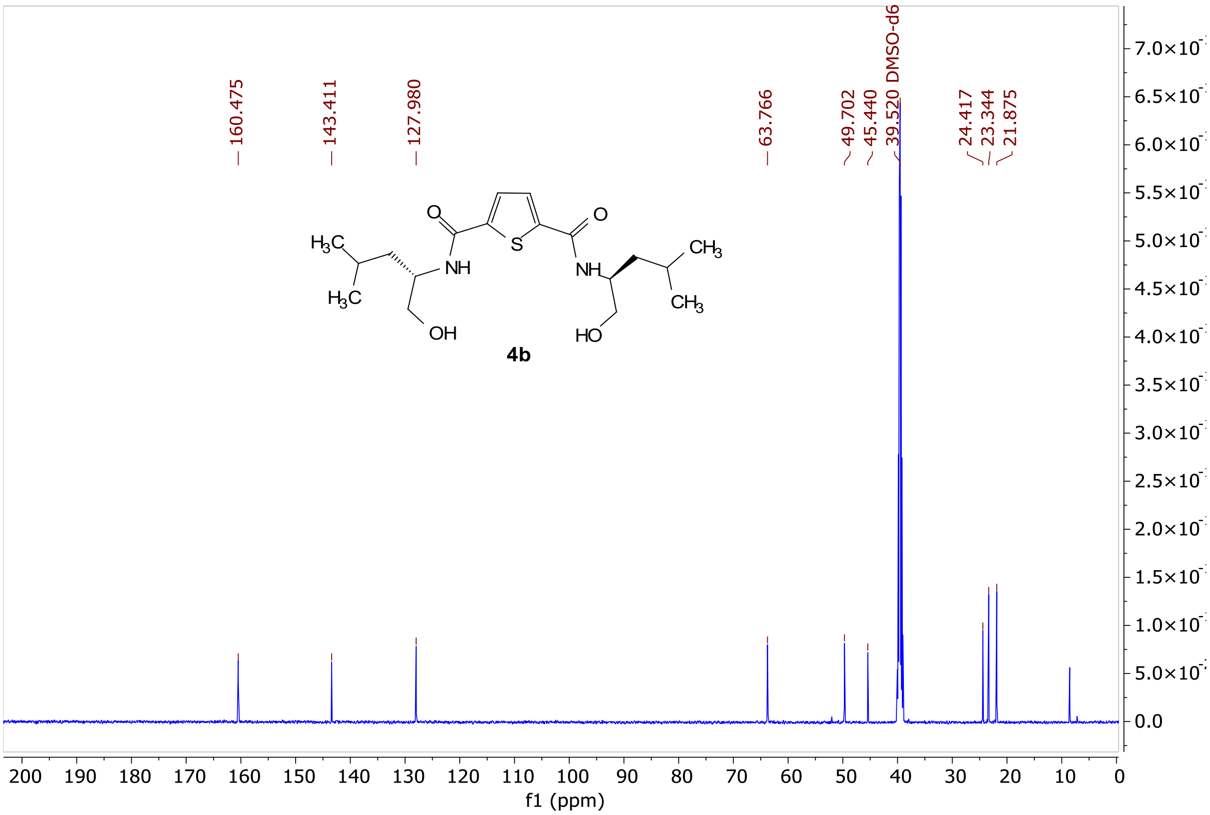

Electronic Supplementary file (ESI)

^{1}H NMR and ^{13}C-NMR for thiophene-2,5-dicarboxamide alcohol-4c

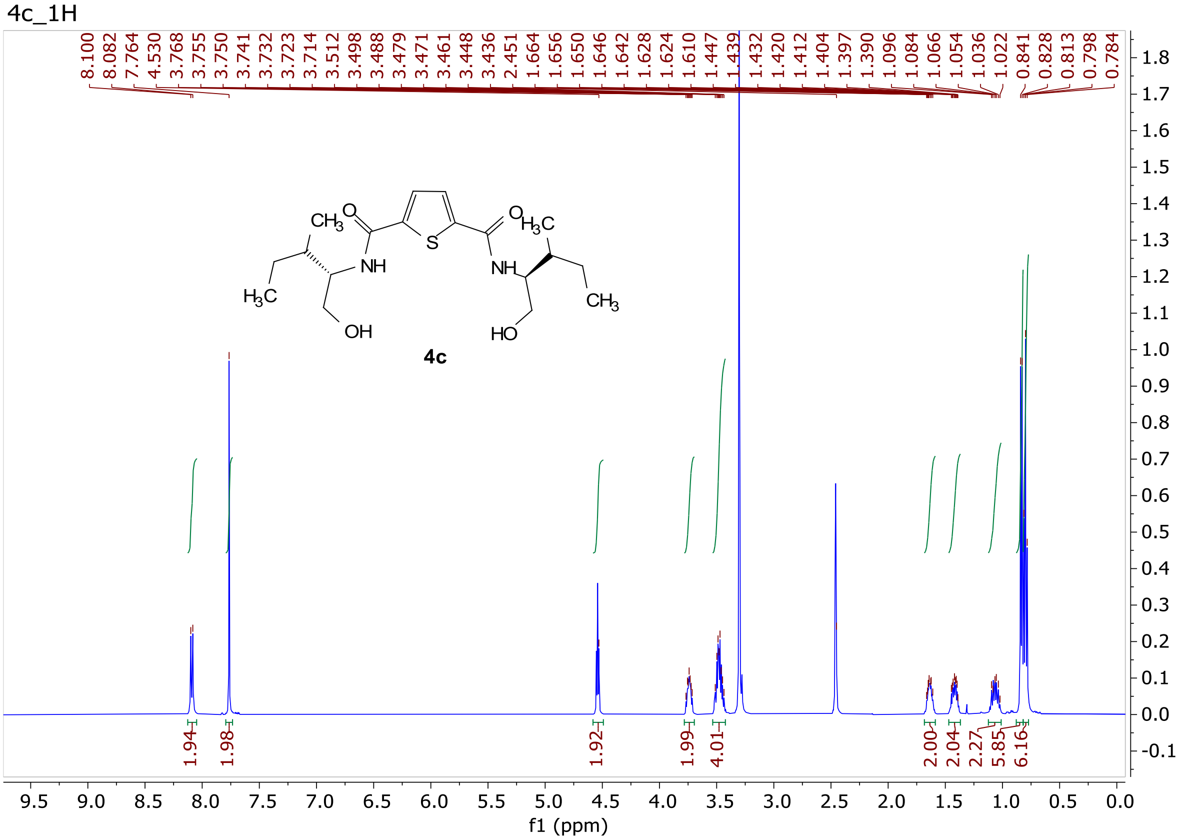

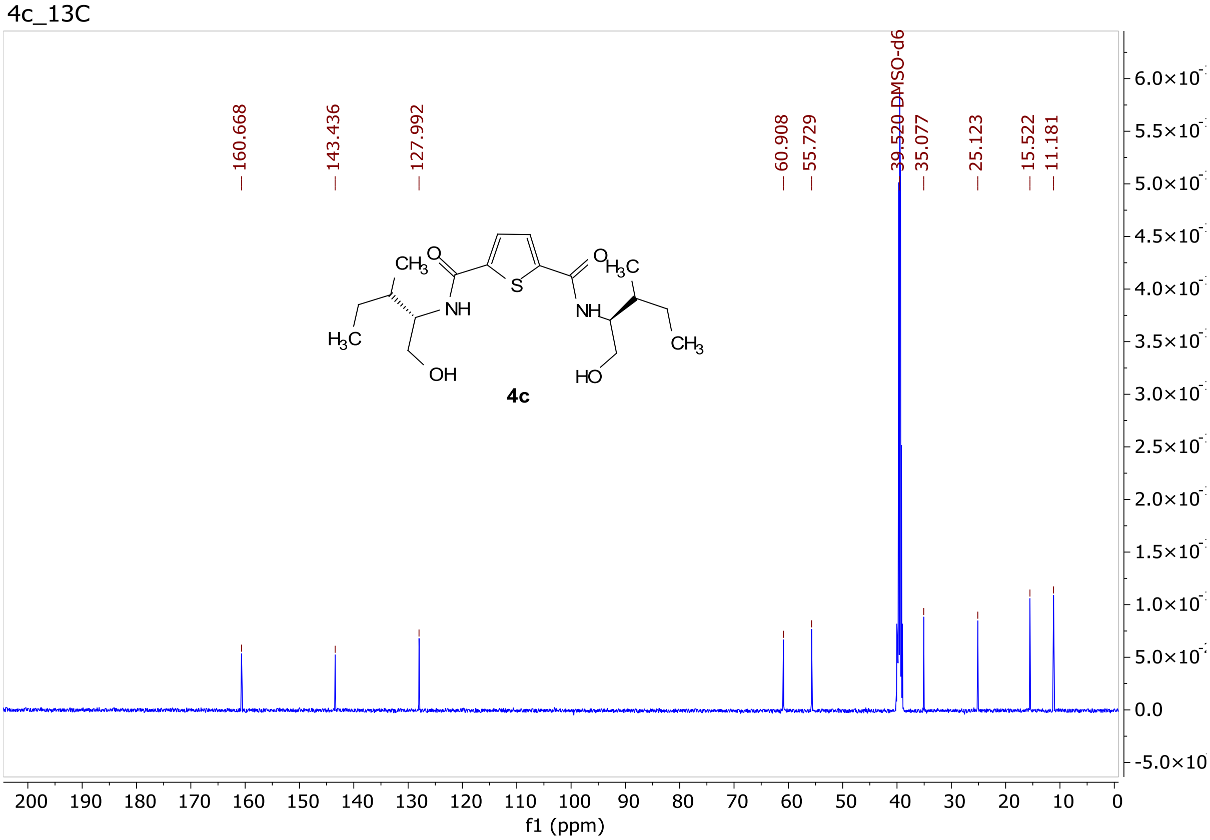

Electronic Supplementary file (ESI)

^{1}H NMR and ^{13}C-NMR for thiophene-2,5-bisimidazoline ligand-L1

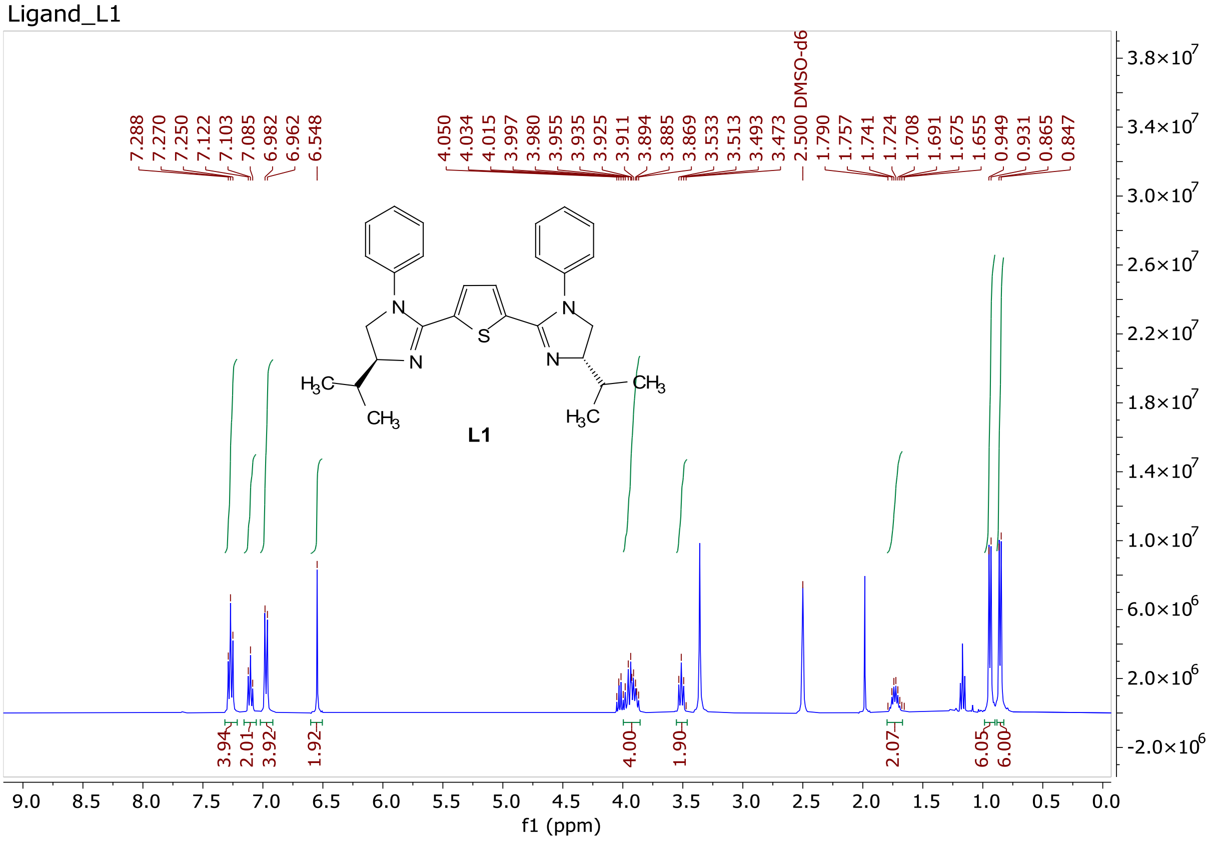

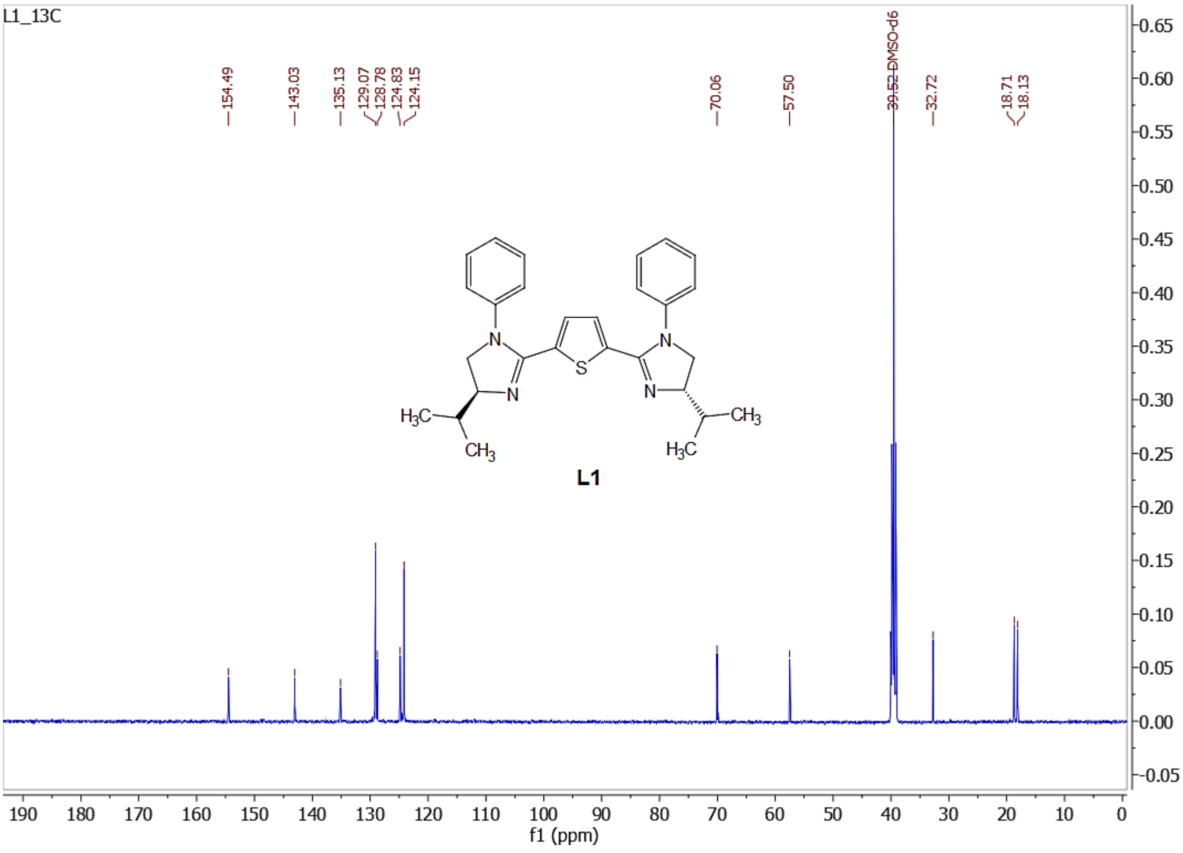

Electronic Supplementary file (ESI)

^{1}H NMR and ^{13}C-NMR for thiophene-2,5-bisimidazoline ligand-L2

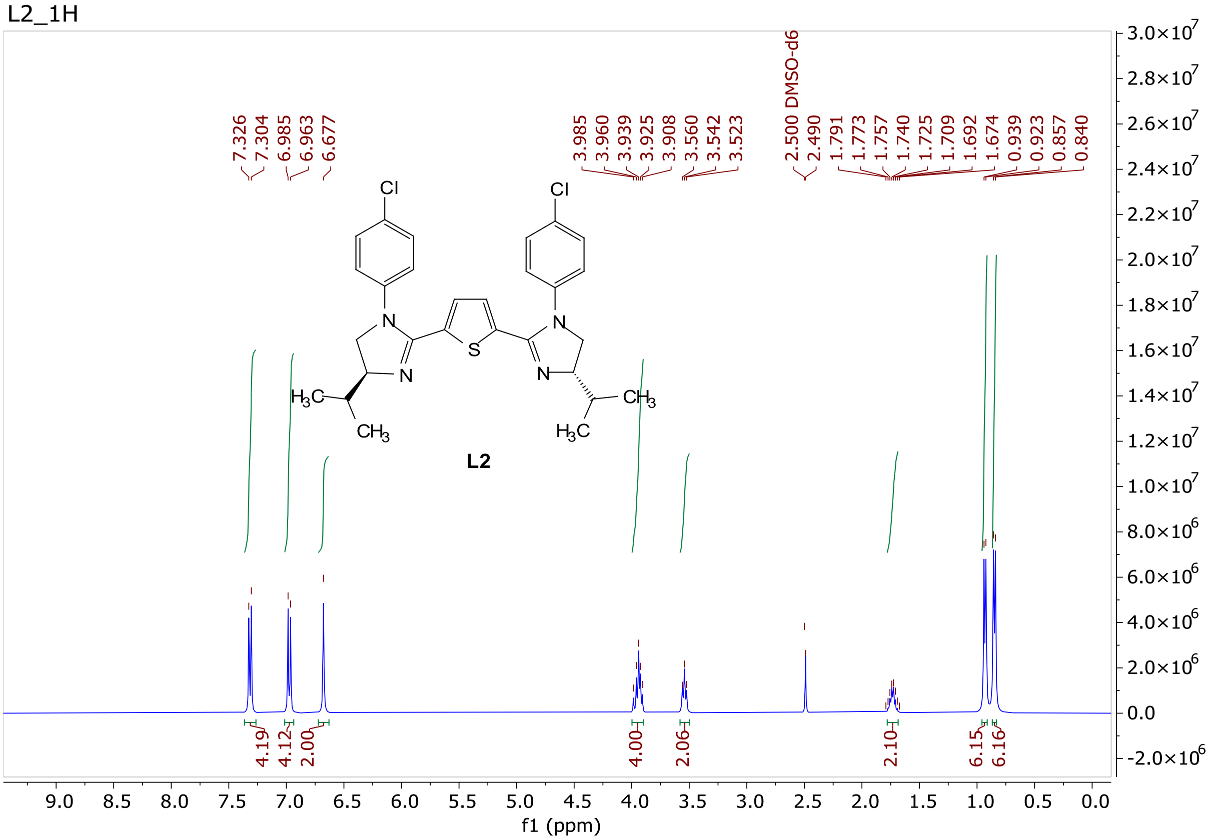

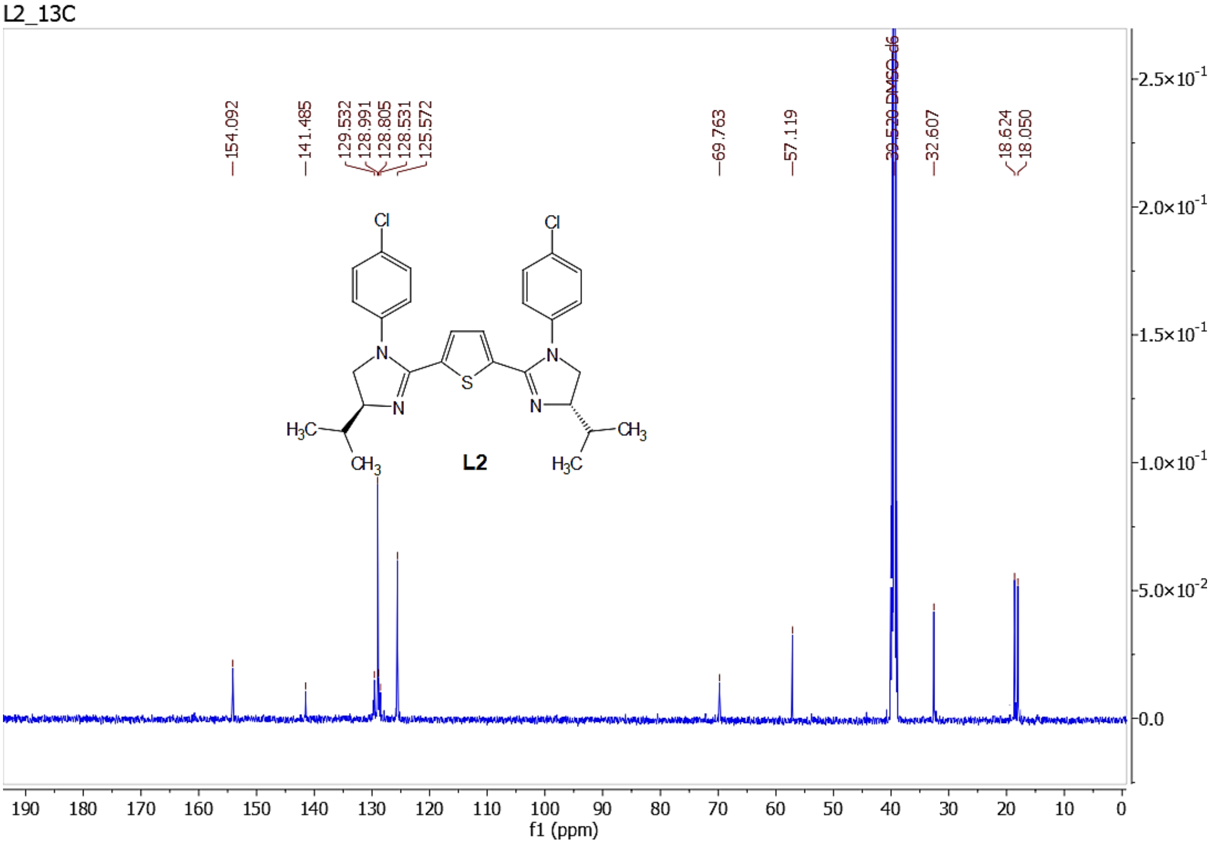

Electronic Supplementary file (ESI)

^{1}H NMR and ^{13}C-NMR for thiophene-2,5-bisimidazoline ligand-L3

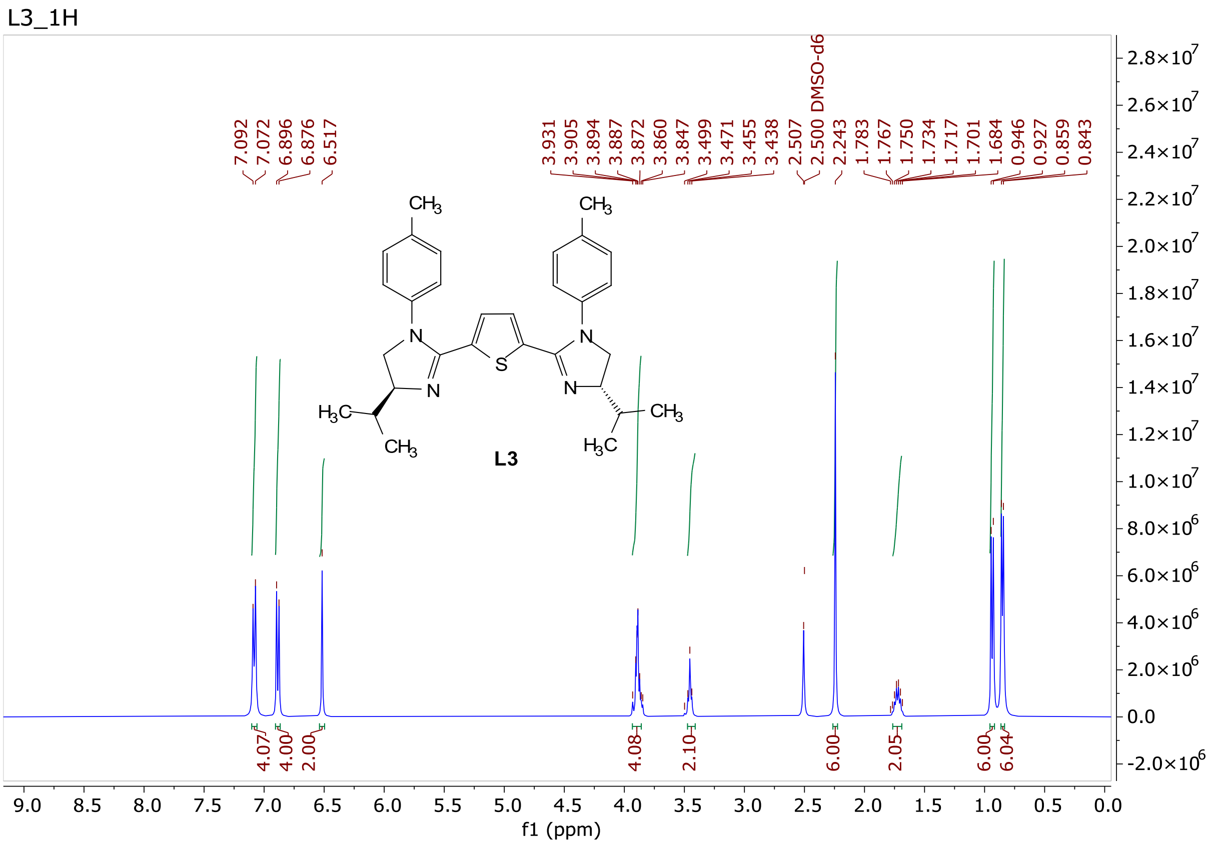

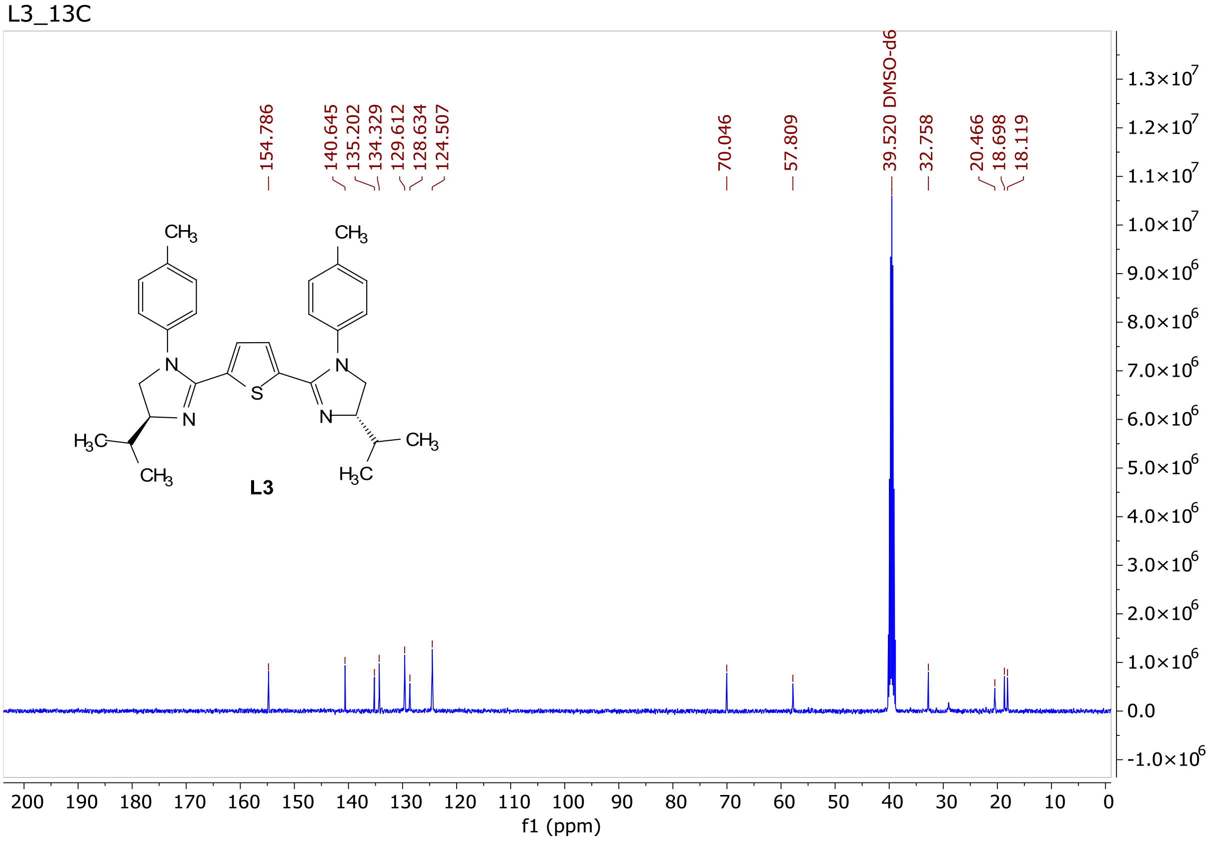

Electronic Supplementary file (ESI)

^{1}H NMR and ^{13}C-NMR for thiophene-2,5-bisoxazoline ligand-L4

L4_1H

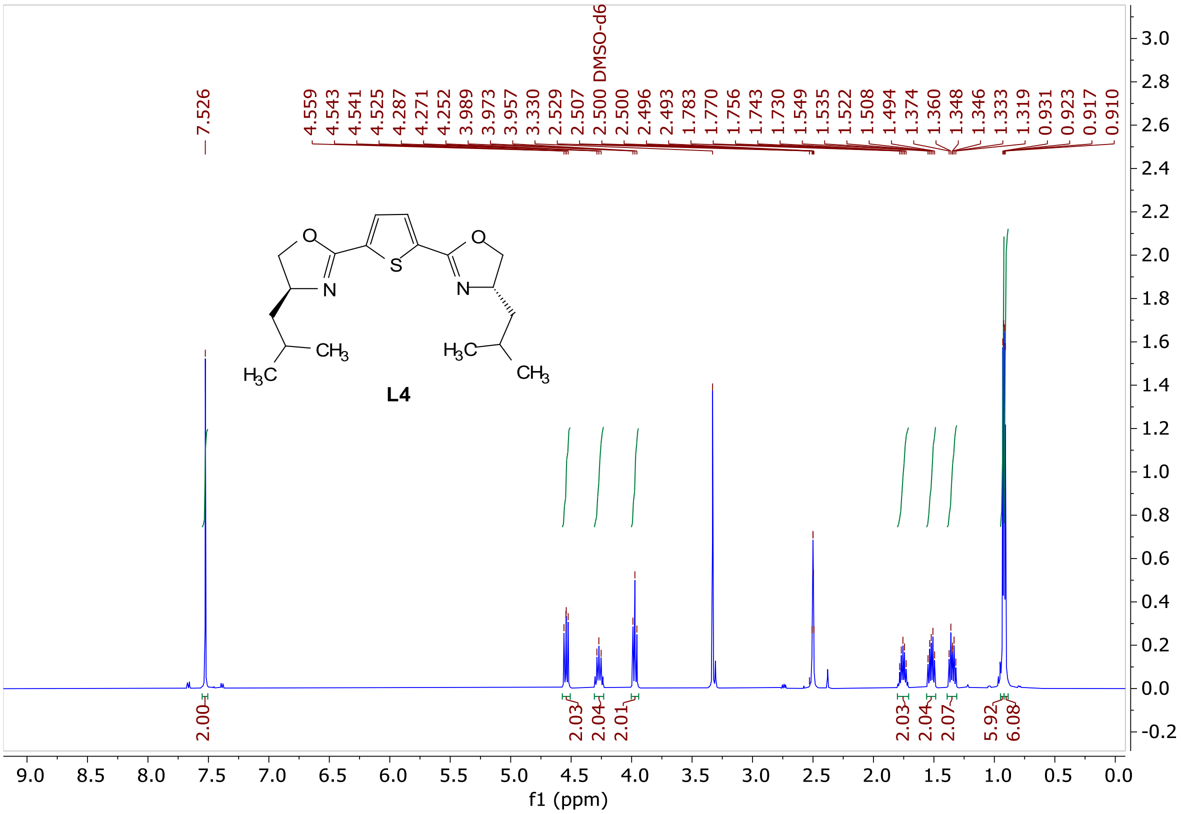

L4_13C

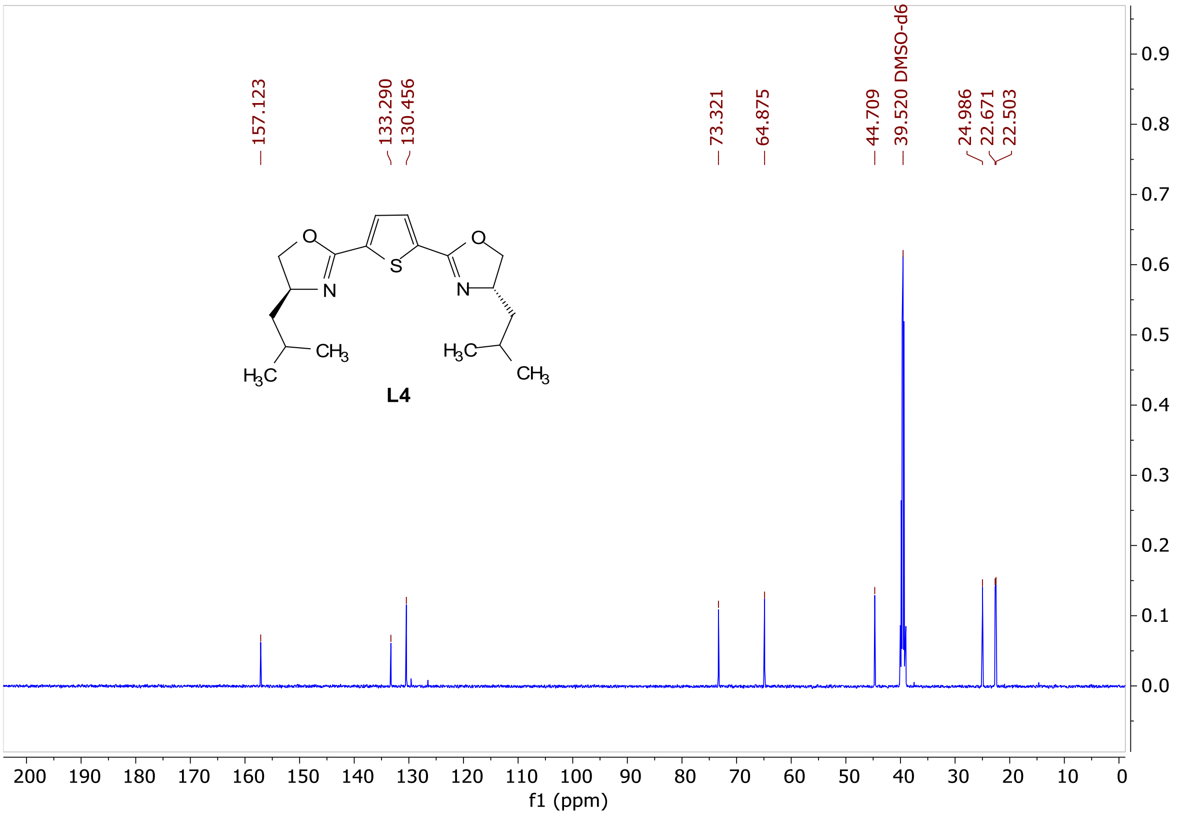

^{1}H NMR and ^{13}C-NMR for thiophene-2,5-bisoxazoline ligand-L5

L5_1H

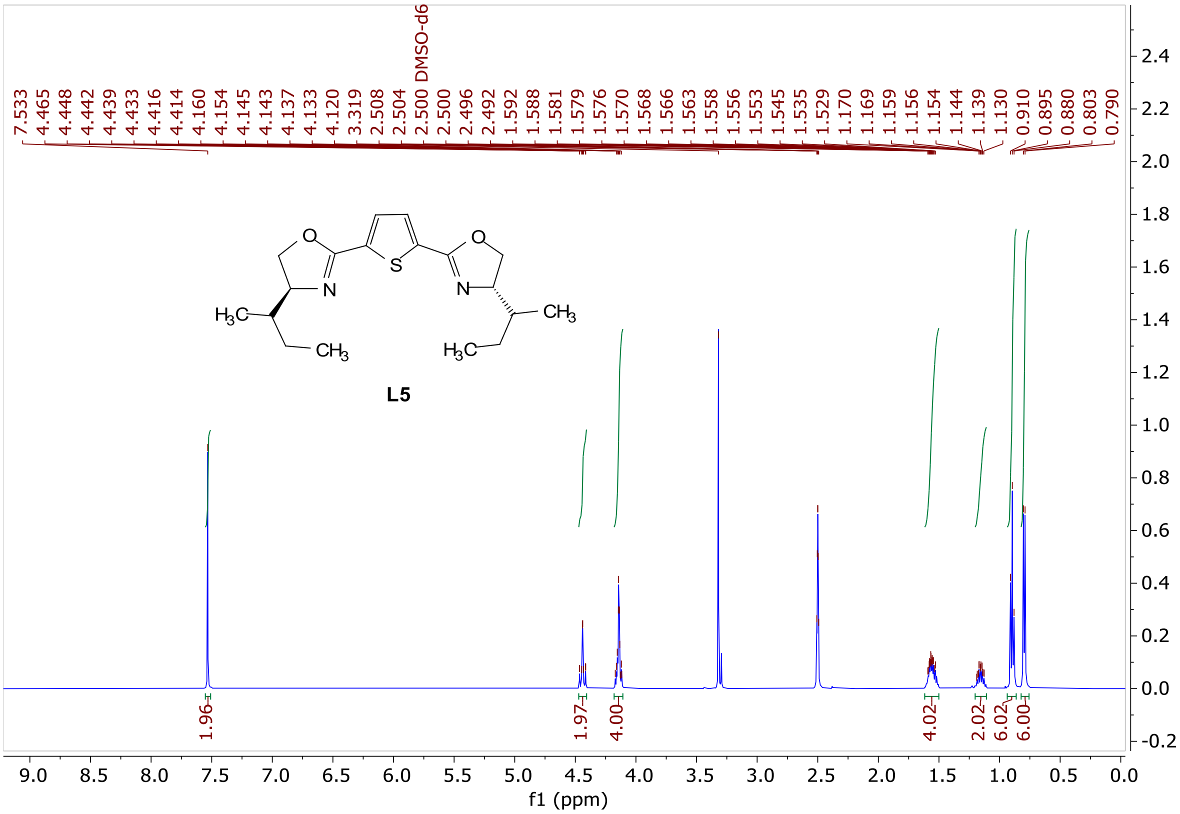

L5_13C

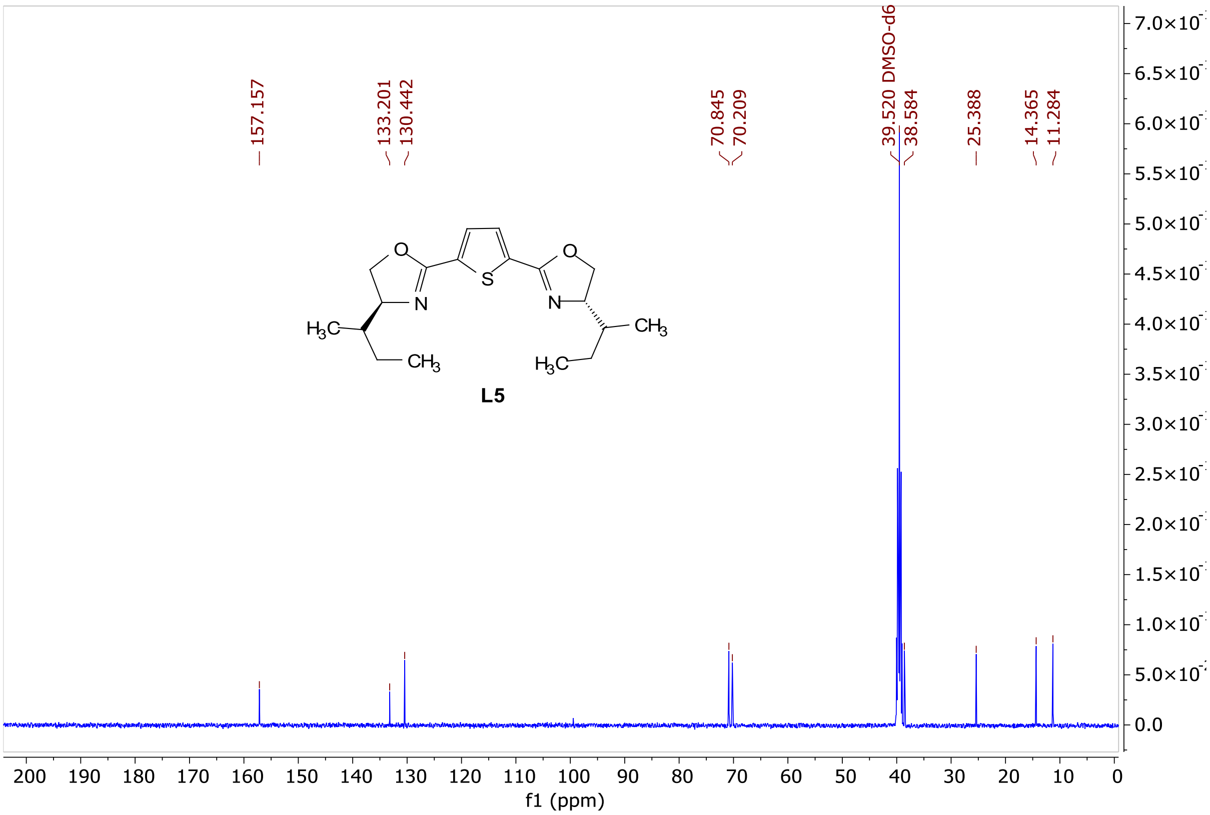

^{1}H NMR and ^{13}C-NMR for Friedel Craft product-10a

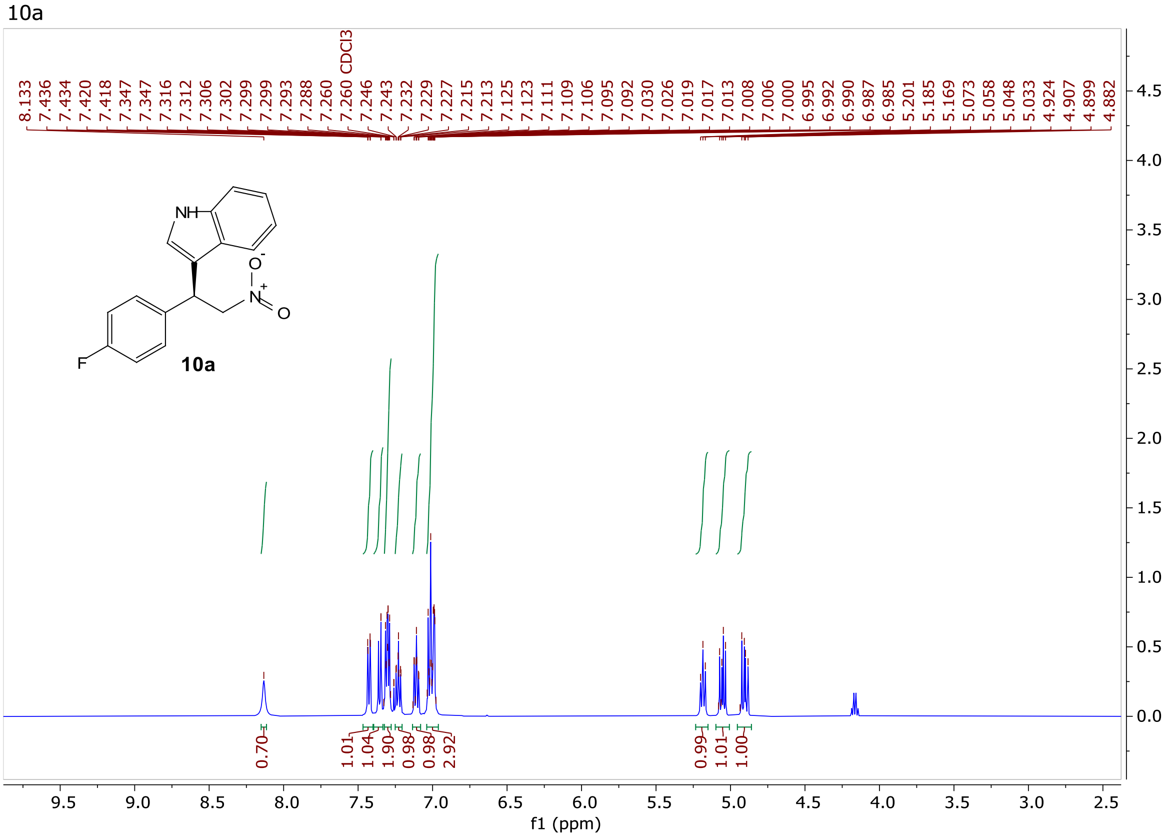

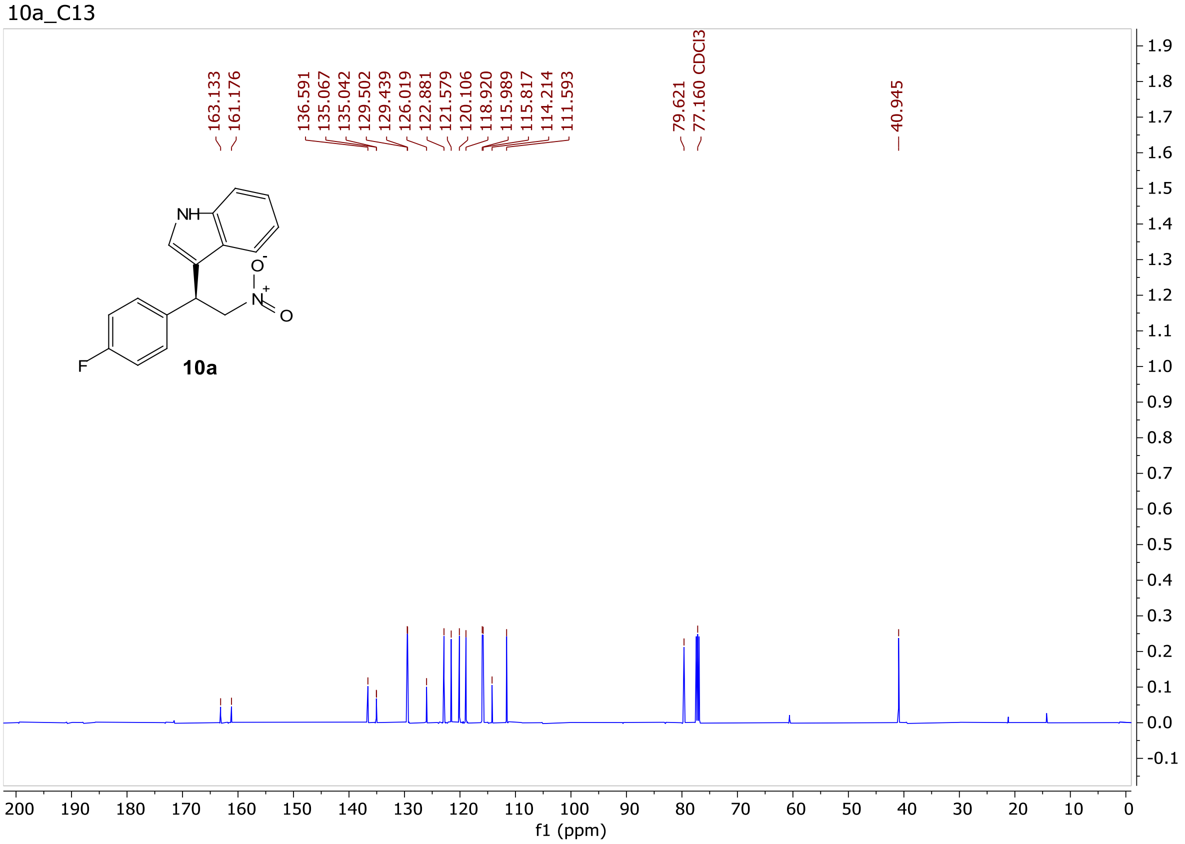

Electronic Supplementary file (ESI)

^{1}H NMR and ^{13}C-NMR for Friedel Craft product-10b

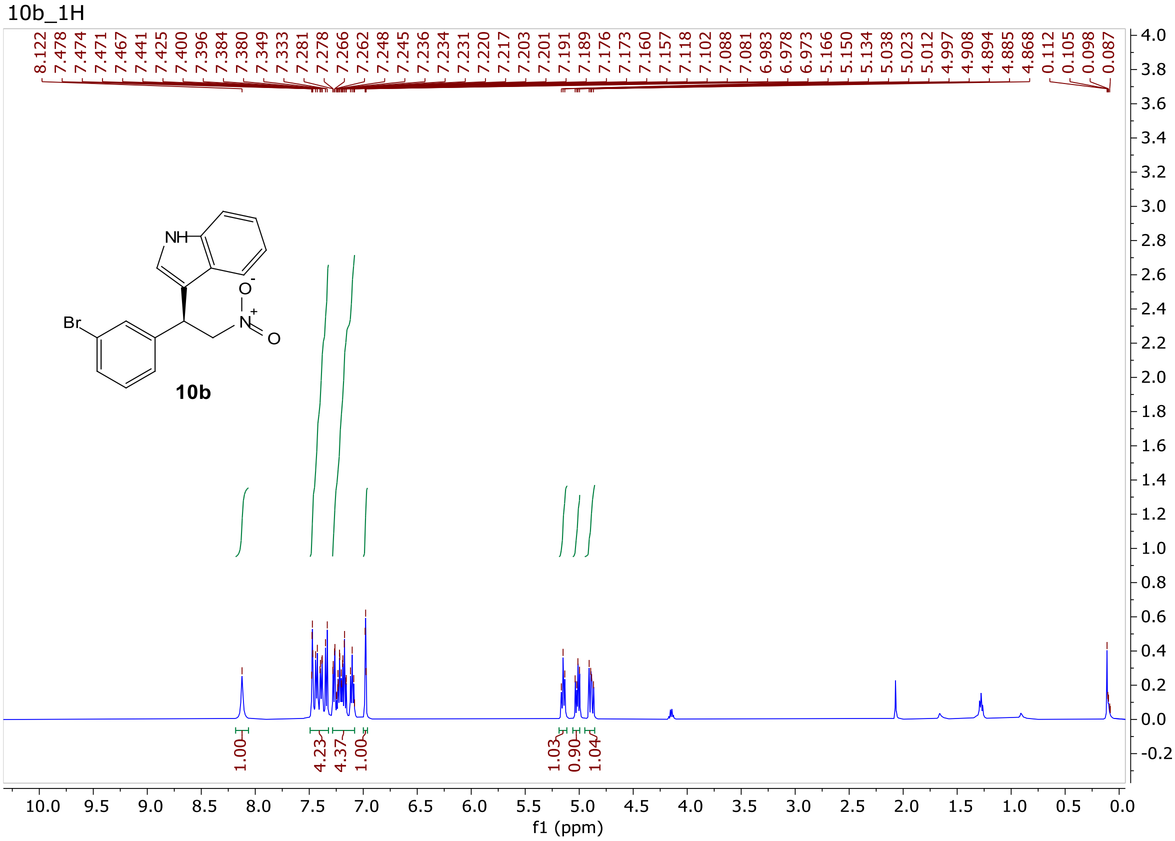

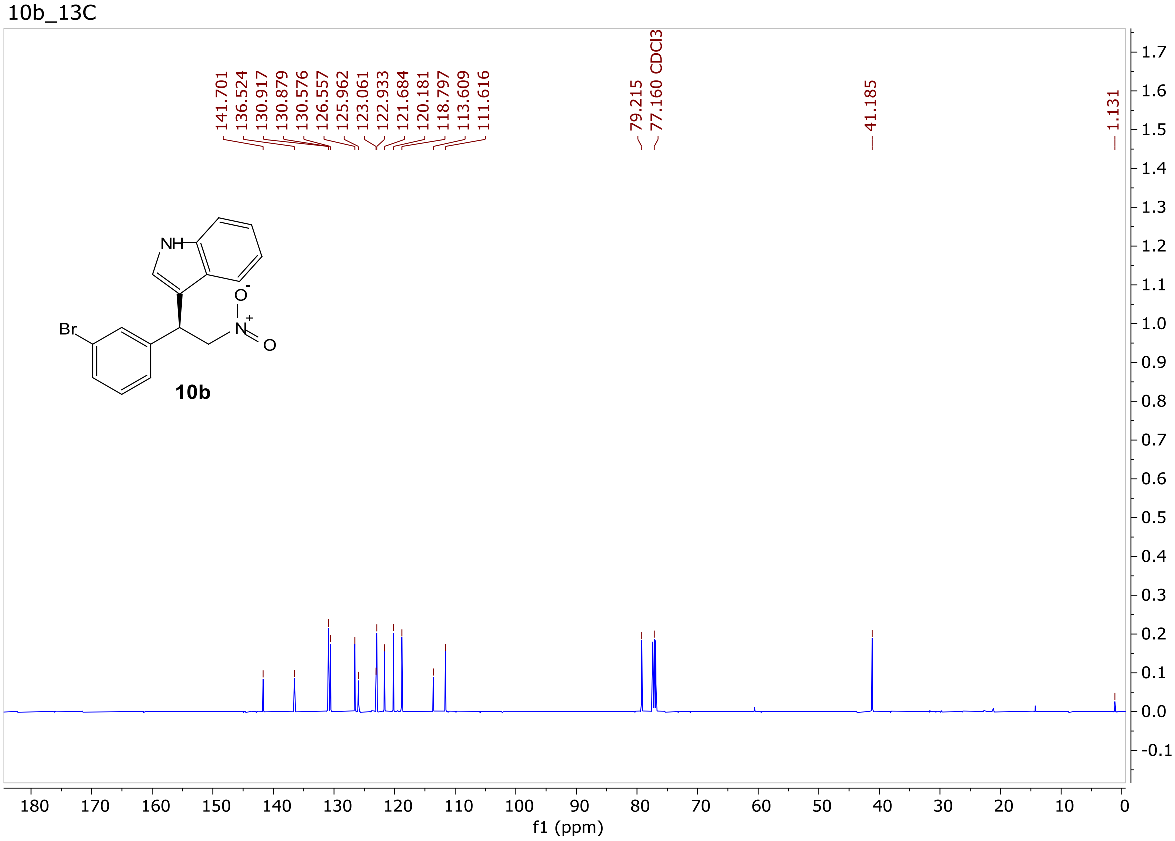

^{1}H NMR and ^{13}C-NMR for Friedel Craft product-10c

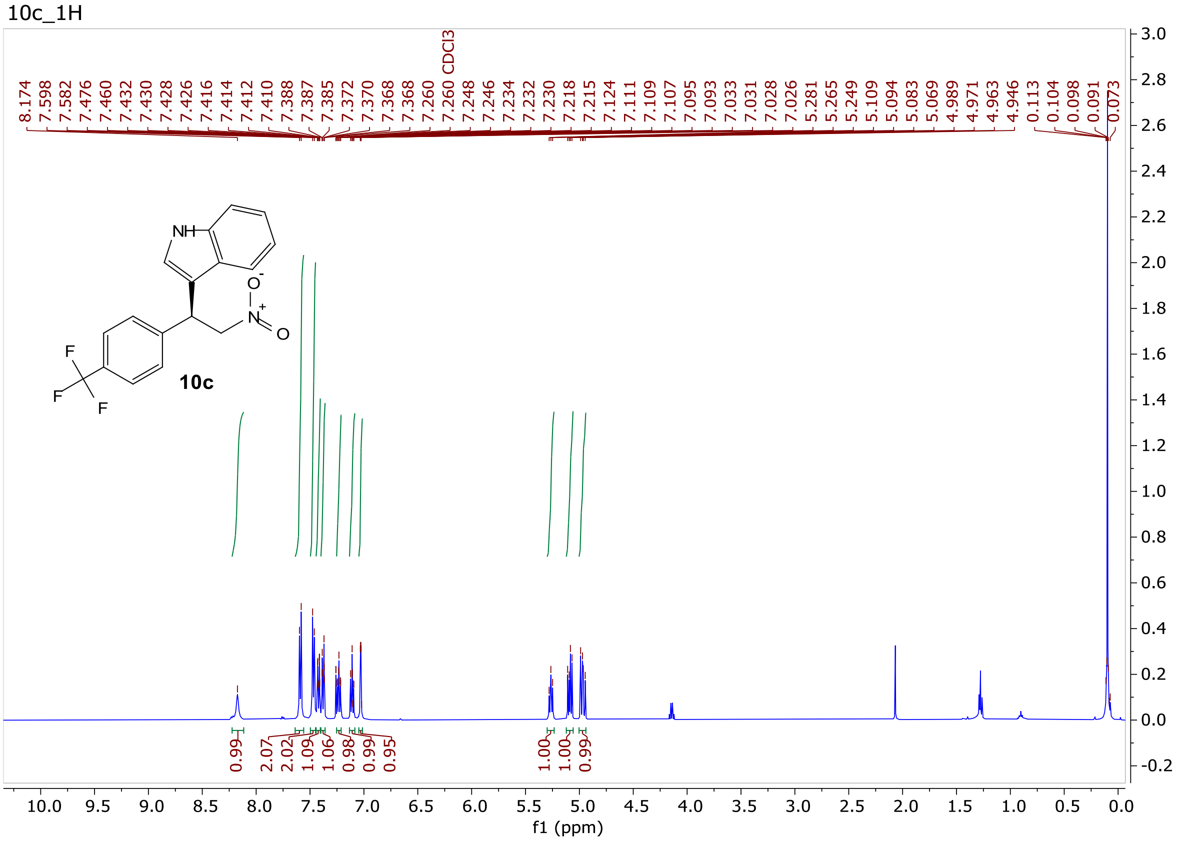

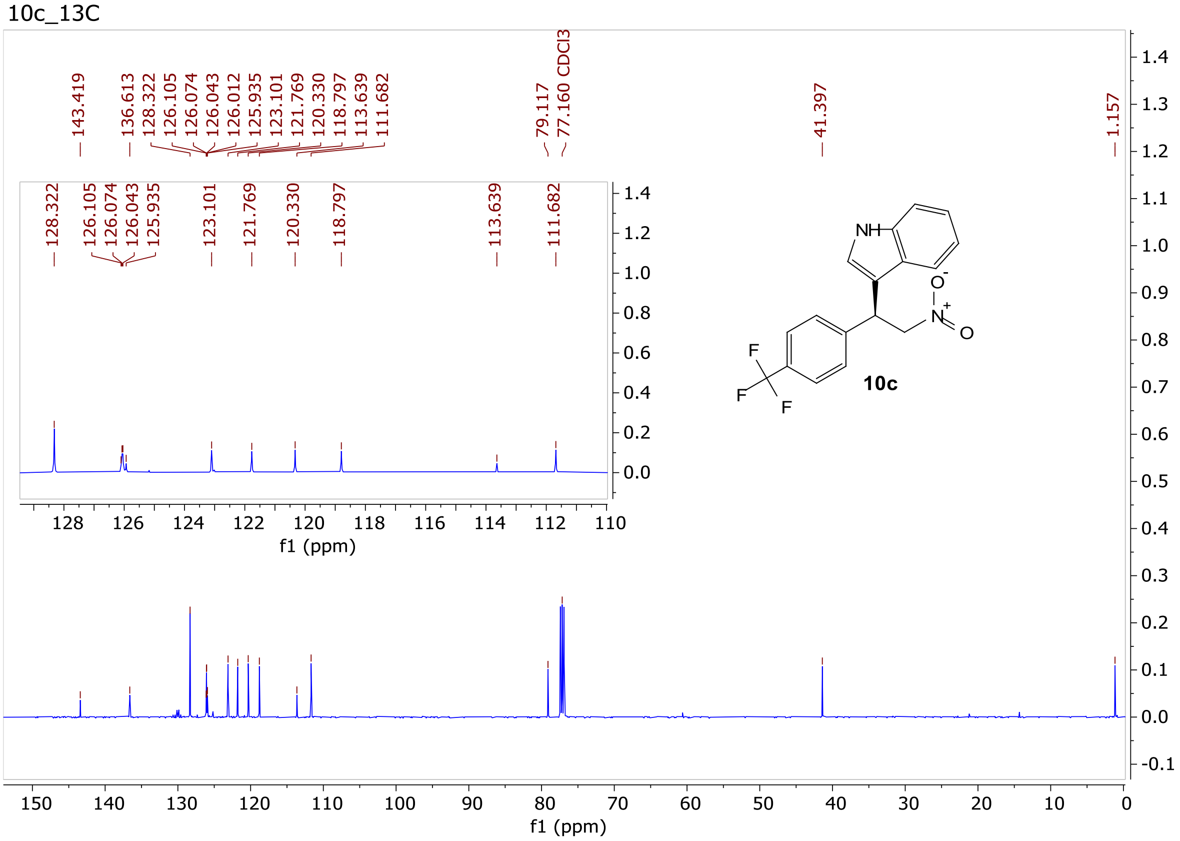

Electronic Supplementary file (ESI)

^{1}H NMR and ^{13}C-NMR for Friedel Craft product-10d

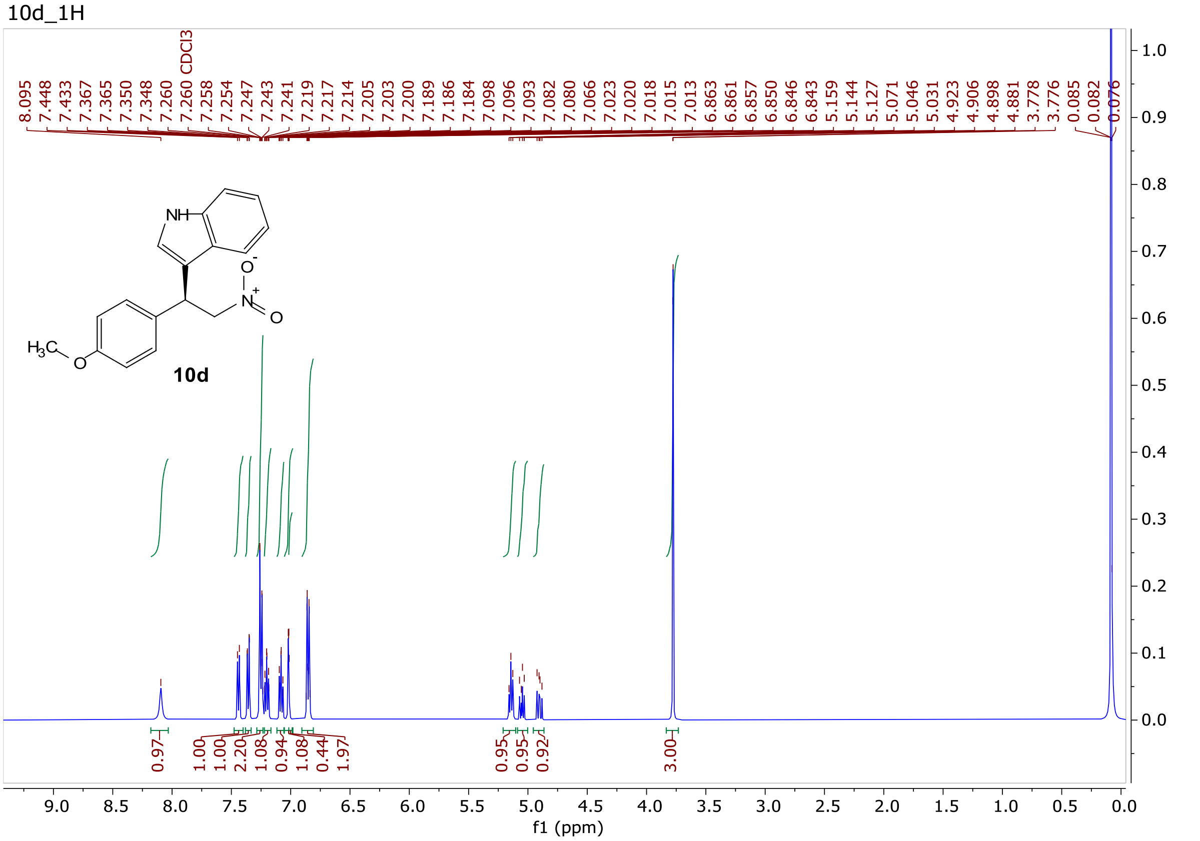

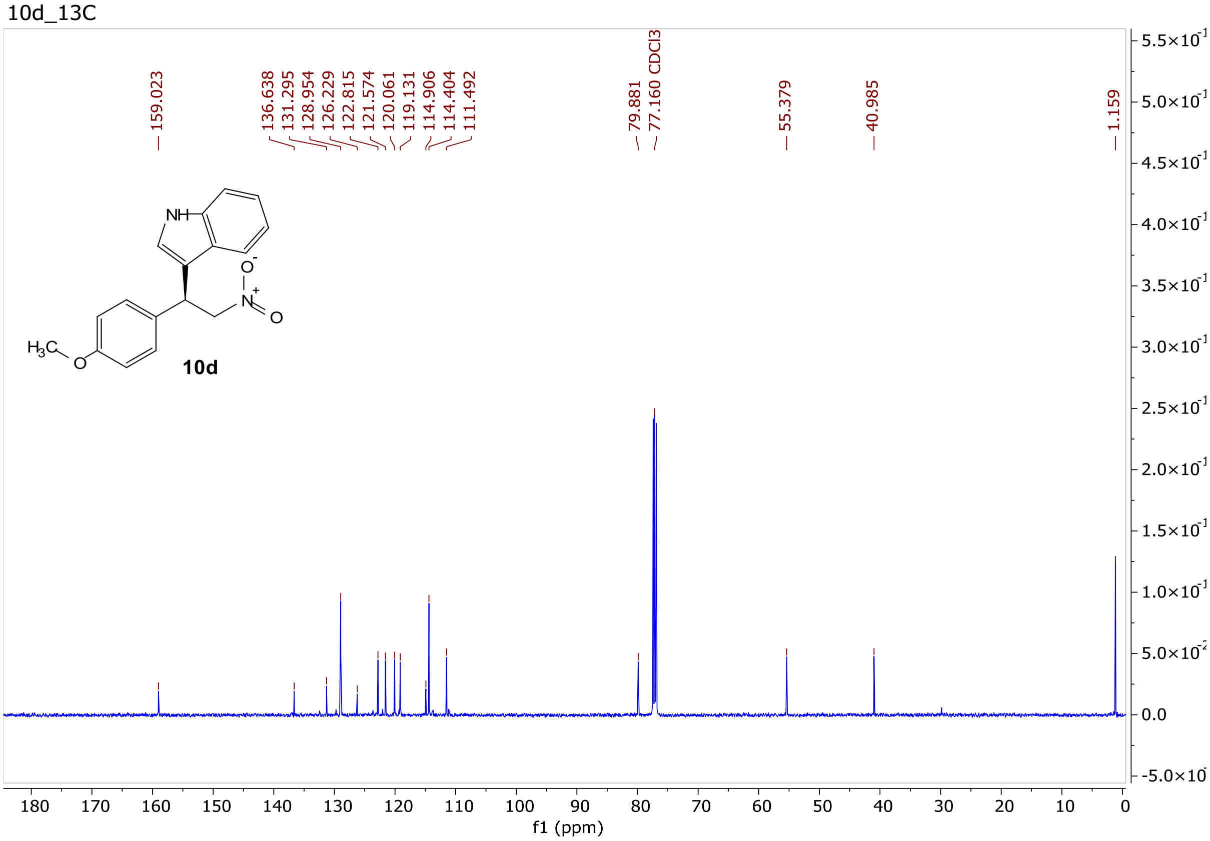

Electronic Supplementary file (ESI)

^{1}H NMR and ^{13}C-NMR for Friedel Craft product-10e

10e_1H

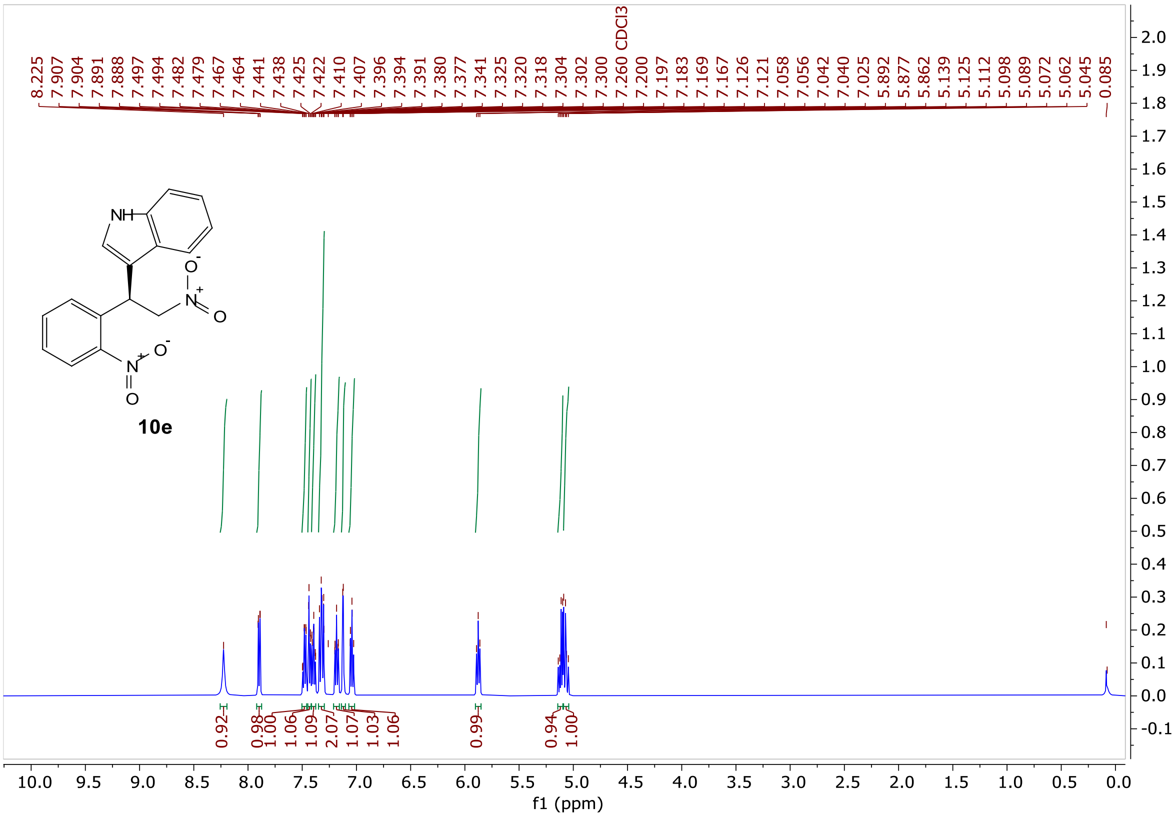

10e_13C

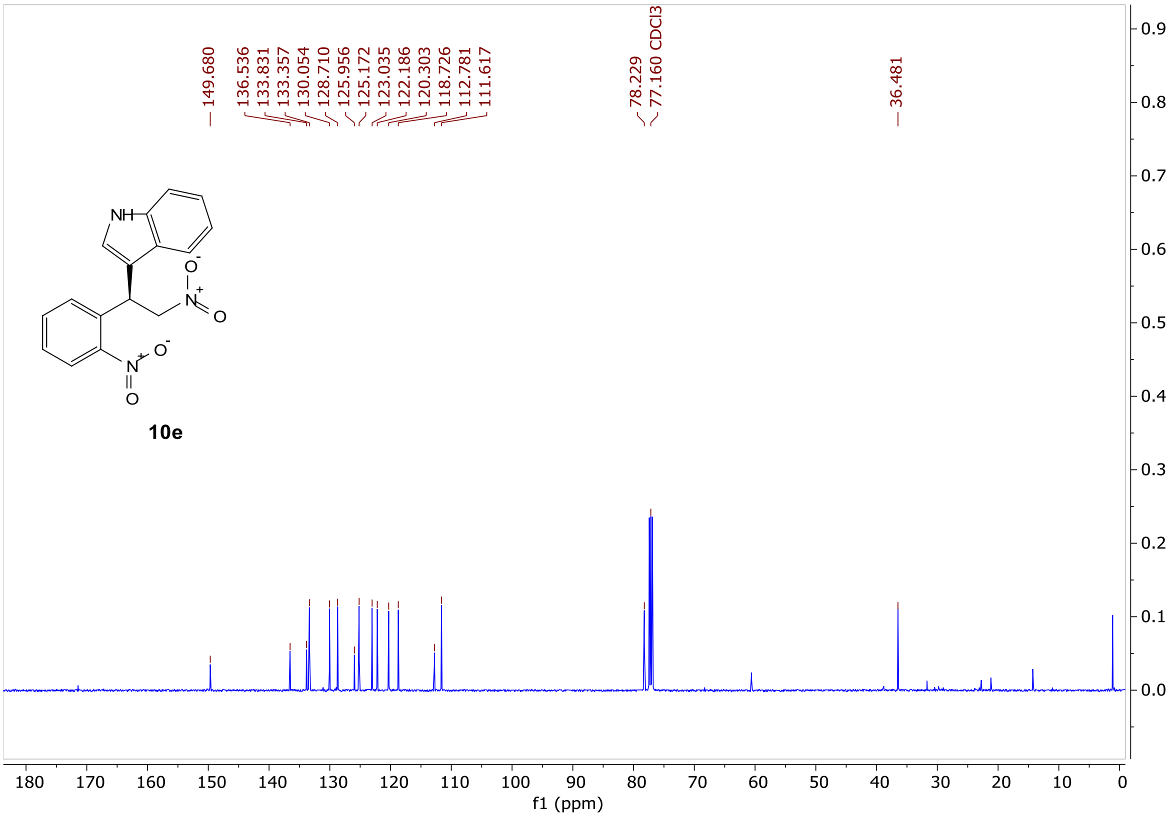

^{1}H NMR and ^{13}C-NMR for Friedel Craft product-10f

10f_1H

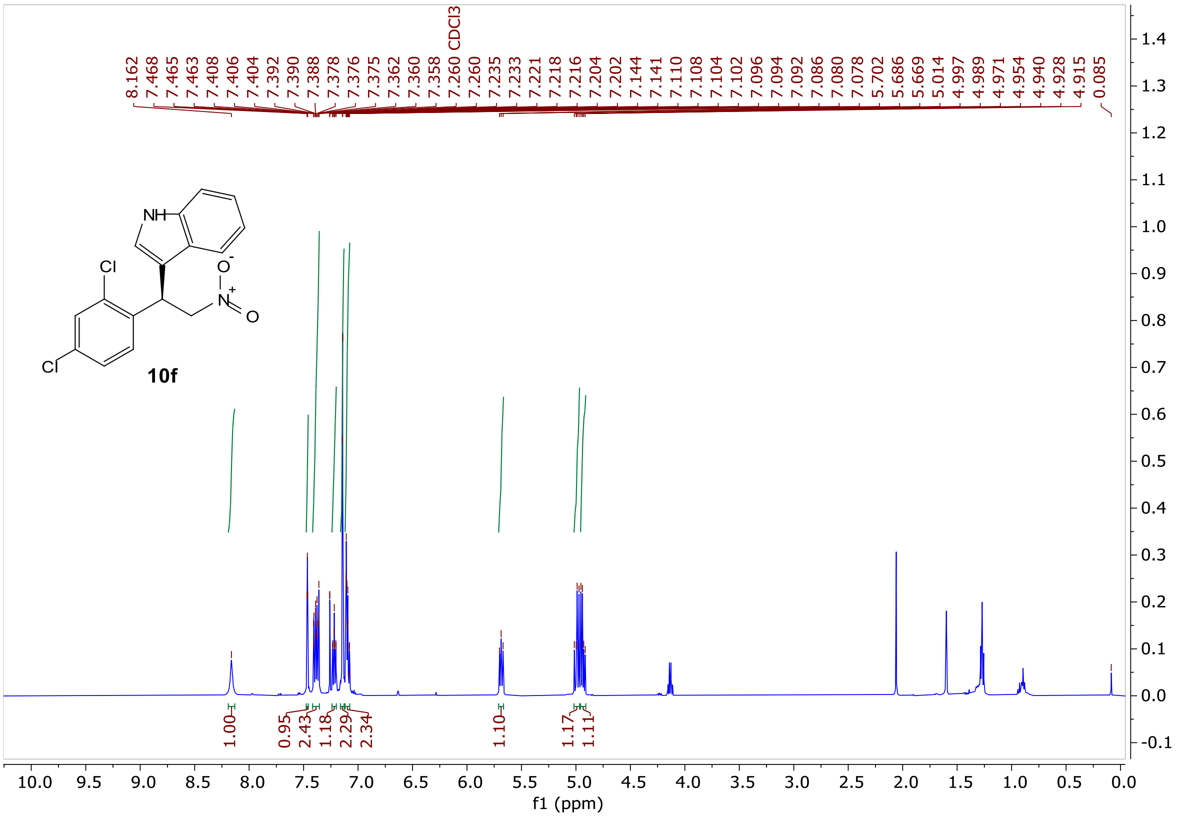

10f_13C

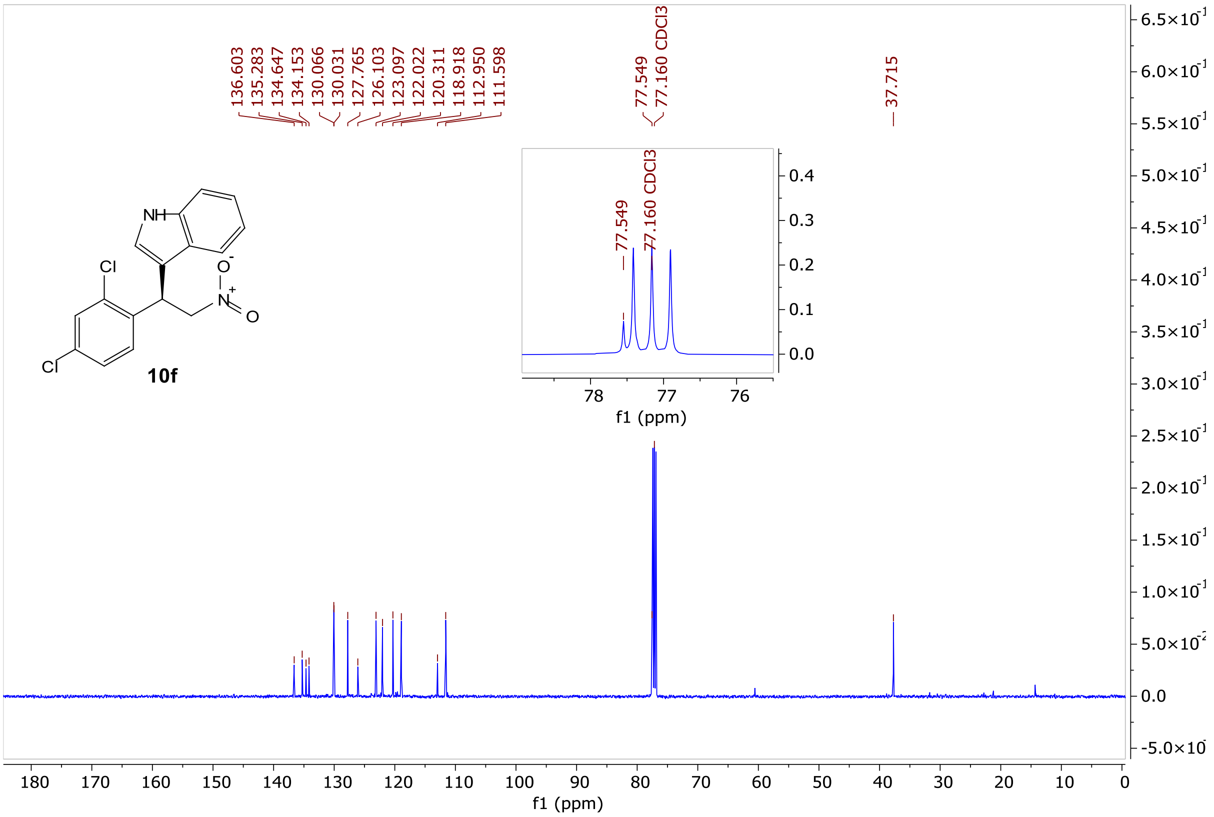

^{1}H NMR and ^{13}C-NMR for Friedel Craft product-10g

10g_1H

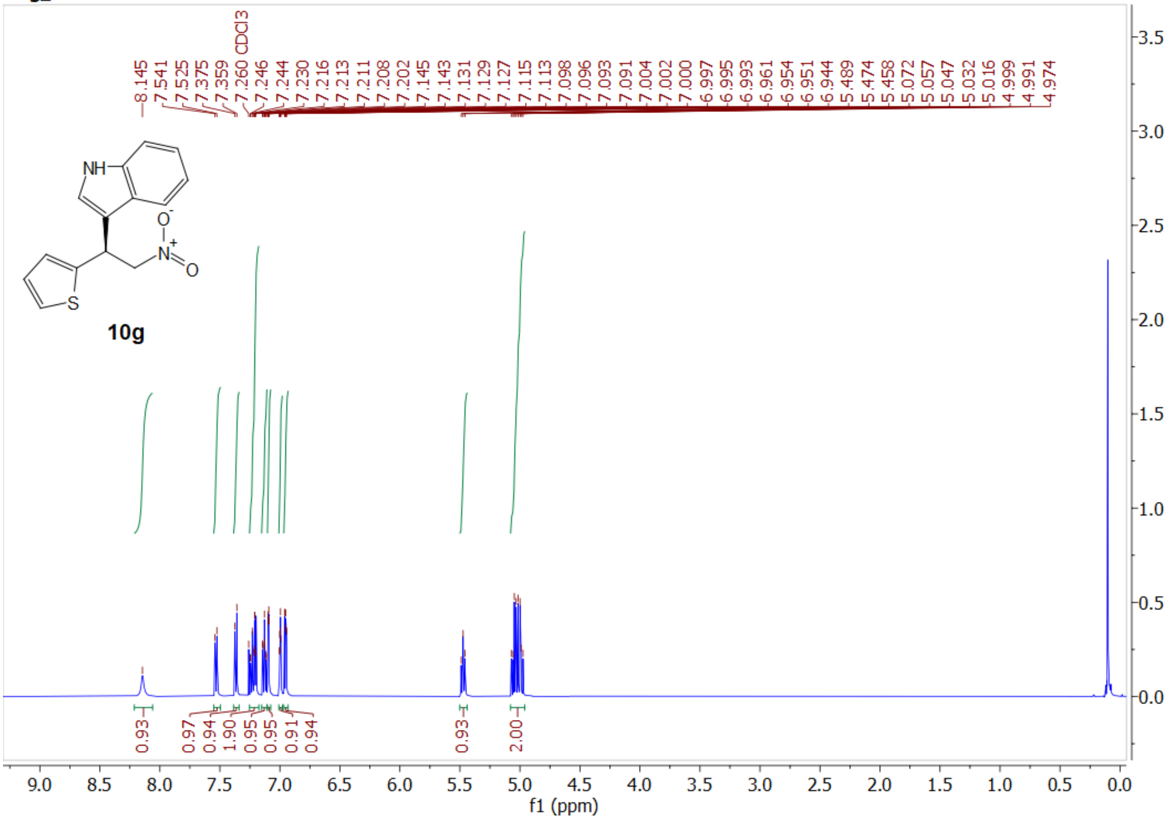

10g_13C

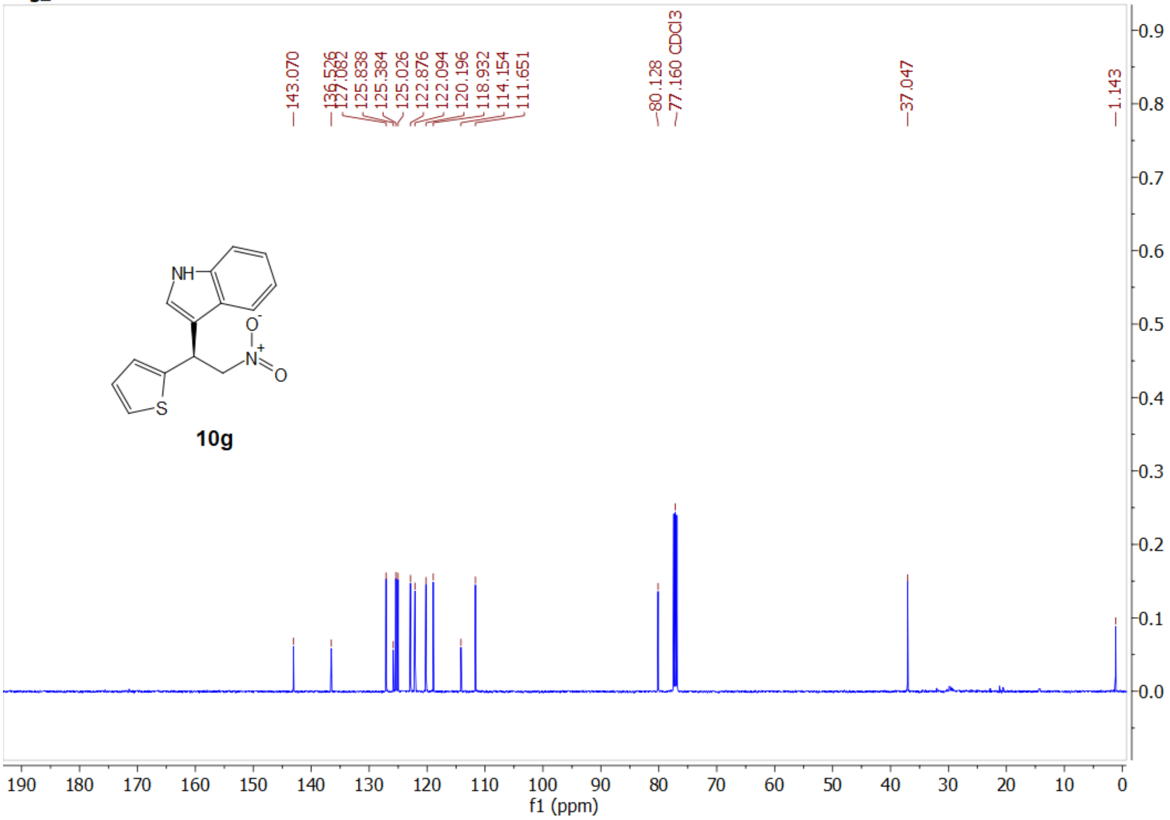

^{1}H NMR and ^{13}C-NMR for Friedel Craft product-10h

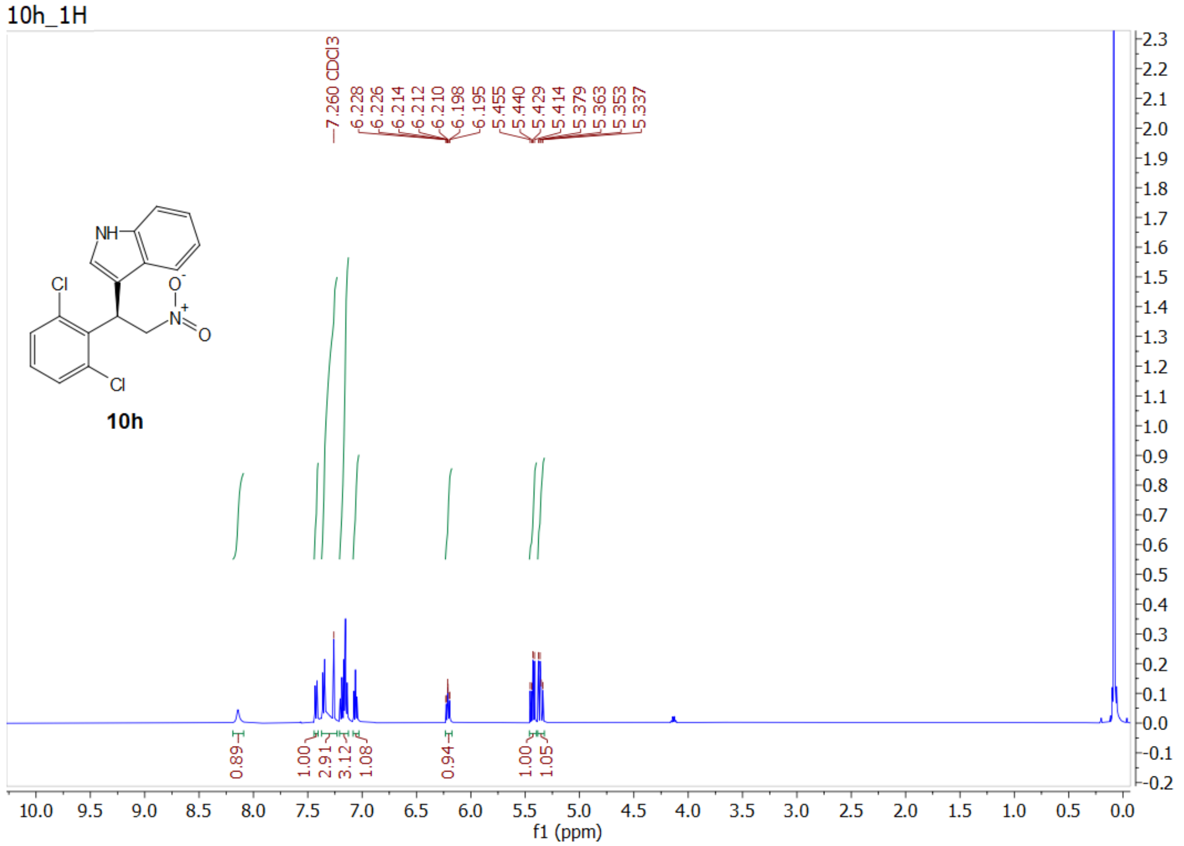

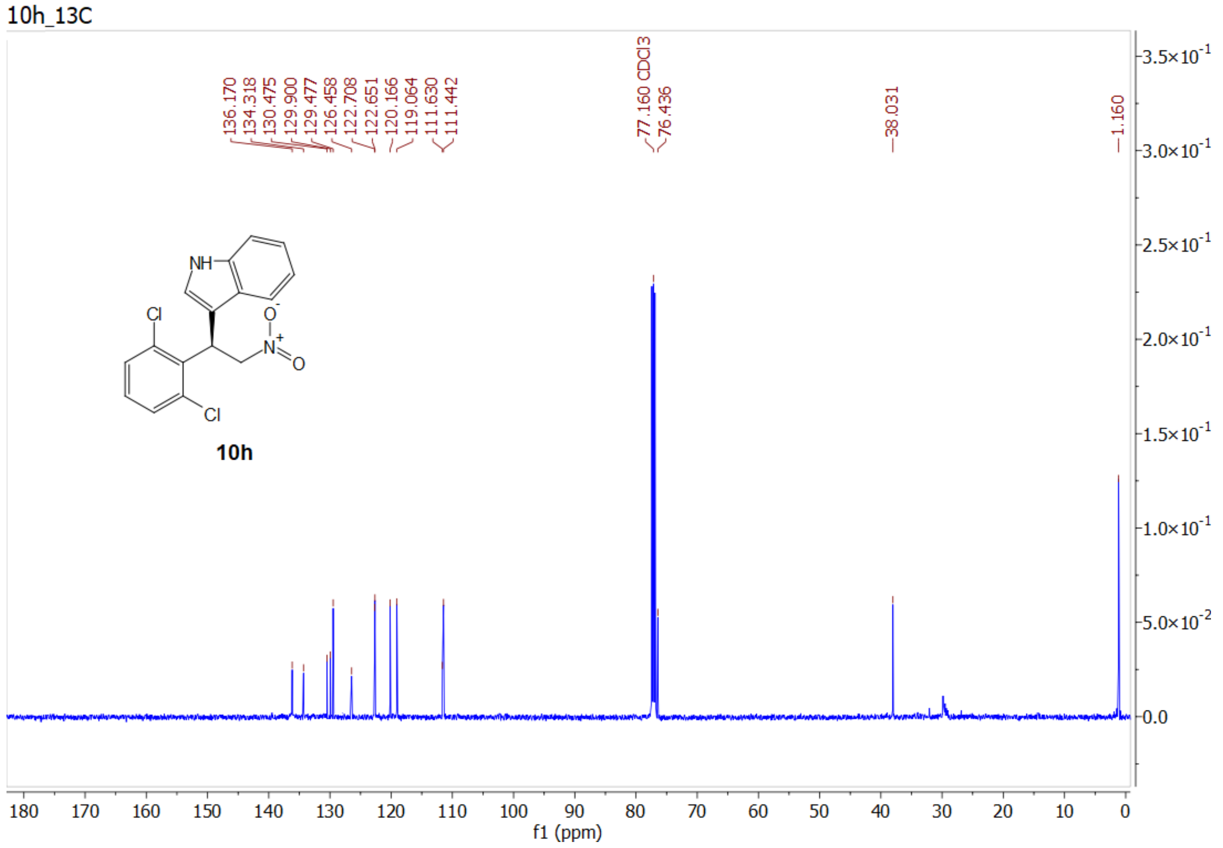

Electronic Supplementary file (ESI)

^{1}H NMR and ^{13}C-NMR for Friedel Craft product-10i

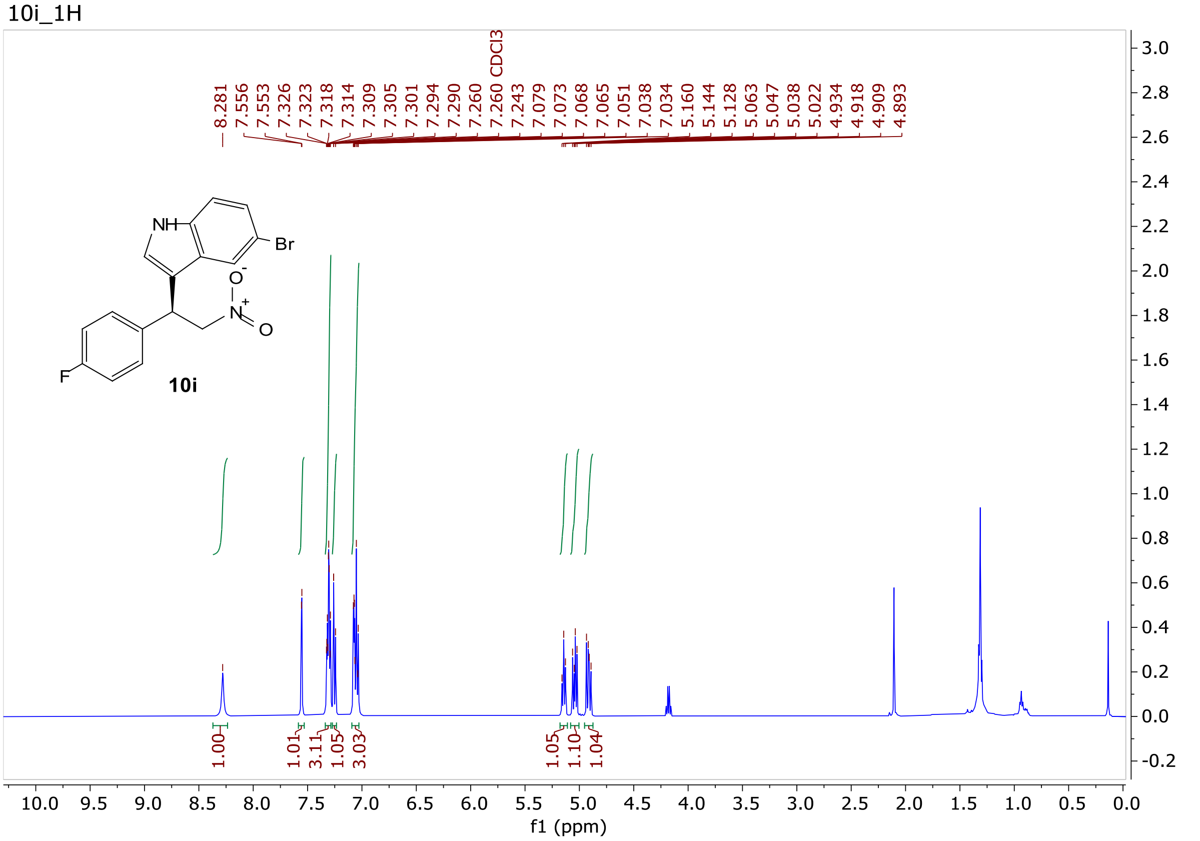

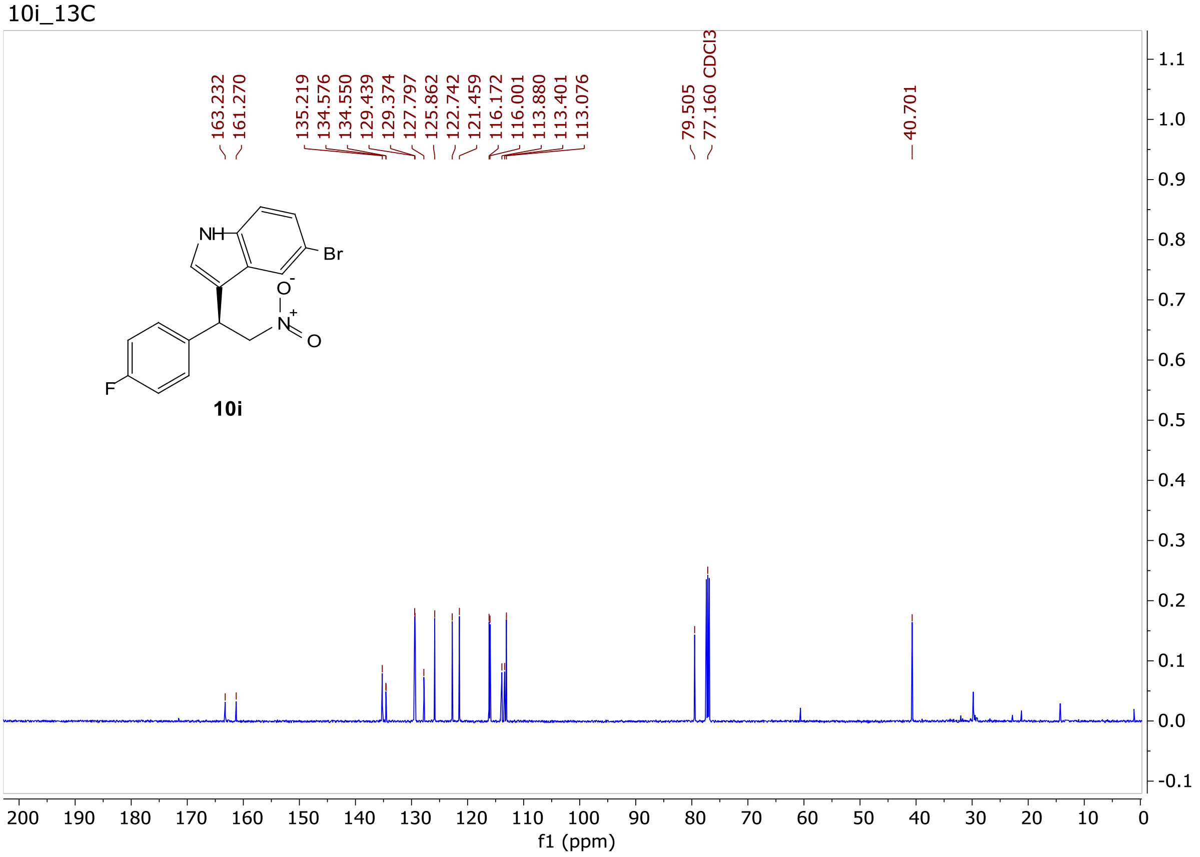

Electronic Supplementary file (ESI)

^{1}H NMR and ^{13}C-NMR for Friedel Craft product-10j

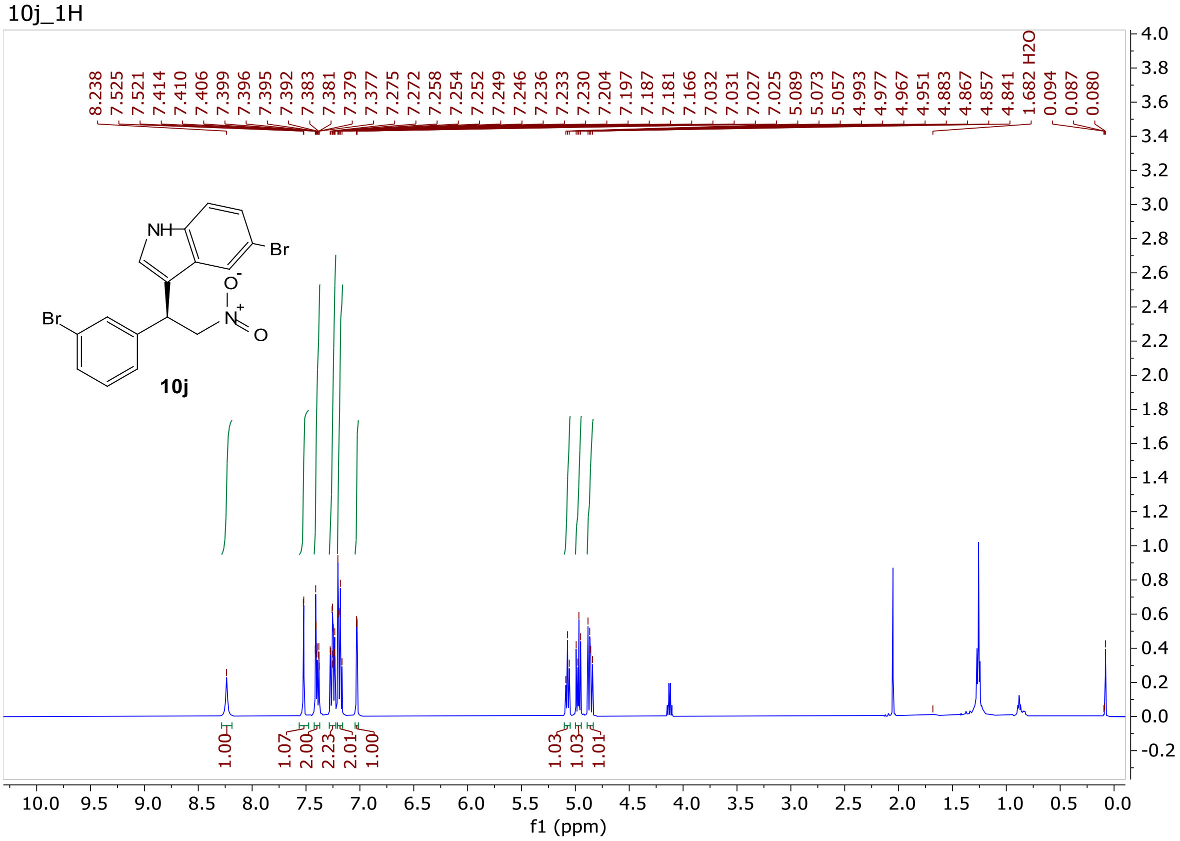

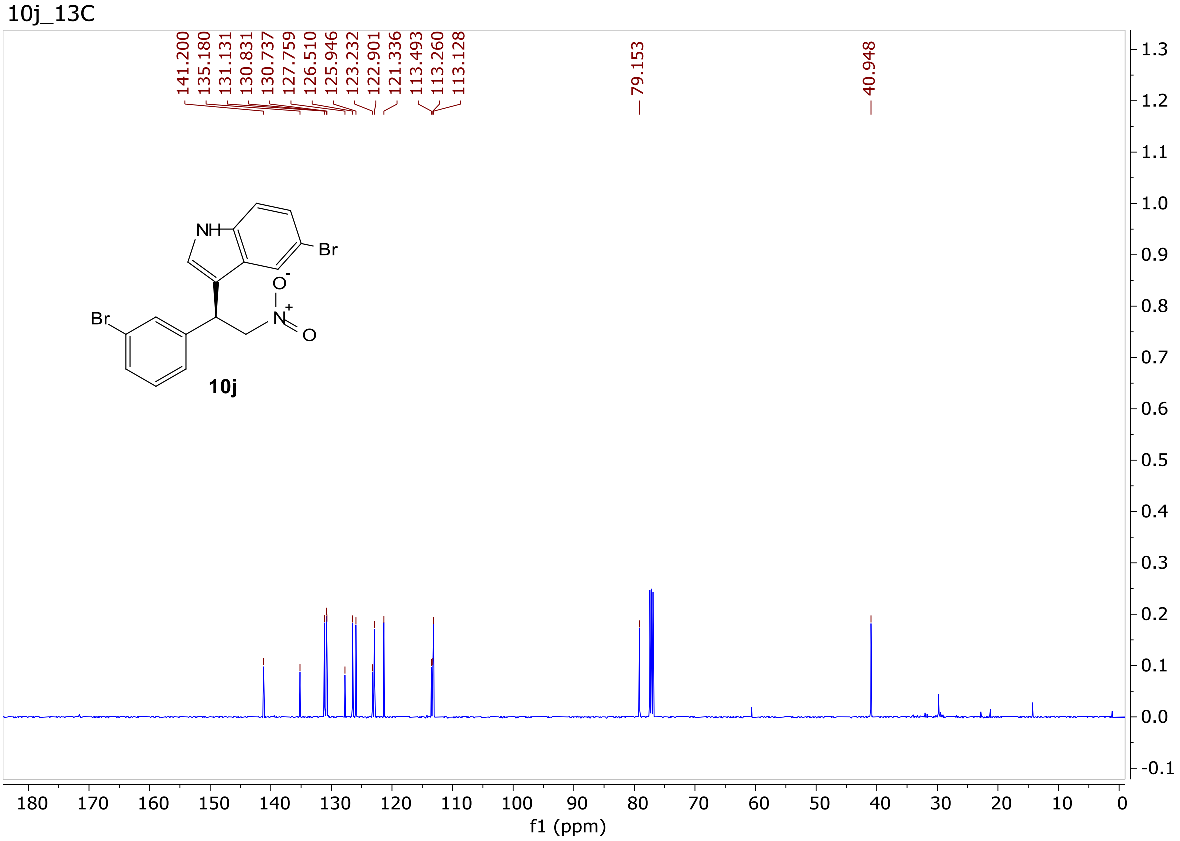

^{1}H NMR and ^{13}C-NMR for Friedel Craft product-10k

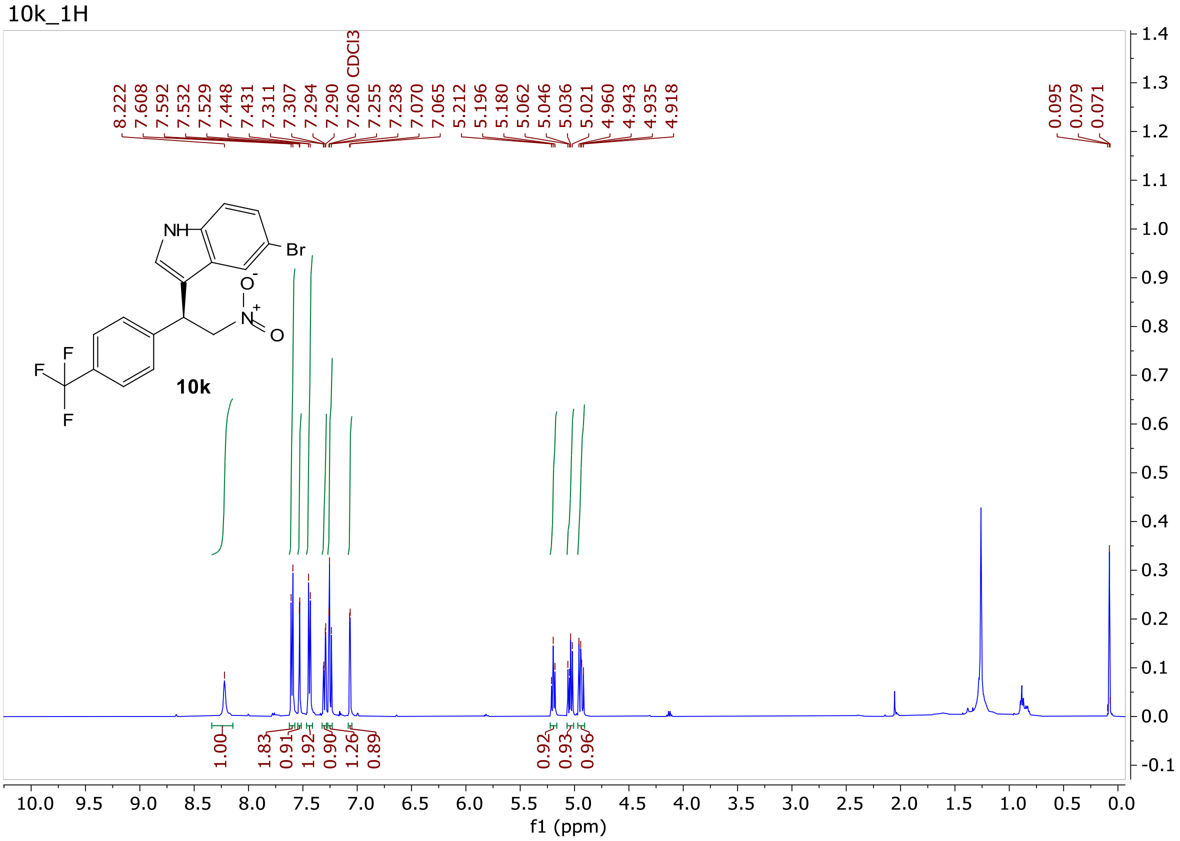

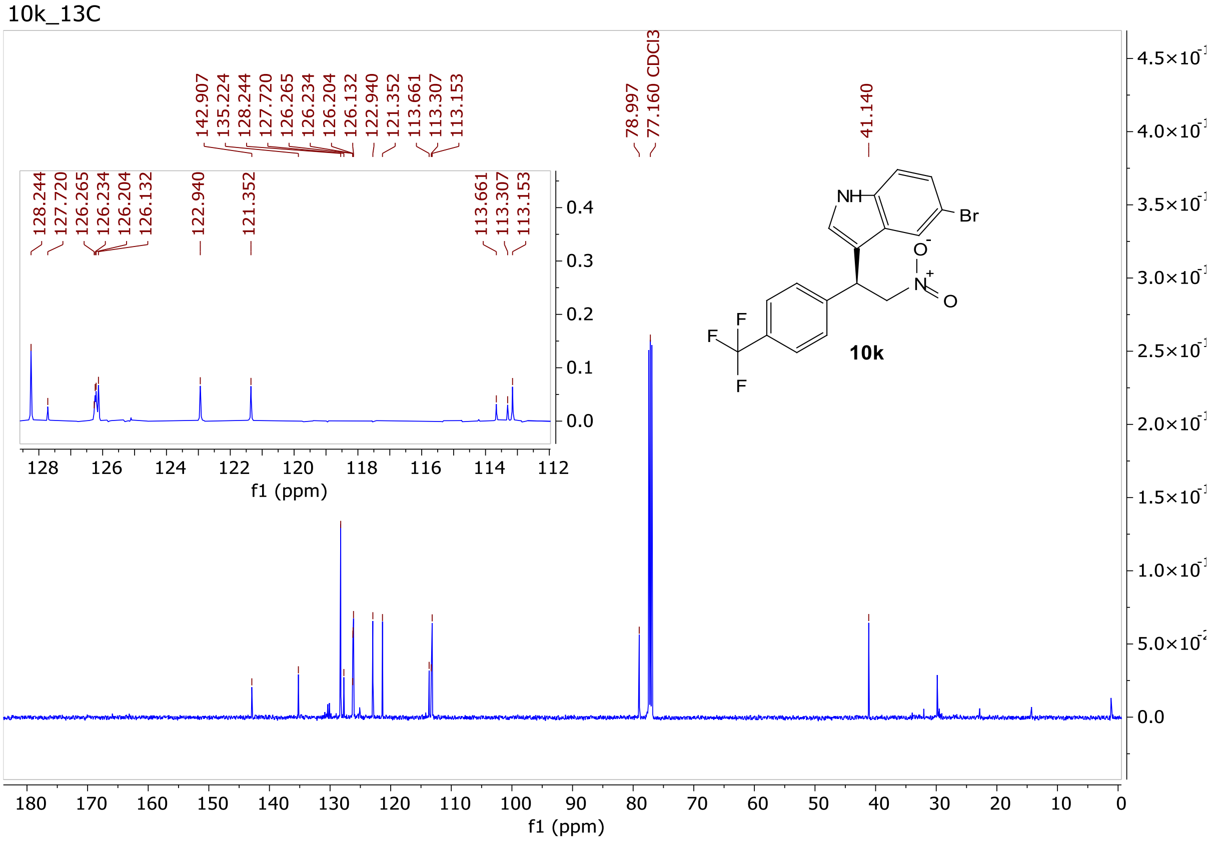

Electronic Supplementary file (ESI)

^{1}H NMR and ^{13}C-NMR for Friedel Craft product-10l

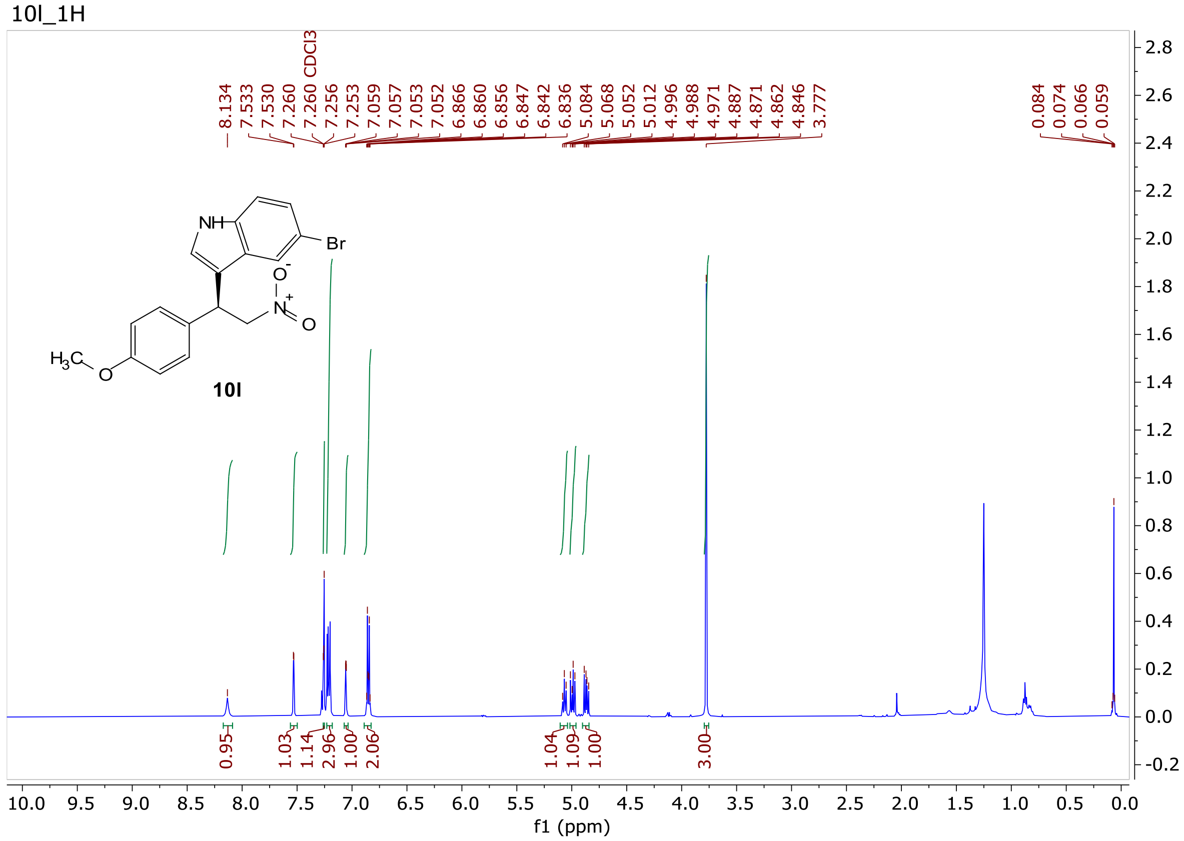

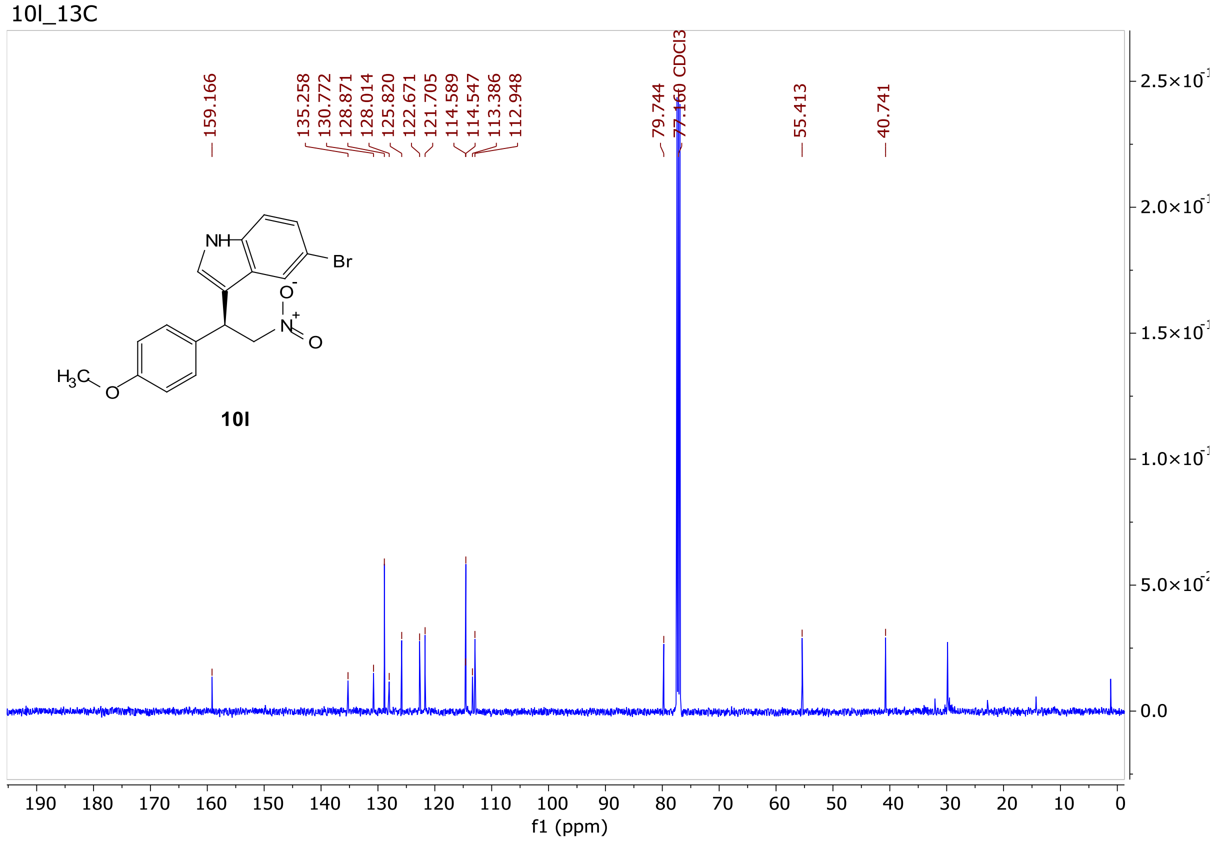

Electronic Supplementary file (ESI)

^{1}H NMR and ^{13}C-NMR for Friedel Craft product-10m

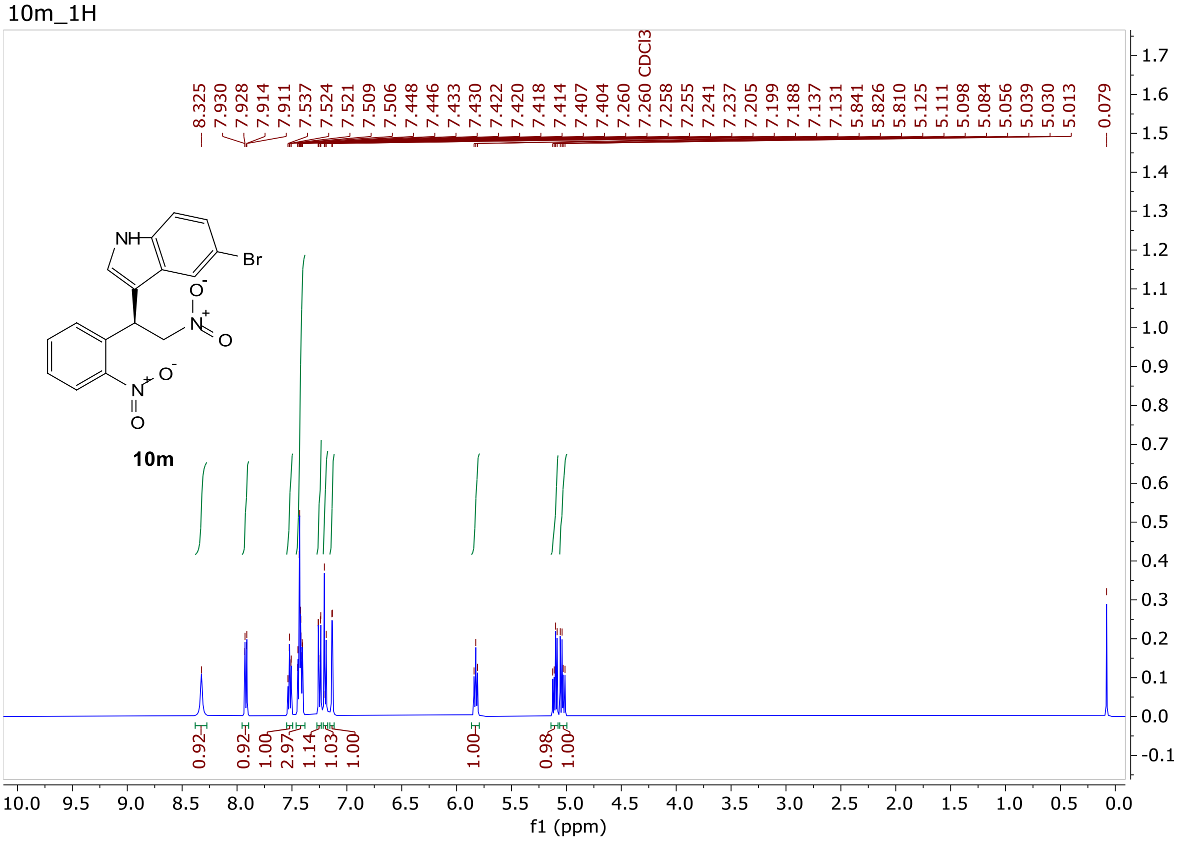

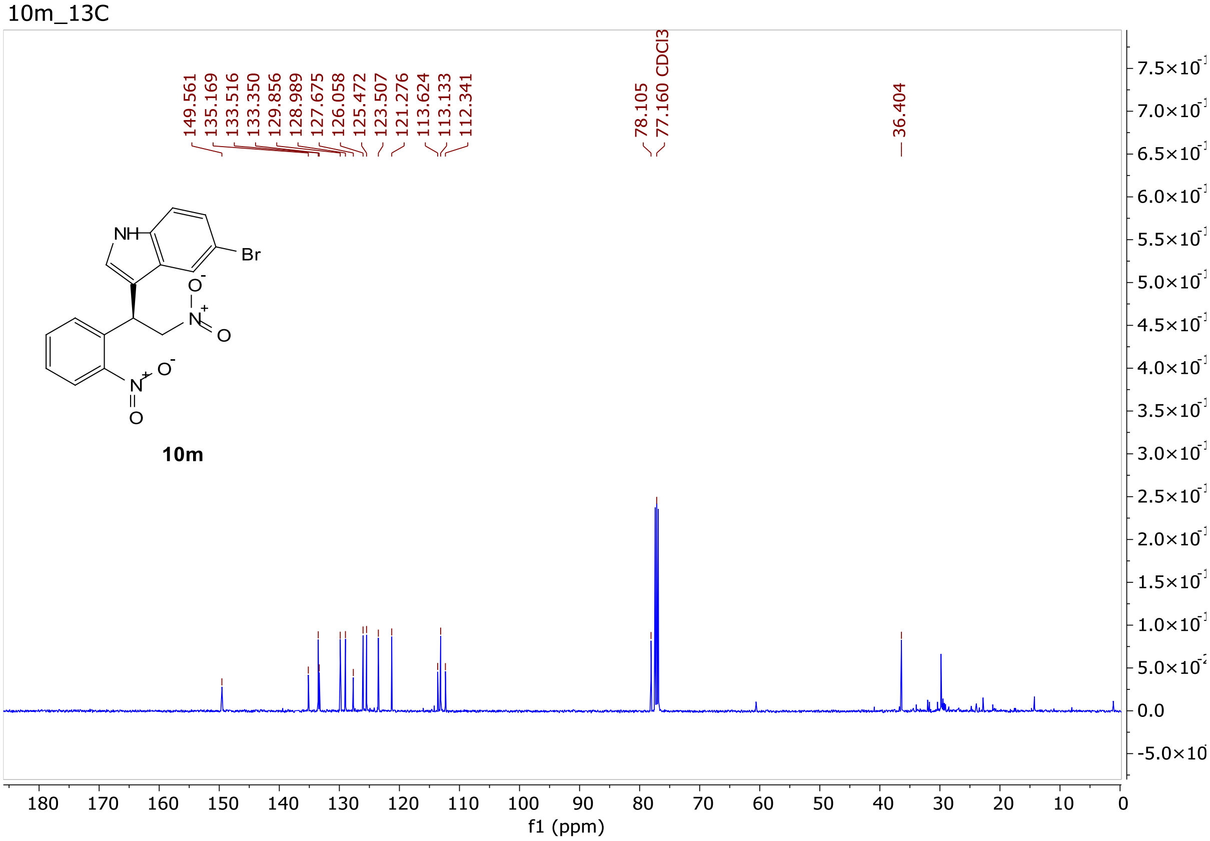

Electronic Supplementary file (ESI)

^{1}H NMR and ^{13}C-NMR for Friedel Craft product-10n

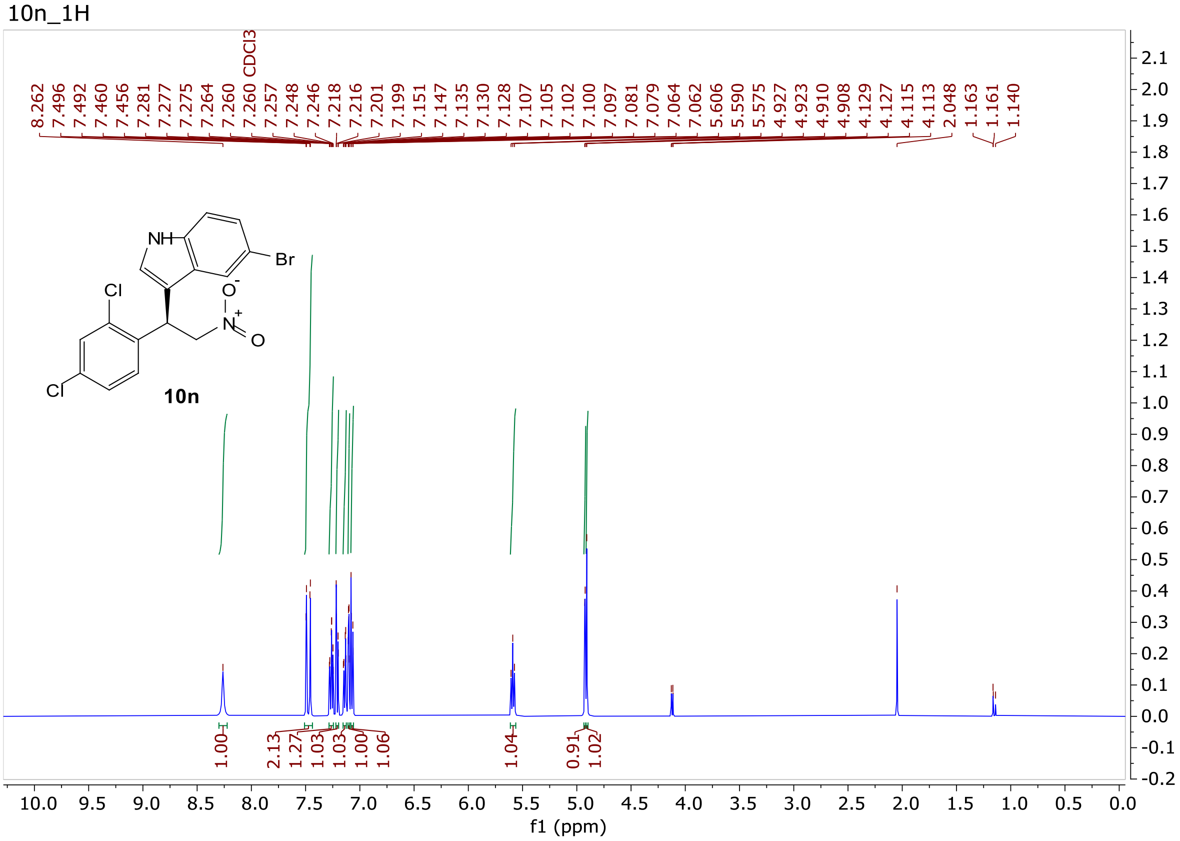

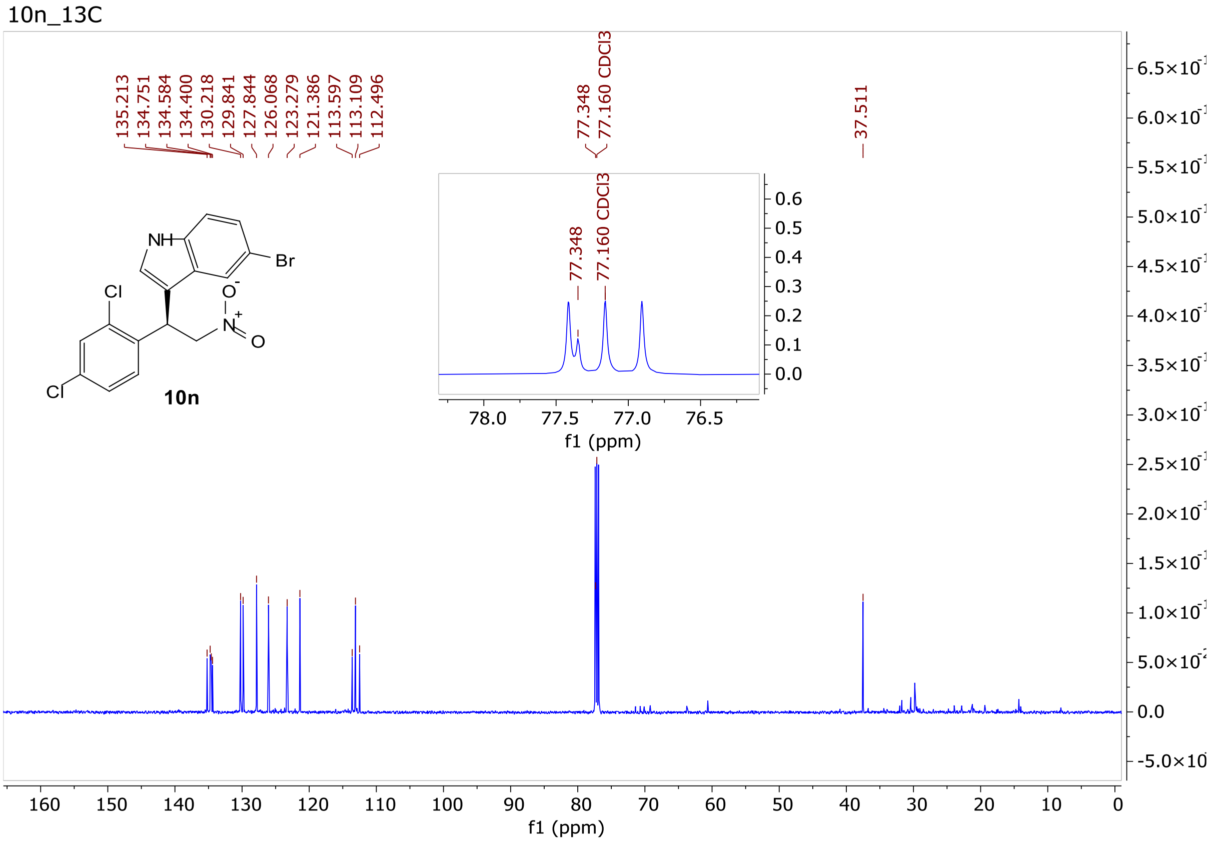

^{1}H NMR and ^{13}C-NMR for Friedel Craft product-10o

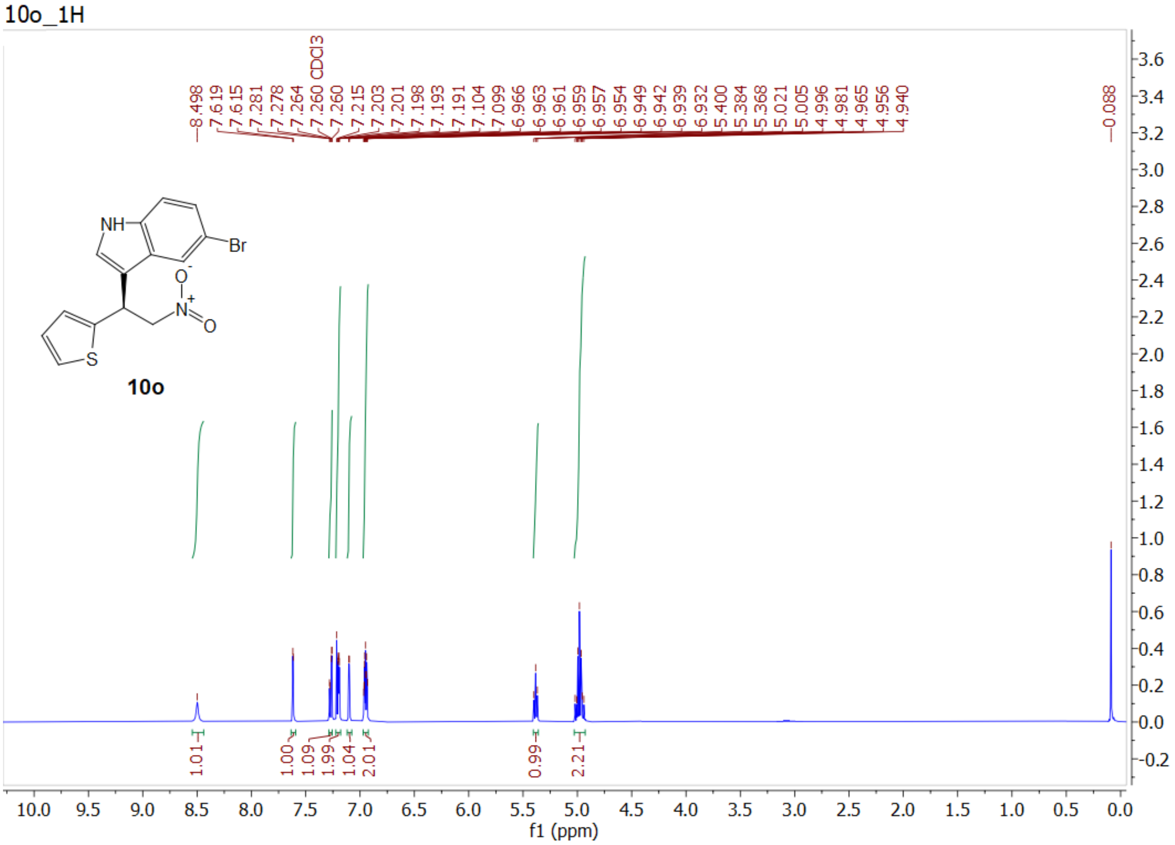

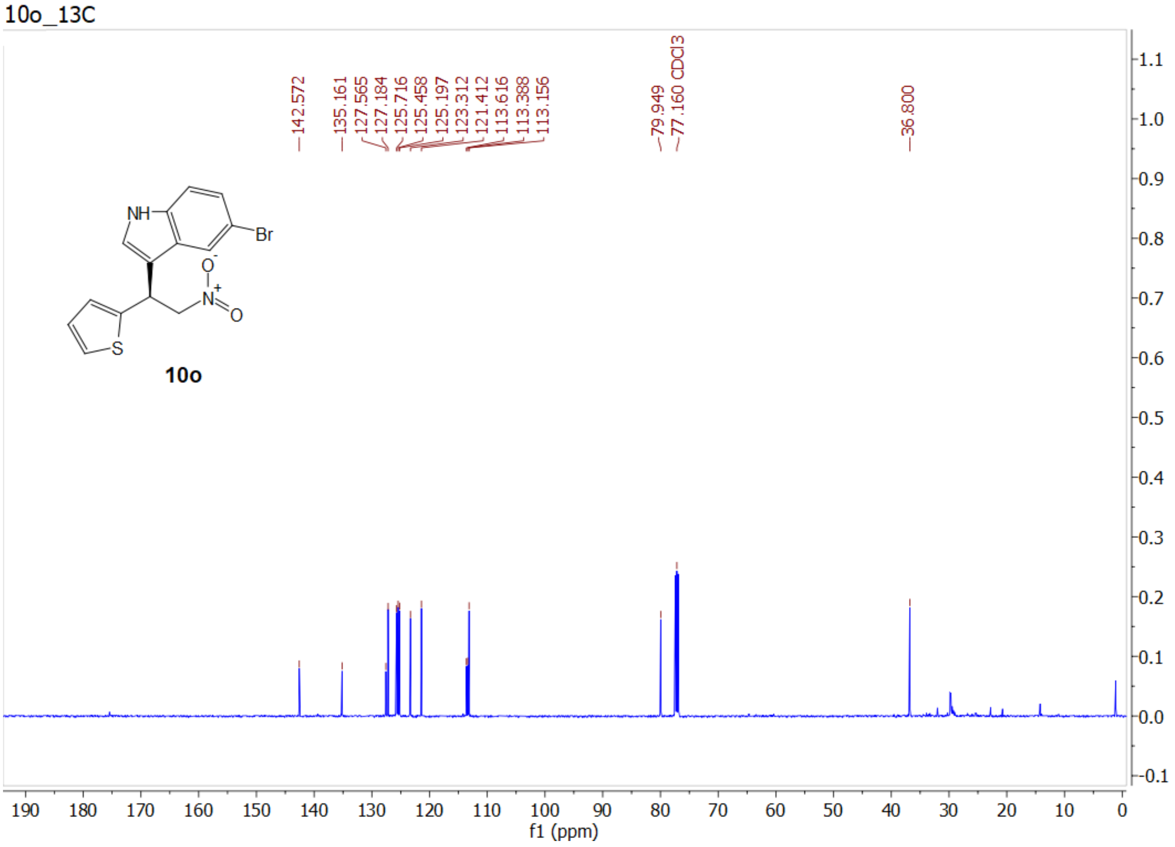

Electronic Supplementary file (ESI)

^{1}H NMR and ^{13}C-NMR for Friedel Craft product-10p

10p_1H

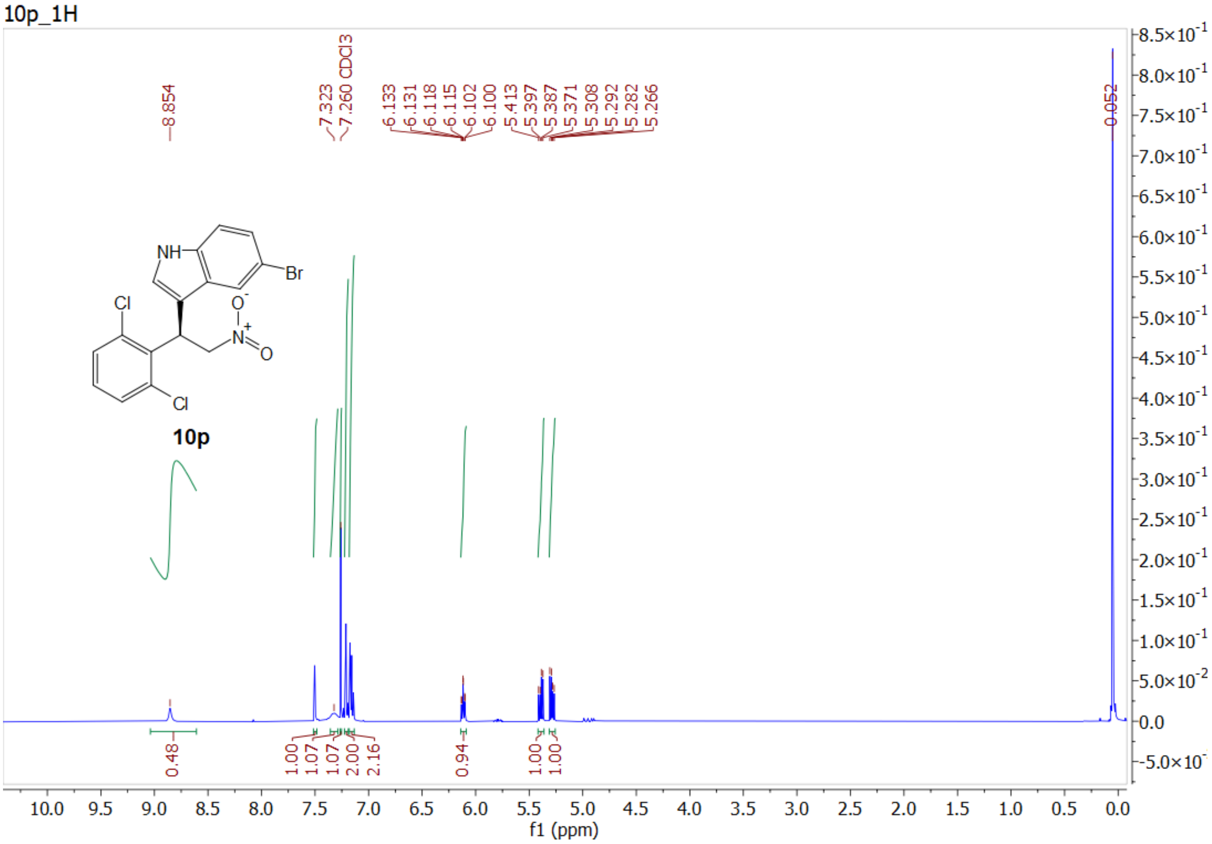

10p_13C

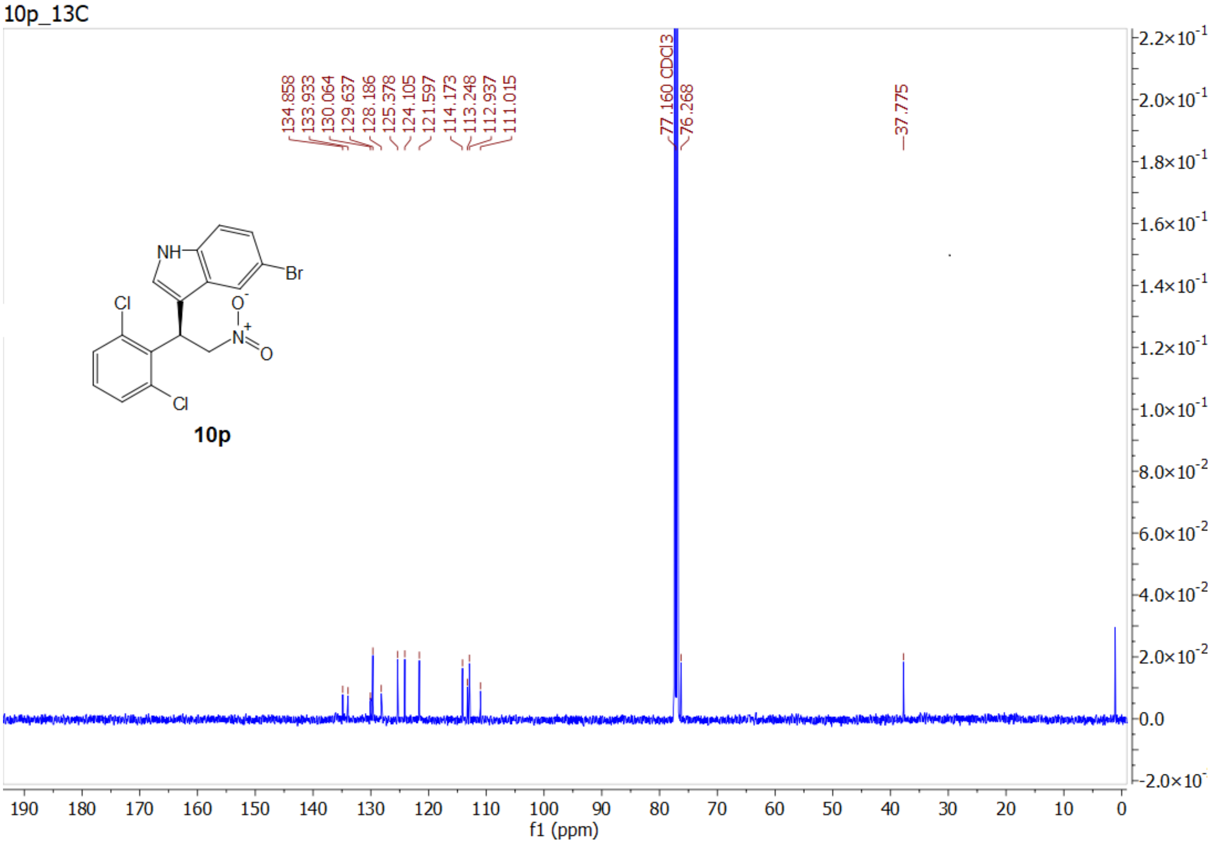

Electronic Supplementary file (ESI)

^{1}H NMR and ^{13}C-NMR for Friedel Craft product-10q

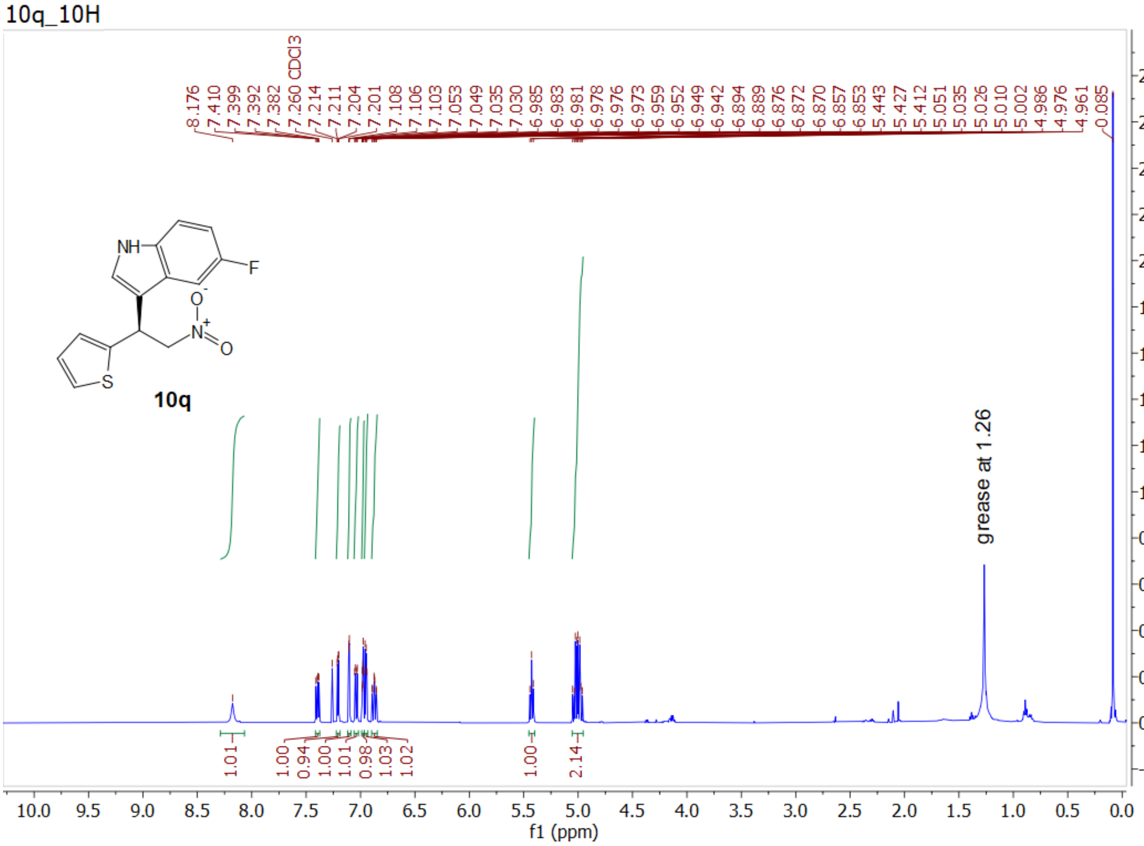

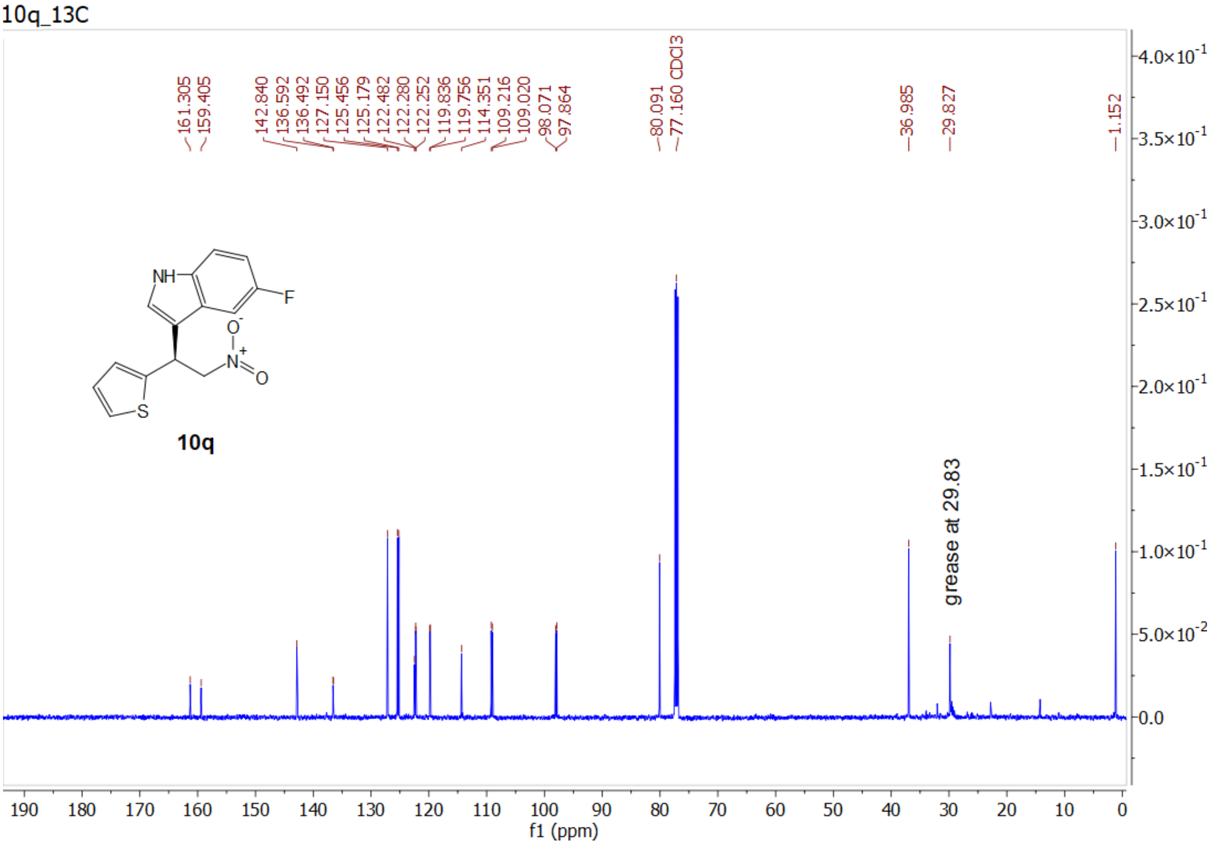

^{1}H NMR and ^{13}C-NMR for Friedel Craft product-10r

10r_1H

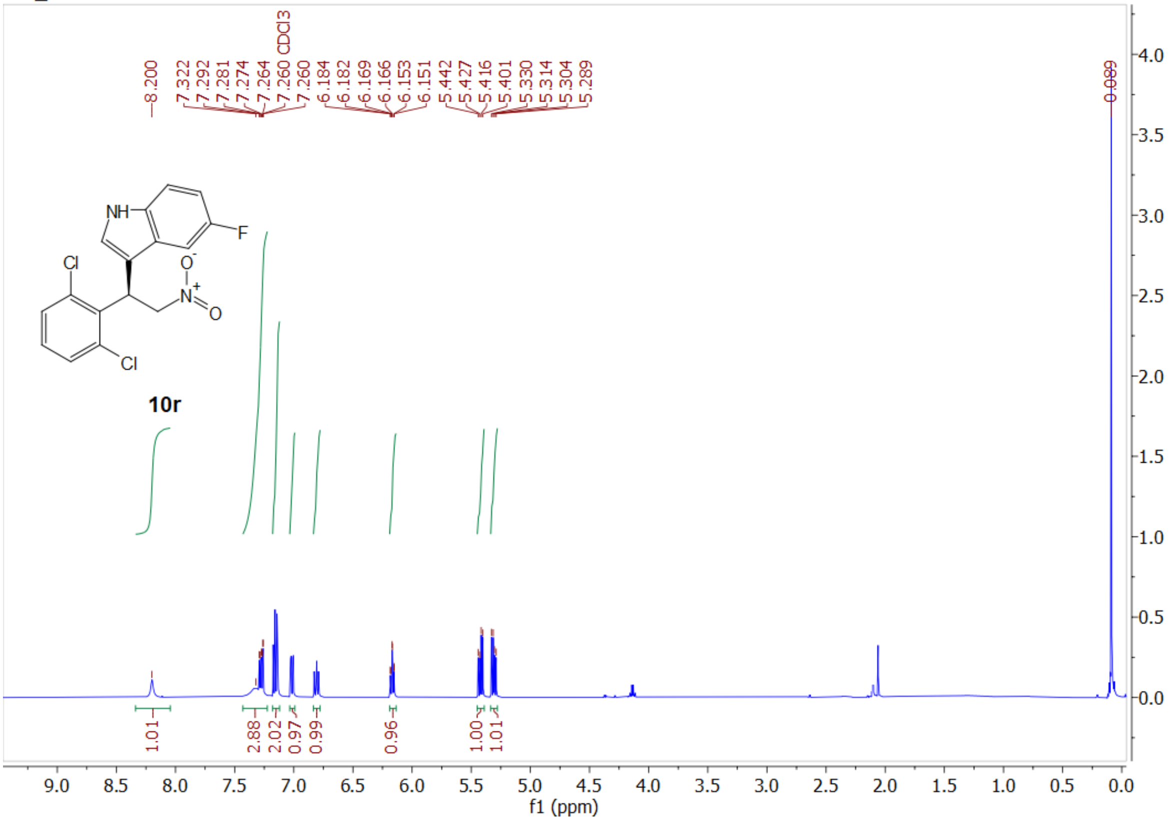

10r_13C

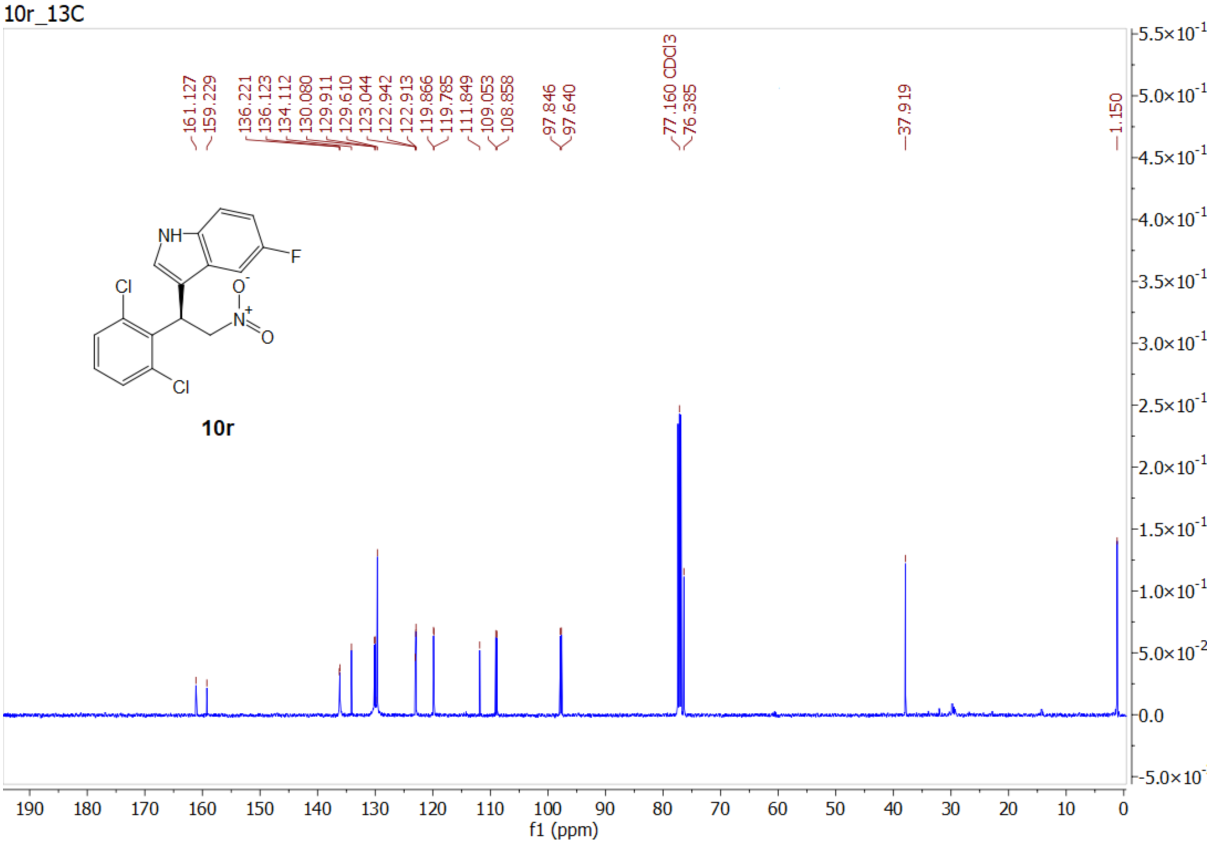

Electronic Supplementary file (ESI)

^{1}H NMR and ^{13}C-NMR for Friedel Craft product-10s

10s_1H

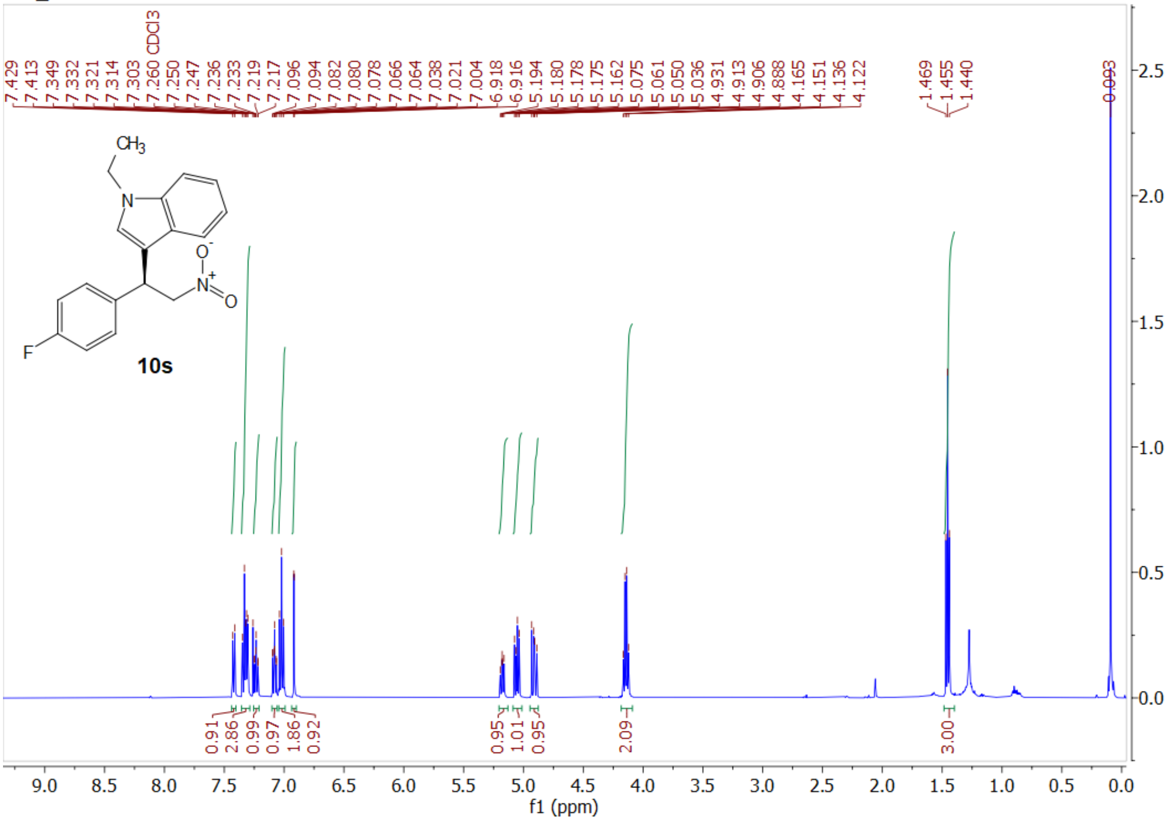

10s_13C

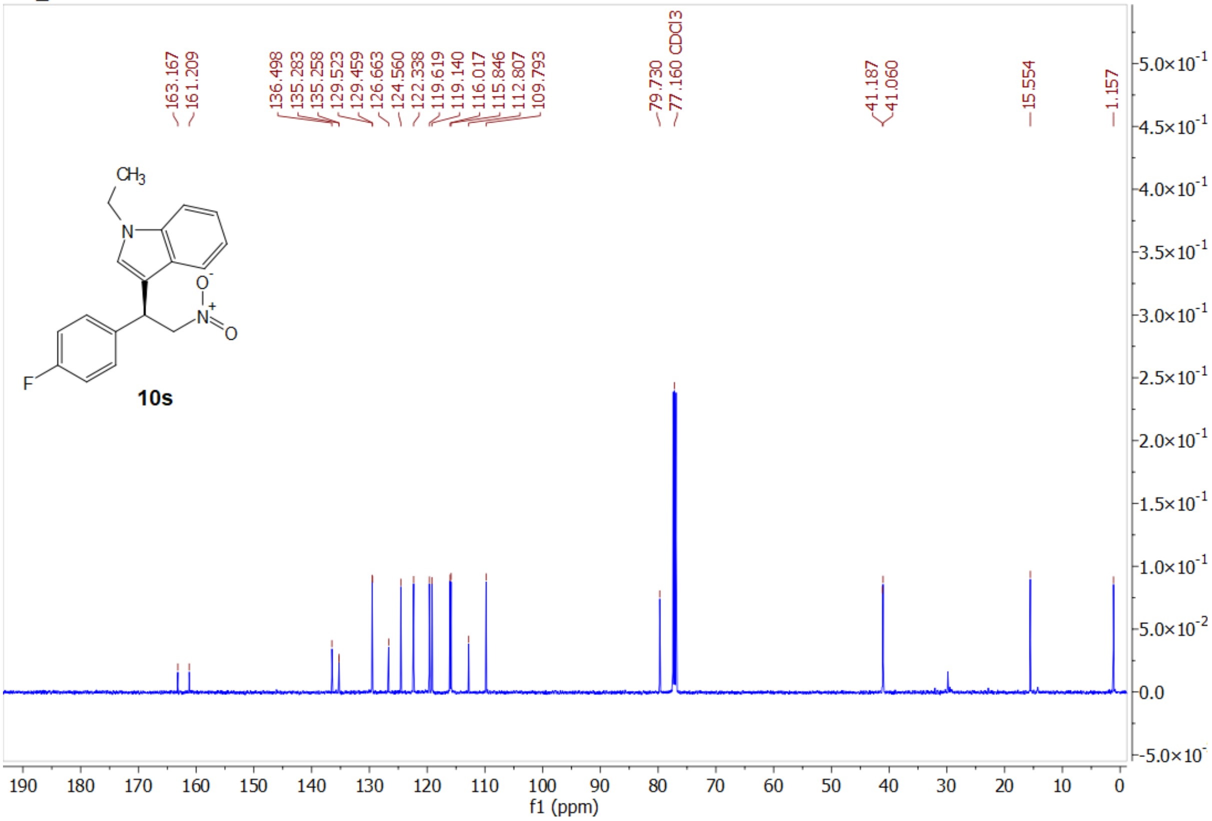

^{1}H NMR and ^{13}C-NMR for Friedel Craft product-10t

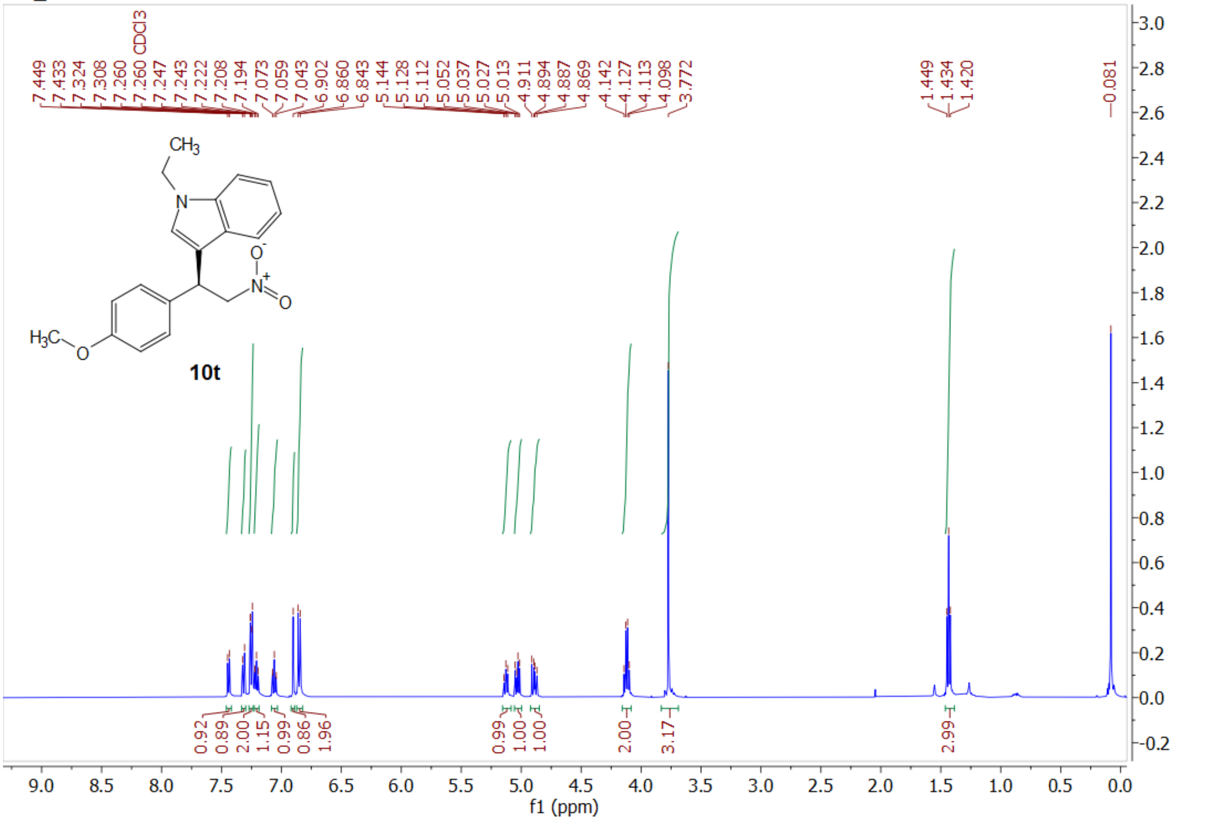

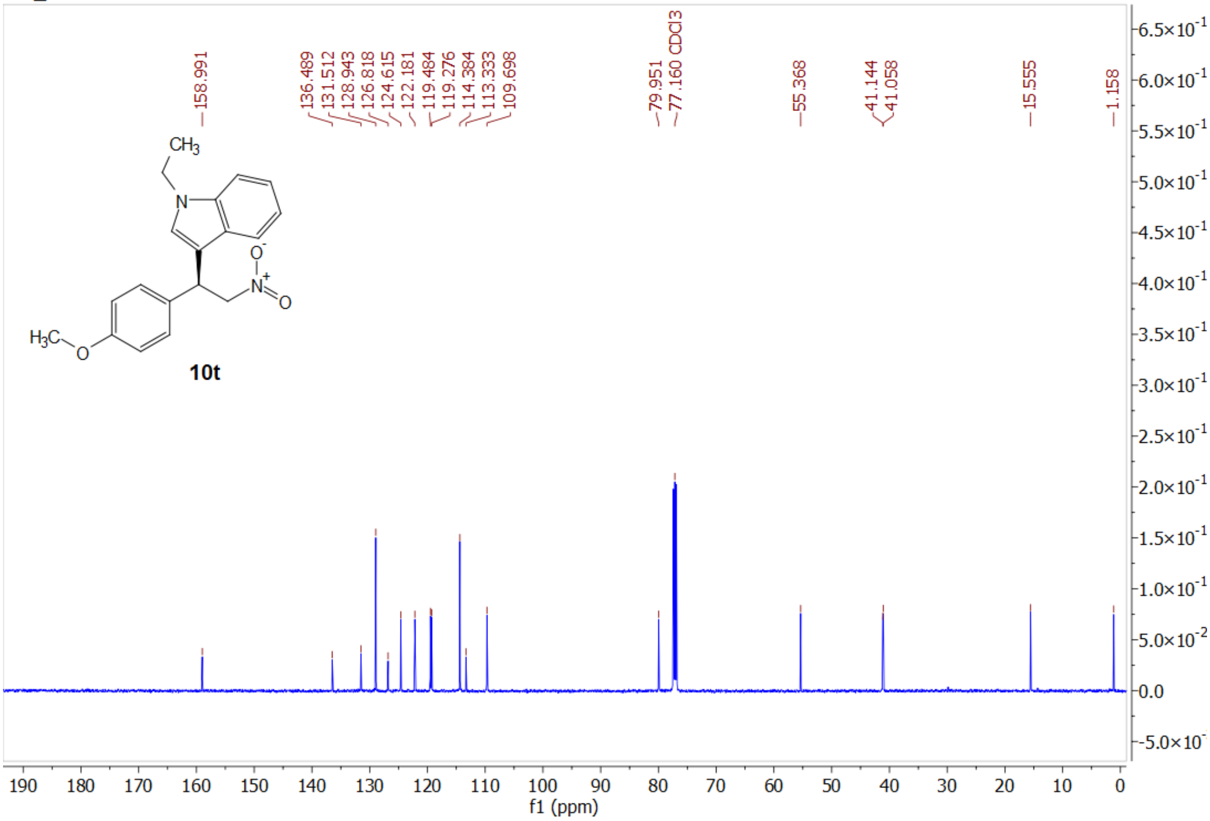

3. Chiral HPLC Analysis

Chiral HPLC purity of ligand L1

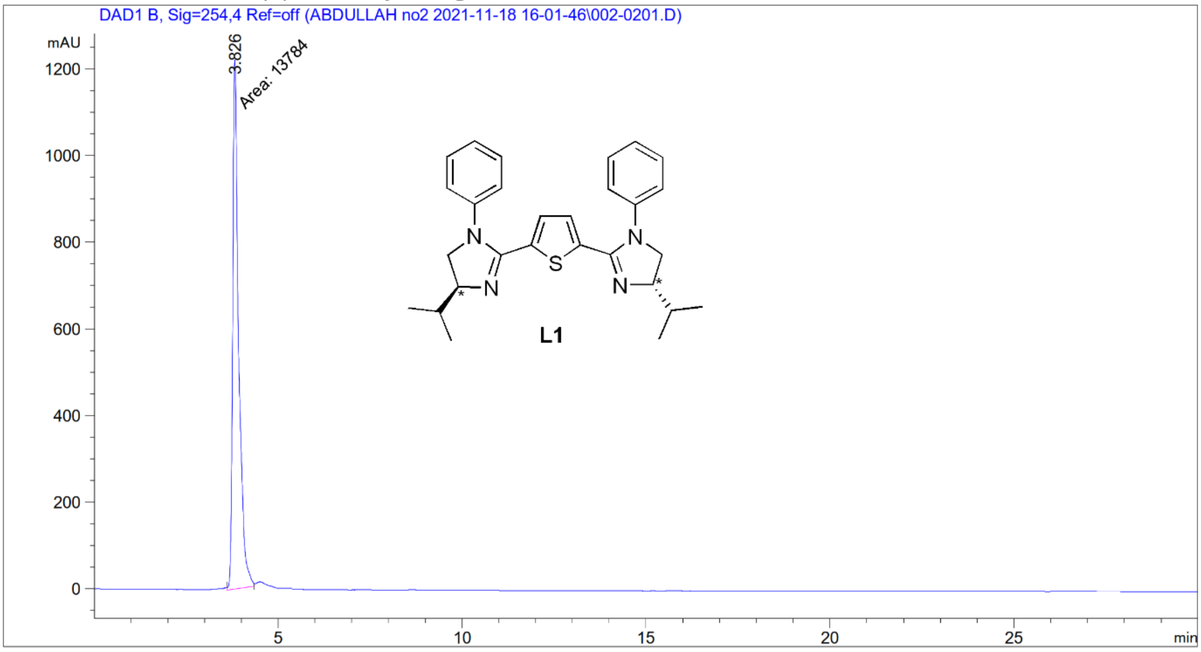

Peak #	RetTime [min]	Type	Width [min]	Area [mAU*s]	Height [mAU]	Area %
1	3.826	MM	0.1880	1.37840e4	1222.21545	100.0000

Chiral HPLC purity of ligand L2

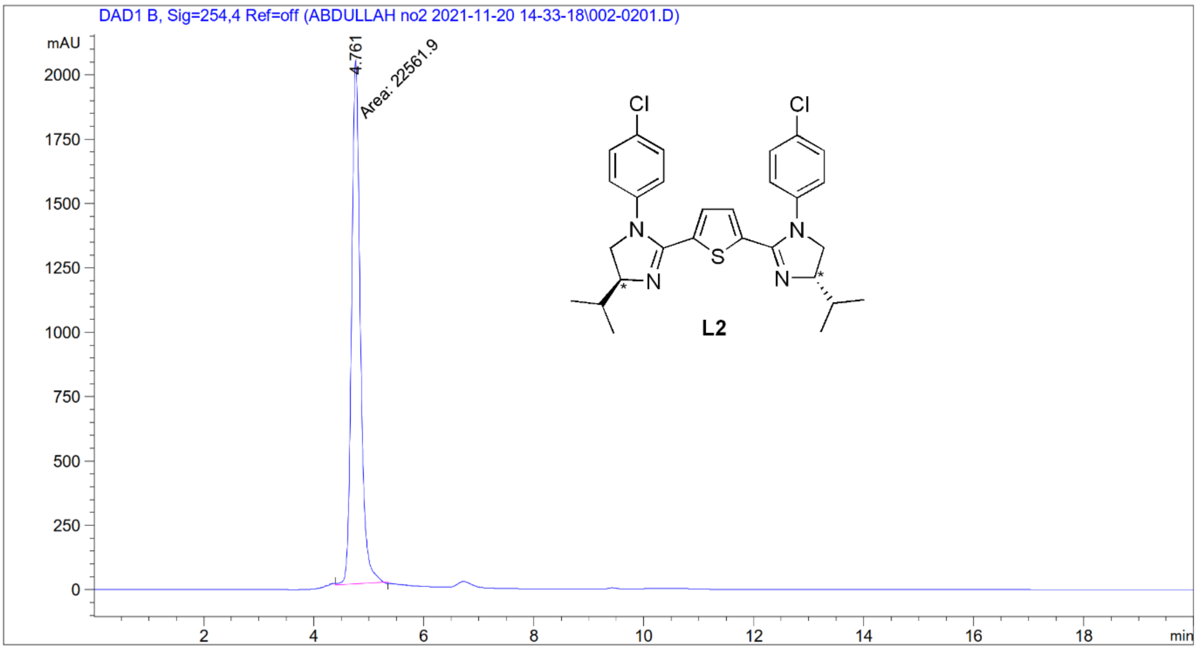

Peak #	RetTime [min]	Type	Width [min]	Area [mAU*s]	Height [mAU]	Area %
1	4.761	FM	0.1848	2.25619e4	2035.34668	100.0000

Chiral HPLC purity of ligand L3

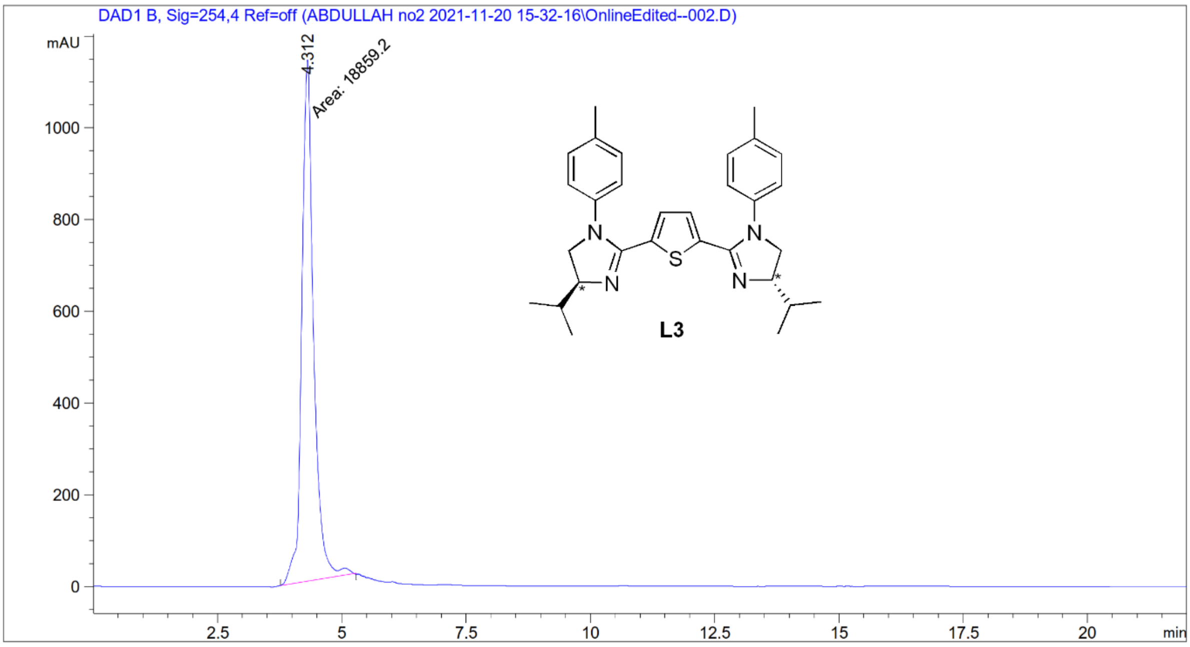

Peak #	RetTime [min]	Type	Width [min]	Area [mAU*s]	Height [mAU]	Area %
1	4.312	MM	0.2764	1.88592e4	1137.12976	100.0000

Chiral HPLC purity of ligand L4

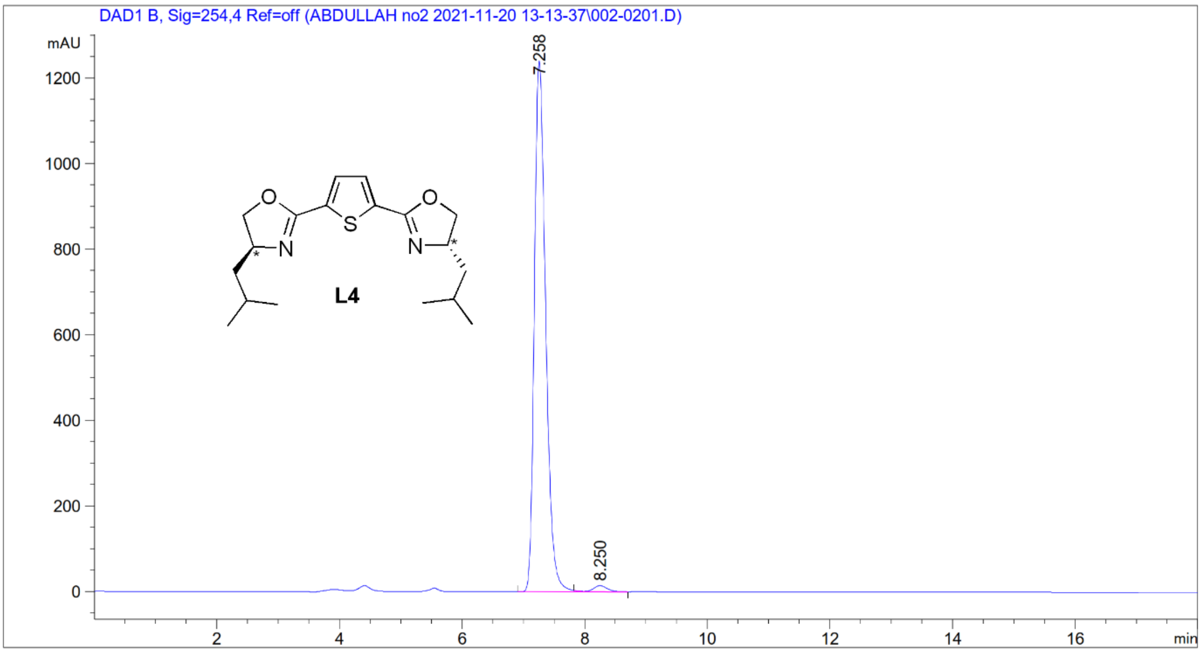

Peak #	RetTime [min]	Type	Width [min]	Area [mAU*s]	Height [mAU]	Area %
1	7.258	BV R	0.1952	1.58857e4	1241.89453	98.6009
2	8.250	VB E	0.2389	225.40587	14.37647	1.3991

Chiral HPLC purity of ligand L5

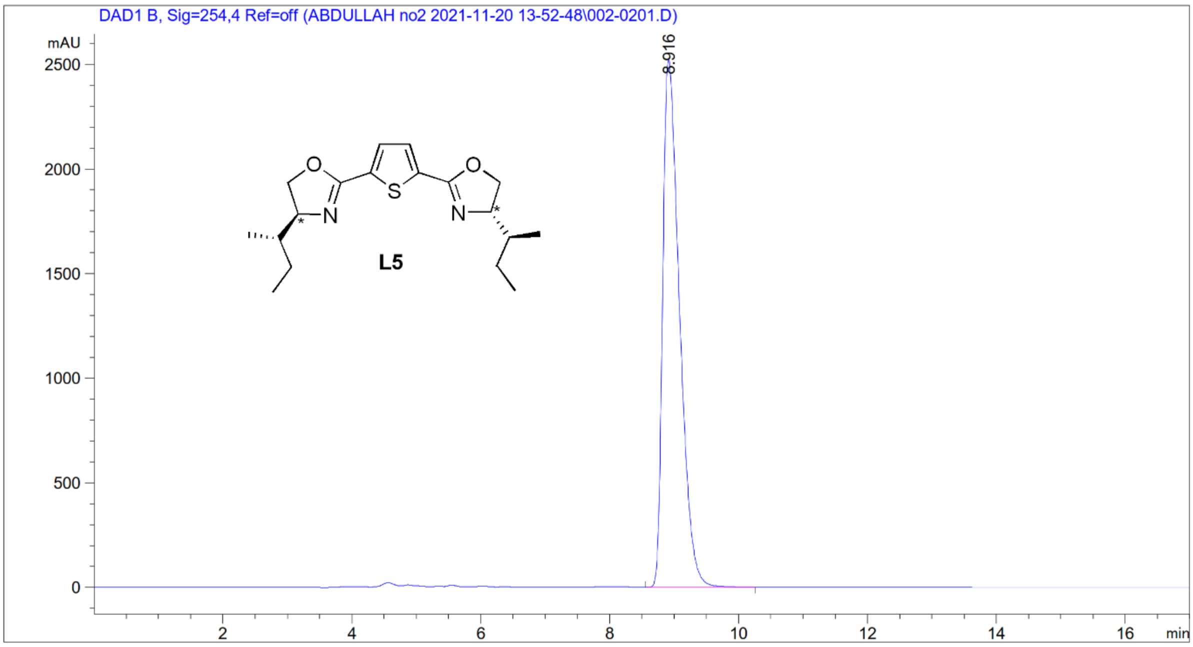

Peak #	RetTime [min]	Type	Width [min]	Area [mAU*s]	Height [mAU]	Area %
1	8.916	BB	0.2771	4.56122e4	2520.79419	100.0000

Chiral HPLC for Friedel Craft product-10a

Racemic-10a

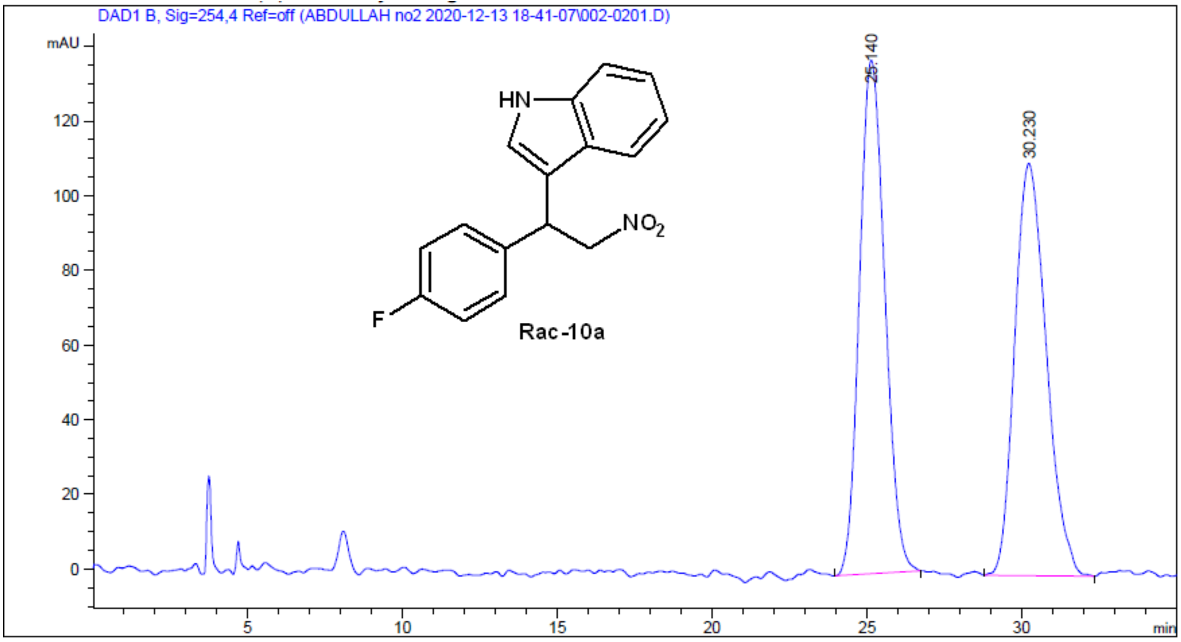

Peak #	RetTime [min]	Type	Width [min]	Area [mAU*s]	Height [mAU]	Area %
1	25.140	BB	0.8828	7832.65381	137.45441	49.9784
2	30.230	BB	1.0606	7839.41602	110.38467	50.0216

Chiral-10a

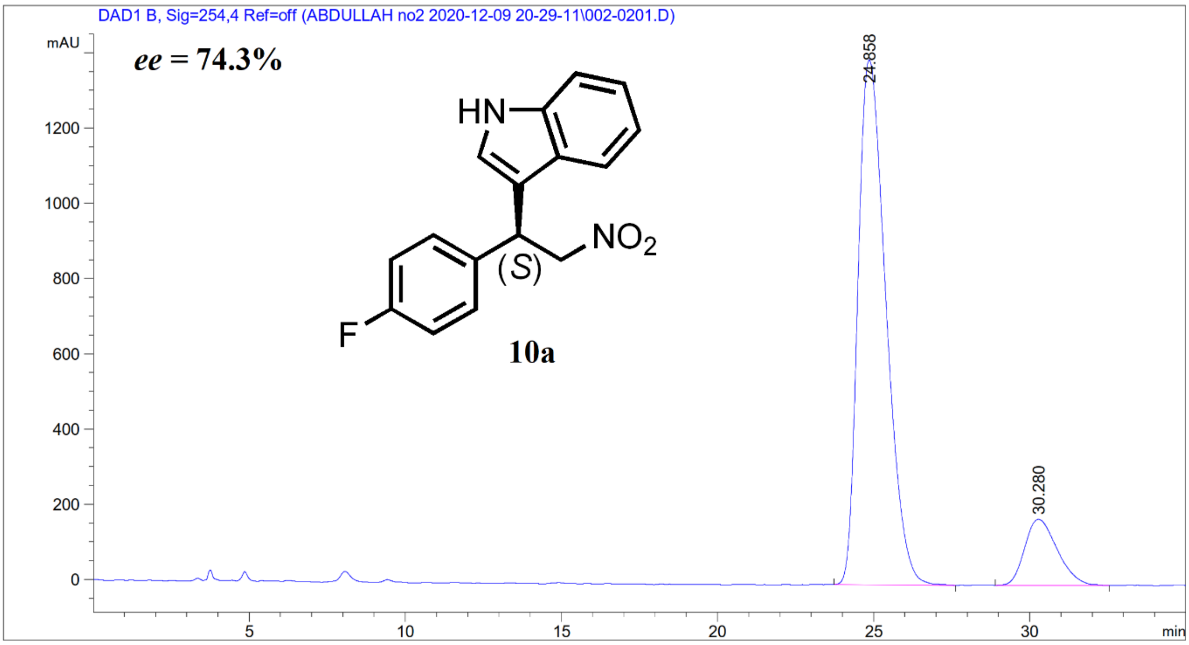

Peak #	RetTime [min]	Type	Width [min]	Area [mAU*s]	Height [mAU]	Area %
1	24.858	BB	0.9623	8.65939e4	1394.88562	87.1544
2	30.280	BB	1.1069	1.27629e4	175.39699	12.8456

Chiral-10a_large scale

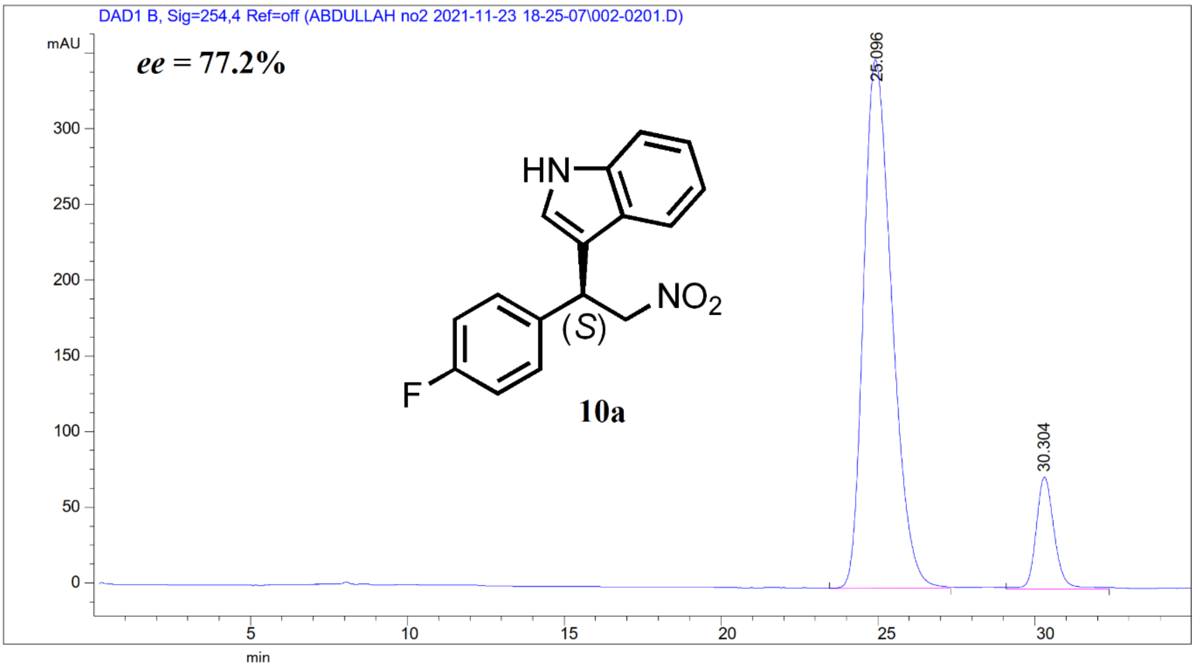

Peak #	RetTime [min]	Type	Width [min]	Area [mAU*s]	Height [mAU]	Area %
1	25.096	BB	0.6949	1.49555e4	348.72155	88.5985
2	30.304	BB	0.4260	1.92452e3	69.94374	11.4015

Chiral HPLC for Friedel Craft product-10b

Racemic-10b

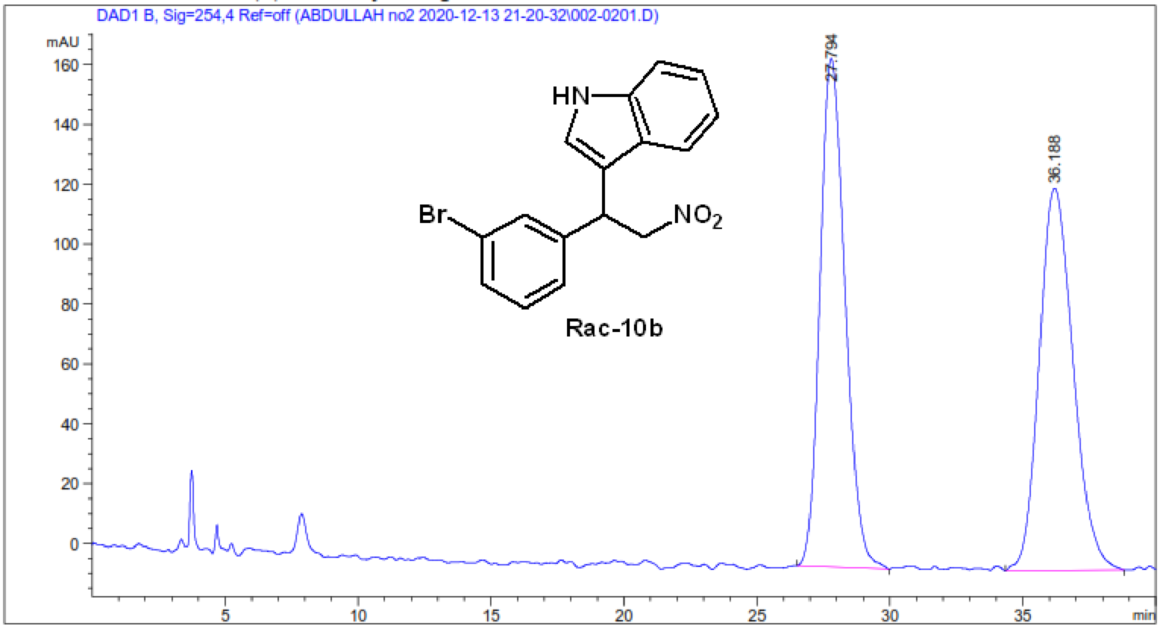

Peak #	RetTime [min]	Type	Width [min]	Area [mAU*s]	Height [mAU]	Area %
1	27.794	BB	1.0067	1.11889e4	169.81950	49.6943
2	36.188	BB	1.2488	1.13266e4	127.70029	50.3057

Chiral-10b

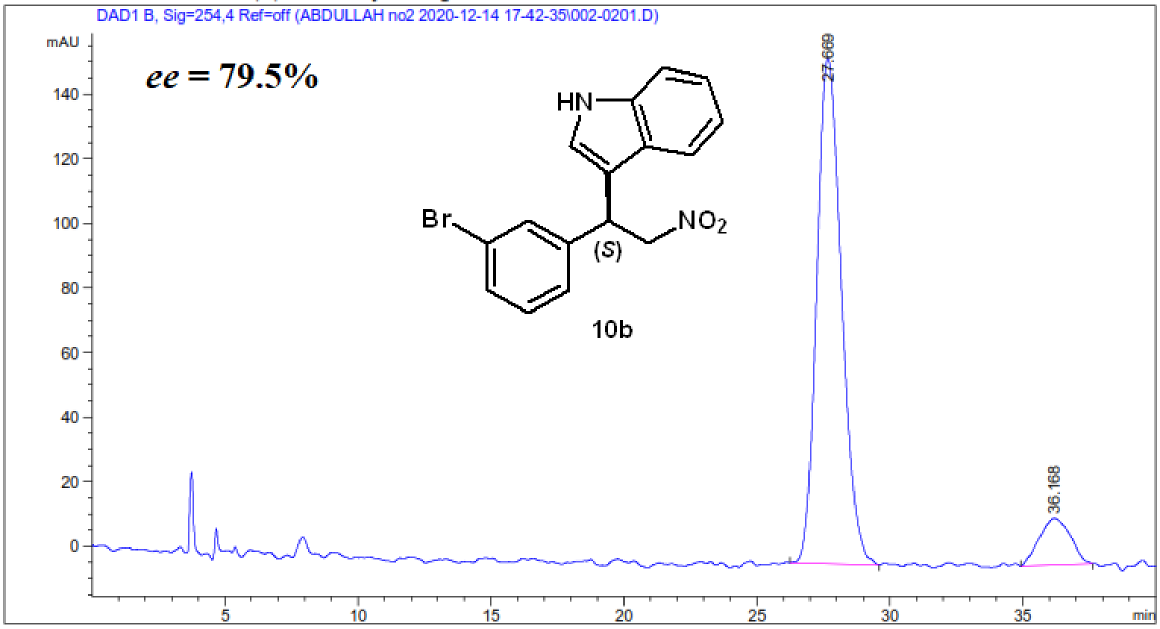

Peak #	RetTime [min]	Type	Width [min]	Area [mAU*s]	Height [mAU]	Area %
1	27.669	BB	1.0038	1.01604e4	156.42024	89.7367
2	36.168	BB	0.9597	1162.05957	14.42719	10.2633

Chiral HPLC for Friedel Craft product-10c

Racemic-10c

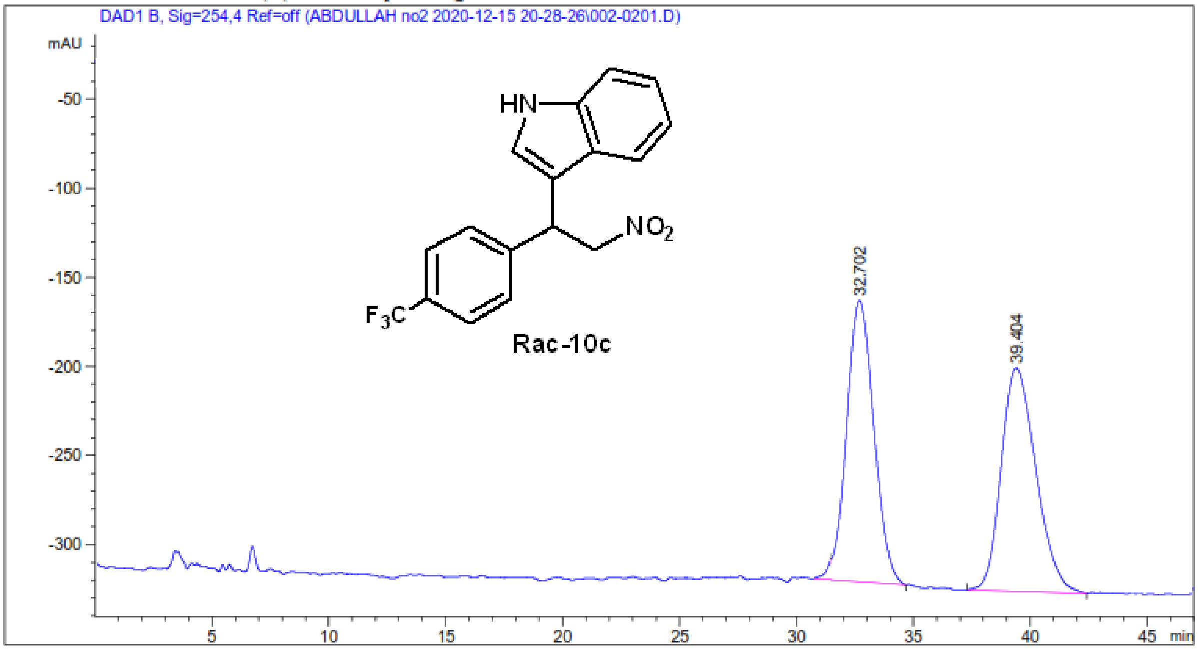

Peak #	RetTime [min]	Type	Width [min]	Area [mAU*s]	Height [mAU]	Area %
1	32.702	VB	1.2346	1.27554e4	157.88155	49.6814
2	39.404	BB	1.4974	1.29190e4	125.24584	50.3186

Chiral-10c

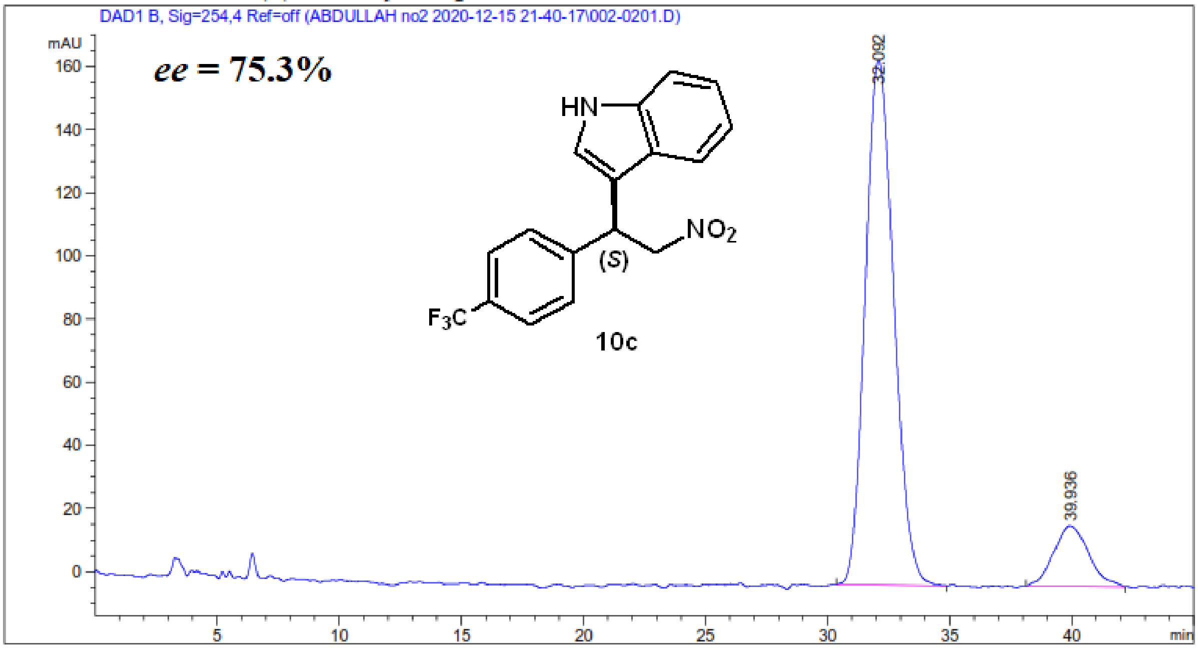

Peak #	RetTime [min]	Type	Width [min]	Area [mAU*s]	Height [mAU]	Area %
1	32.092	BB	1.2608	1.34778e4	166.06856	87.6837
2	39.936	BB	1.1672	1893.11694	19.08630	12.3163

Chiral HPLC for Friedel Craft product-10d

Racemic-10d

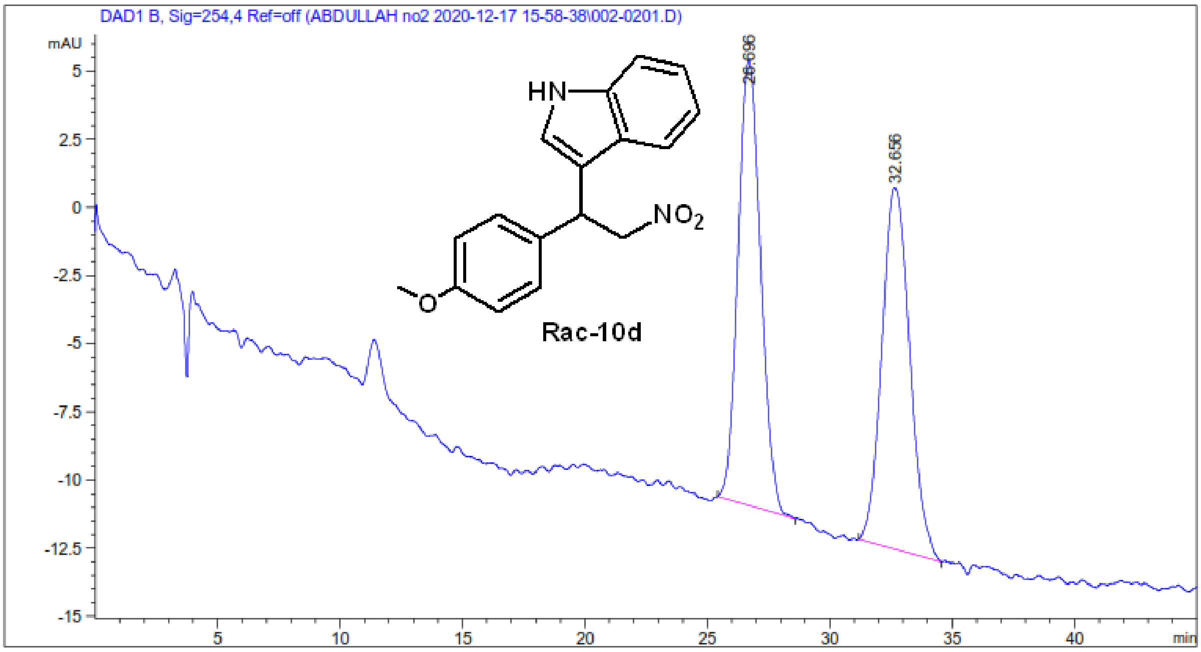

Peak #	RetTime [min]	Type	Width [min]	Area [mAU*s]	Height [mAU]	Area %
1	26.696	BB	0.8136	1048.73560	16.32175	49.9485
2	32.656	BB	0.9430	1050.89722	13.25501	50.0515

Chiral-10d

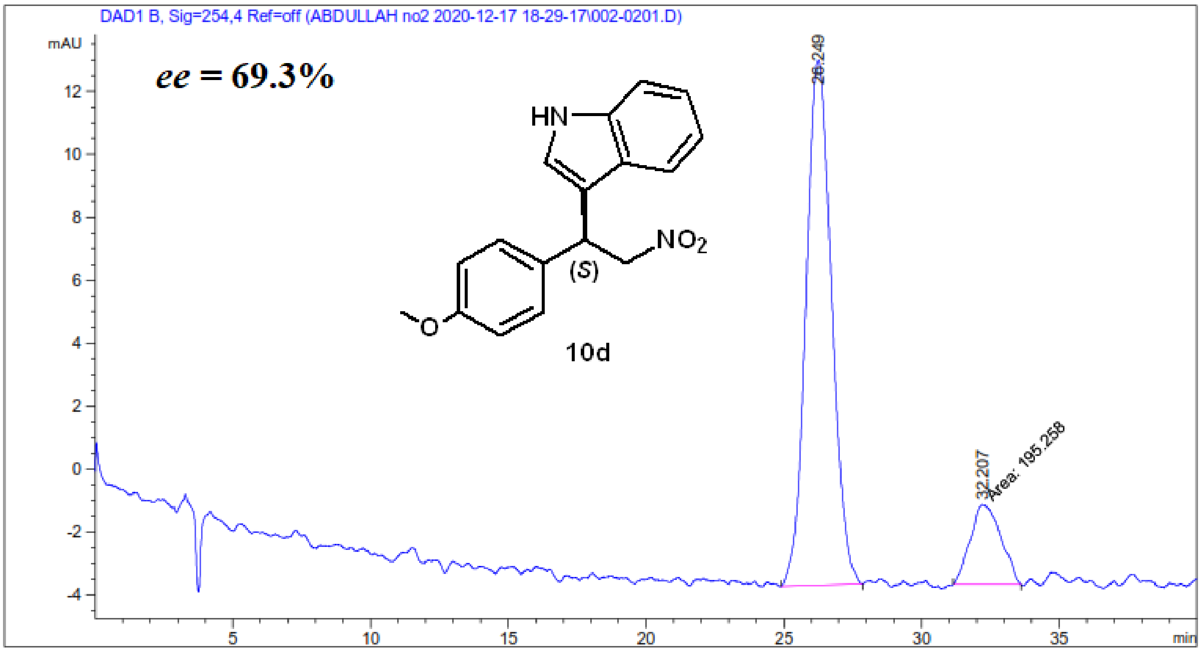

Peak #	RetTime [min]	Type	Width [min]	Area [mAU*s]	Height [mAU]	Area %
1	26.249	BB	0.8712	1076.49341	16.67473	84.6465
2	32.207	MM	1.2825	195.25804	2.53749	15.3535

Chiral HPLC for Friedel Craft product-10e

Racemic-10e

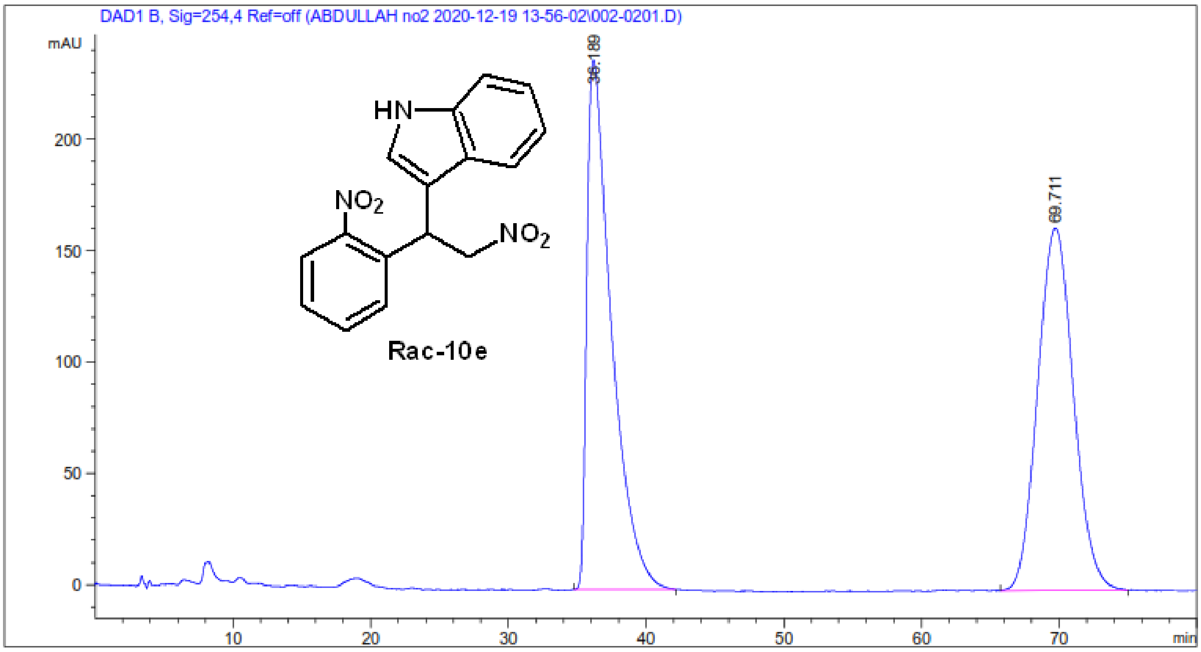

Peak #	RetTime [min]	Type	Width [min]	Area [mAU*s]	Height [mAU]	Area %
1	36.189	BB	1.7324	2.94402e4	237.45752	50.0721
2	69.711	BB	2.3206	2.93554e4	162.57515	49.9279

Chiral-10e

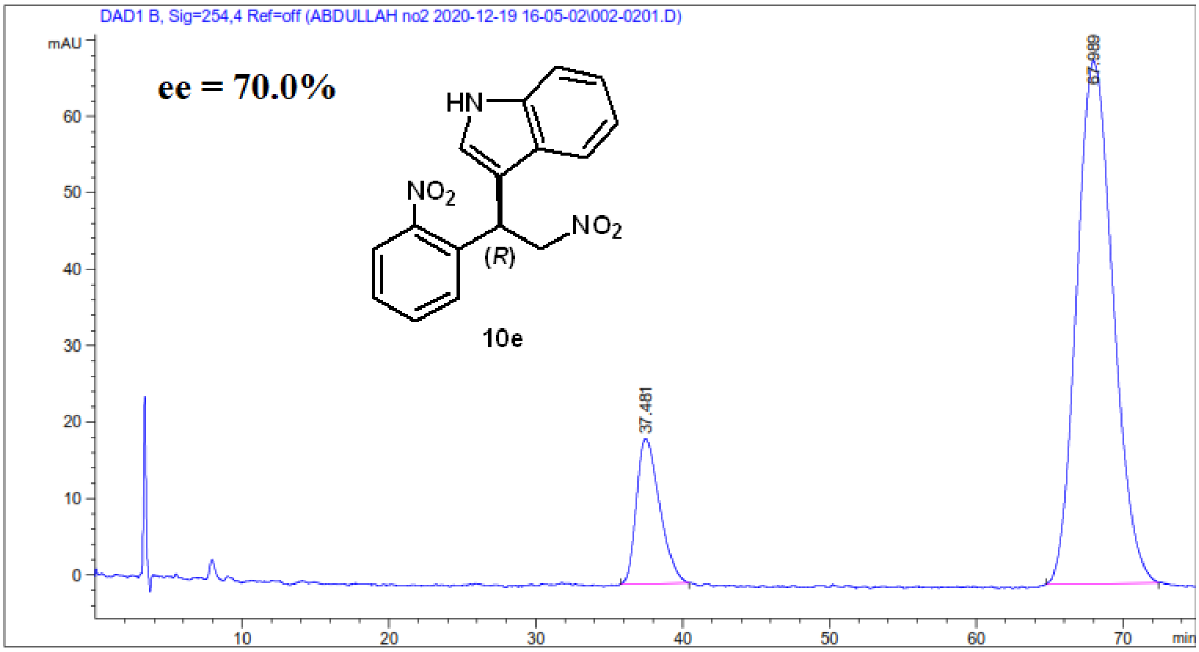

Peak #	RetTime [min]	Type	Width [min]	Area [mAU*s]	Height [mAU]	Area %
1	37.481	BB	1.2879	2084.03345	18.96646	15.0086
2	67.989	BB	2.0295	1.18016e4	68.44755	84.9914

Chiral HPLC for Friedel Craft product-10f

Racemic-10f

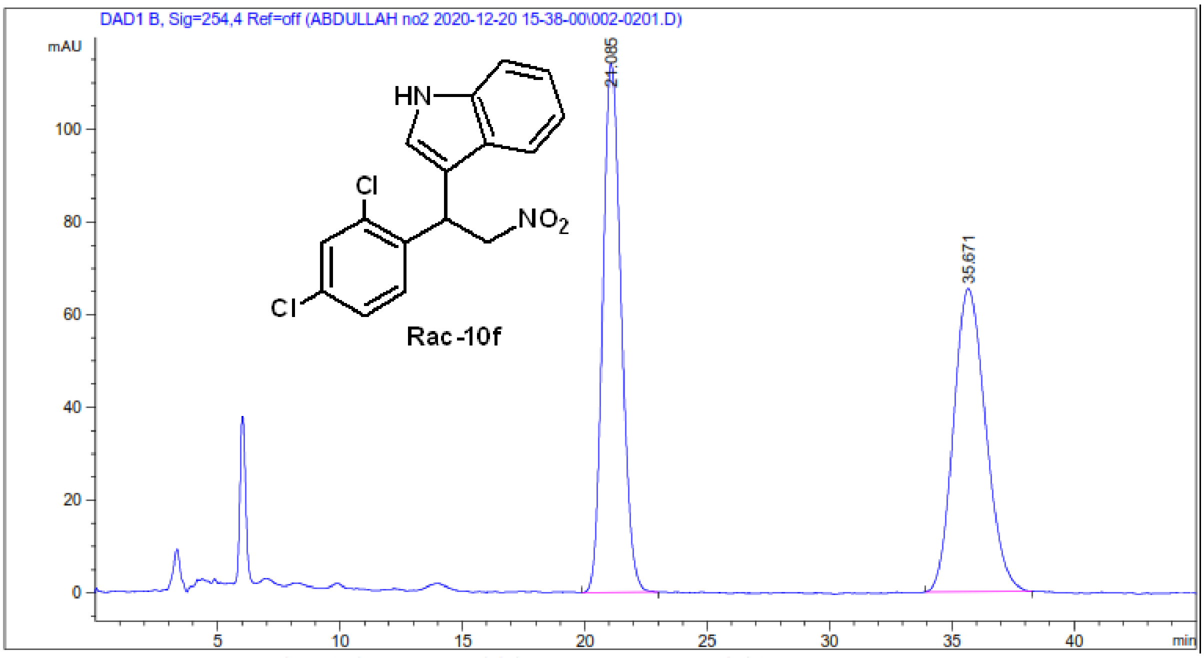

Peak #	RetTime [min]	Type	Width [min]	Area [mAU*s]	Height [mAU]	Area %
1	21.085	BB	0.8150	5972.73193	114.04918	50.0005
2	35.671	BB	1.2496	5972.62061	65.39095	49.9995

Chiral-10f

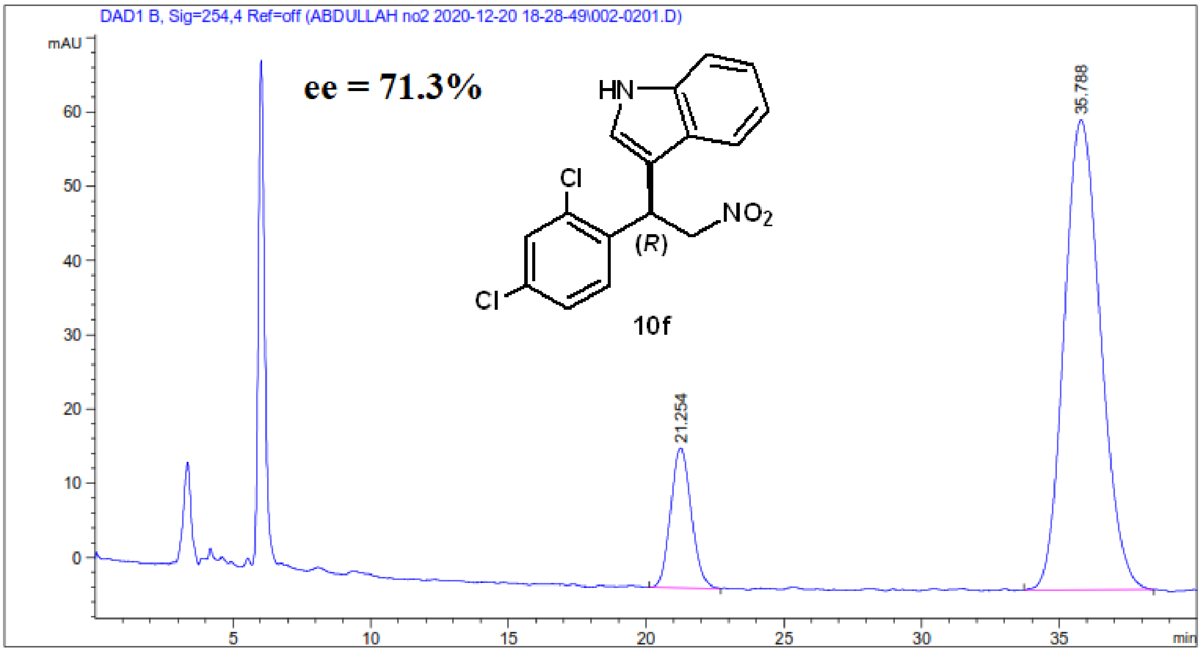

Peak #	RetTime [min]	Type	Width [min]	Area [mAU*s]	Height [mAU]	Area %
1	21.254	BB	0.7251	978.89471	18.81221	14.3735
2	35.788	BB	1.3126	5831.50049	63.31315	85.6265

Chiral HPLC for Friedel Craft product-10g

Racemic-10g

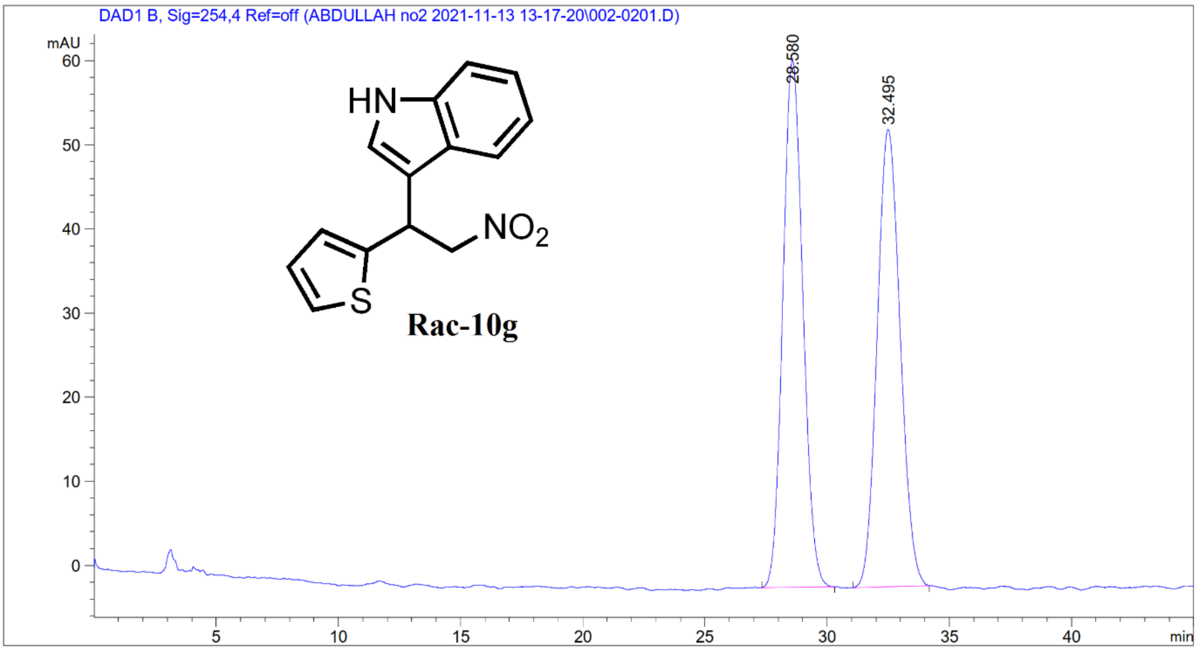

Peak #	RetTime [min]	Type	Width [min]	Area [mAU*s]	Height [mAU]	Area %
1	28.580	BB	0.8479	3564.16528	62.63051	50.0482
2	32.495	BB	0.9794	3557.29810	54.36292	49.9518

Chiral-10g

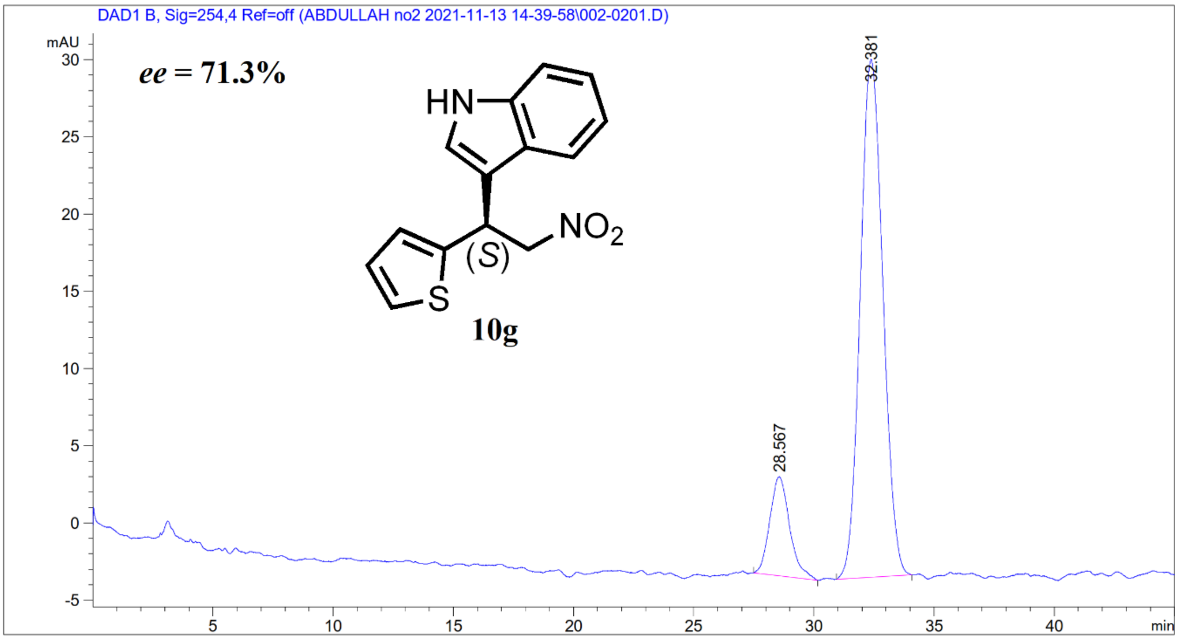

Peak #	RetTime [min]	Type	Width [min]	Area [mAU*s]	Height [mAU]	Area %
1	28.567	BB	0.6783	368.97144	6.42171	14.3654
2	32.381	BB	0.9049	2199.49585	33.54760	85.6346

Chiral HPLC for Friedel Craft product-10h

Racemic-10h

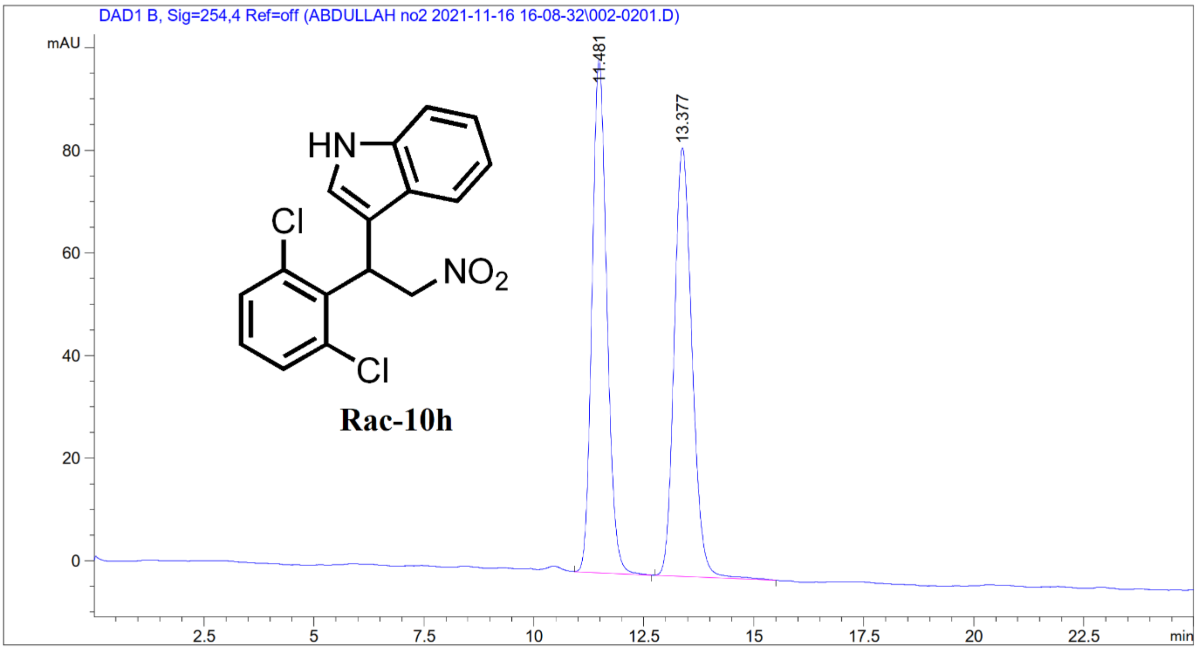

Peak #	RetTime [min]	Type	Width [min]	Area [mAU*s]	Height [mAU]	Area %
1	11.481	BB	0.3629	2335.33472	99.82401	49.8273
2	13.377	BB	0.4347	2351.52124	83.46496	50.1727

Chiral-10h

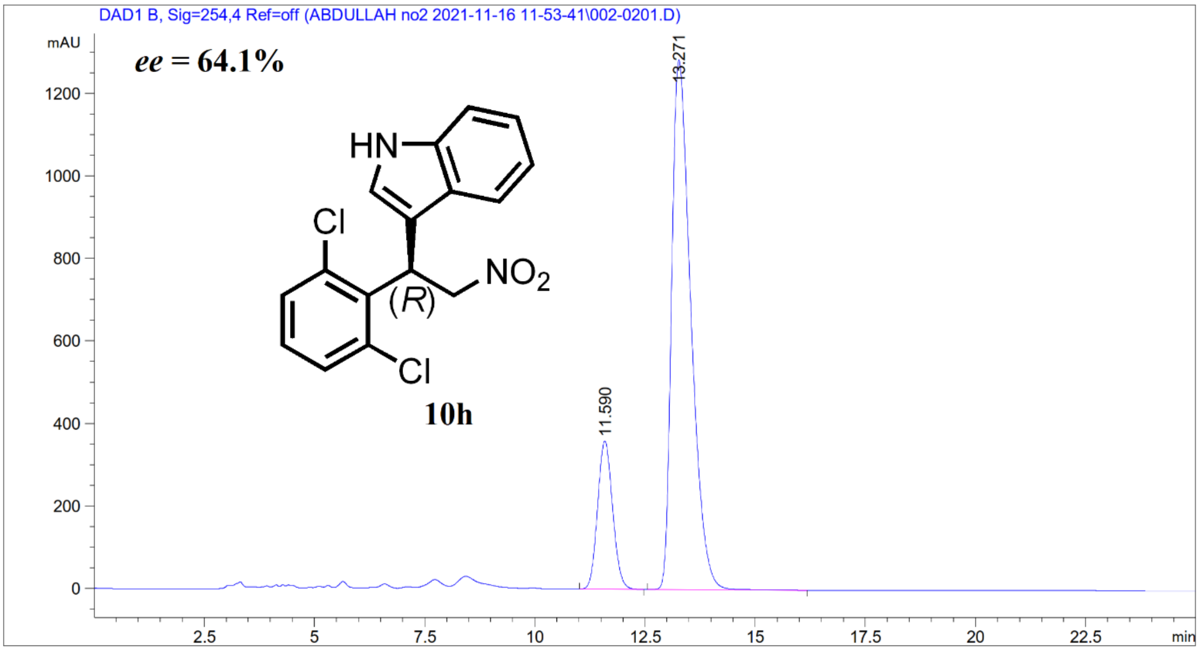

Peak #	RetTime [min]	Type	Width [min]	Area [mAU*s]	Height [mAU]	Area %
1	11.590	BB	0.3680	8494.21484	359.01257	17.9525
2	13.271	BB	0.4629	3.88208e4	1283.44104	82.0475

Chiral HPLC for Friedel Craft product-10i

Racemic-10i

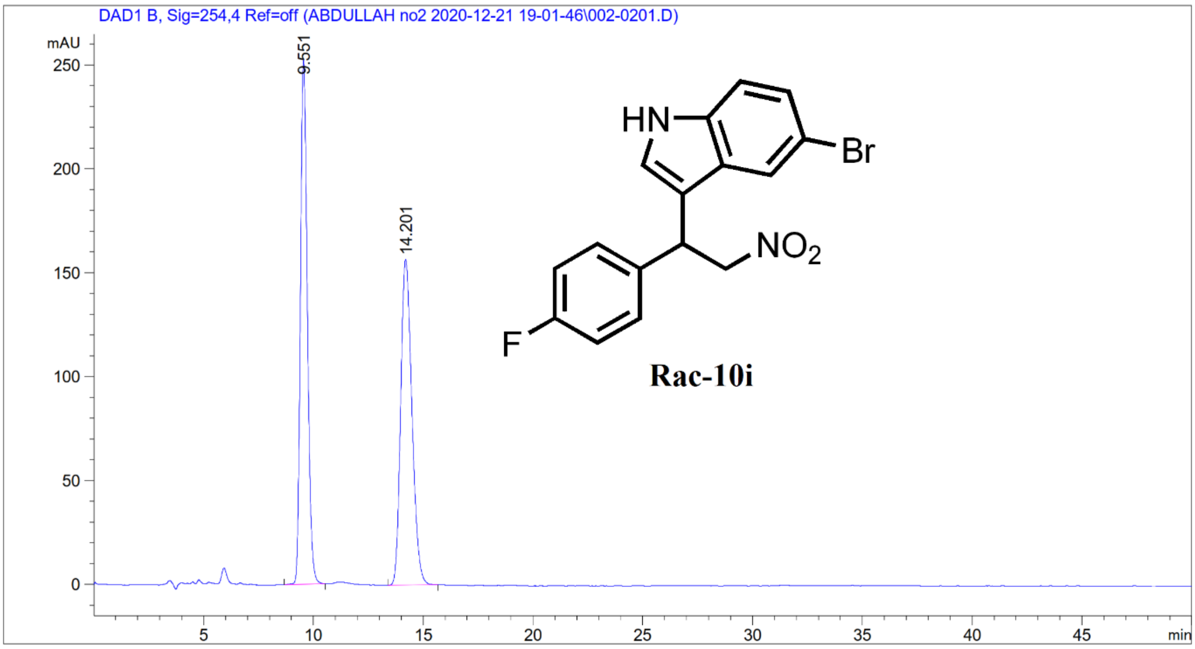

Peak #	RetTime [min]	Type	Width [min]	Area [mAU*s]	Height [mAU]	Area %
1	9.551	BB	0.3398	5533.21045	252.31903	49.8935
2	14.201	BB	0.5513	5556.83350	156.86708	50.1065

Chiral-10i

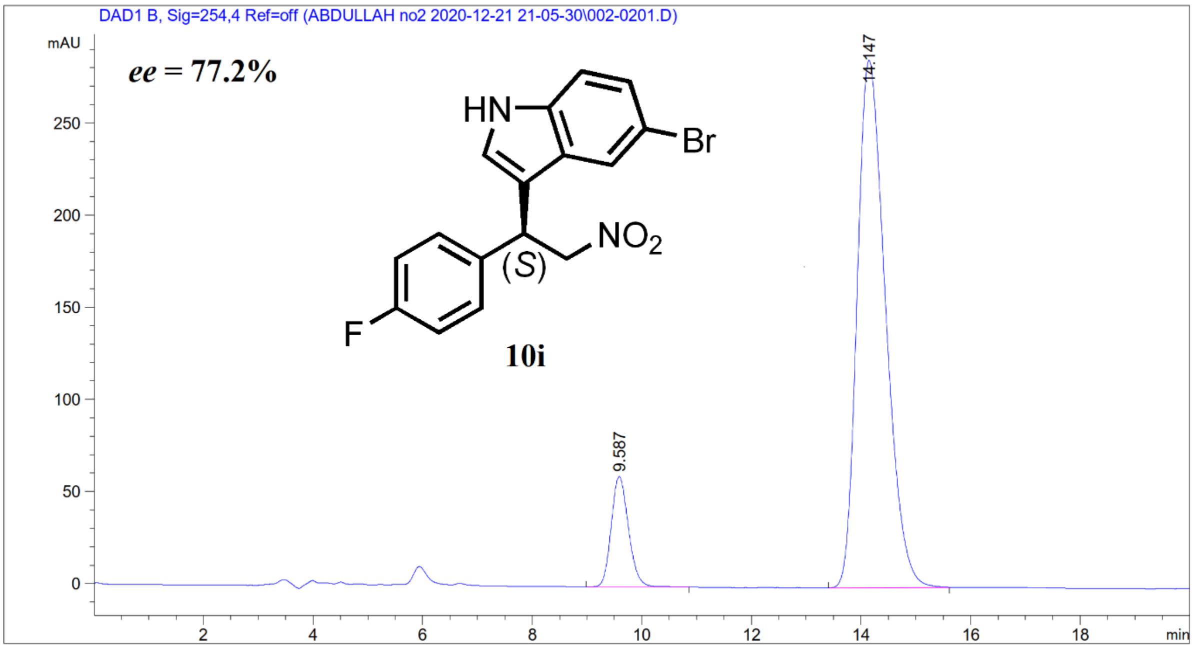

Peak #	RetTime [min]	Type	Width [min]	Area [mAU*s]	Height [mAU]	Area %
1	9.587	BB	0.3408	1319.70349	59.95178	11.4014
2	14.147	BB	0.5559	1.02552e4	286.30734	88.5986

Chiral HPLC for Friedel Craft product-10i

Racemic-10j

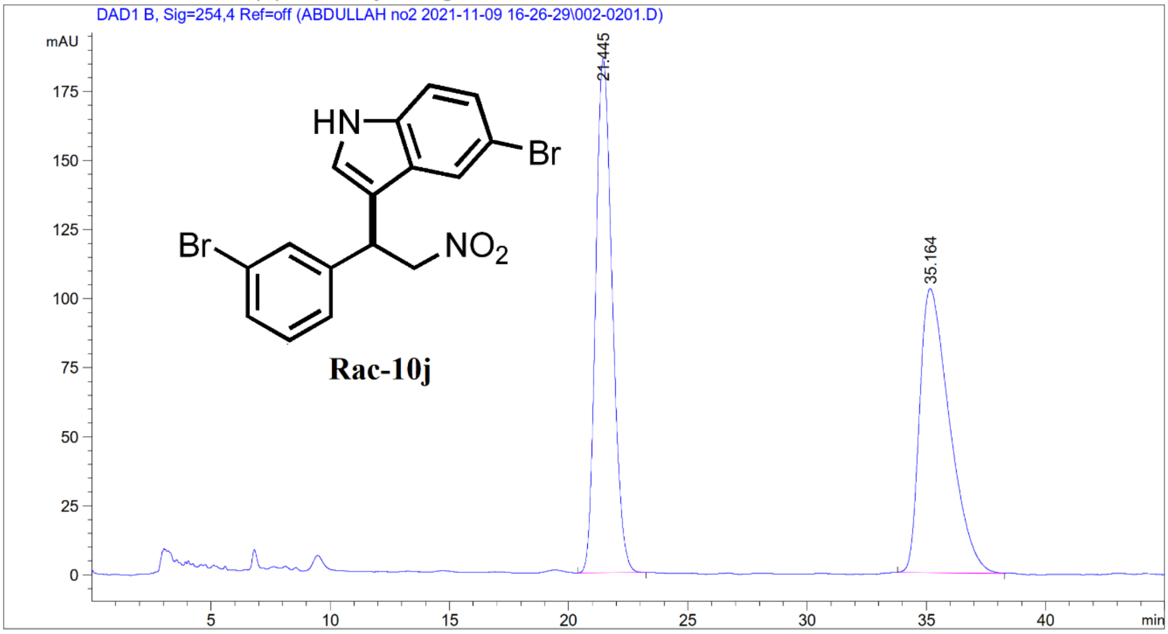

Peak #	RetTime [min]	Type	Width [min]	Area [mAU*s]	Height [mAU]	Area %
1	21.445	BB	0.7490	9021.26563	186.29155	50.1398
2	35.164	BB	1.2537	8970.97363	102.83905	49.8602

Chiral-10j

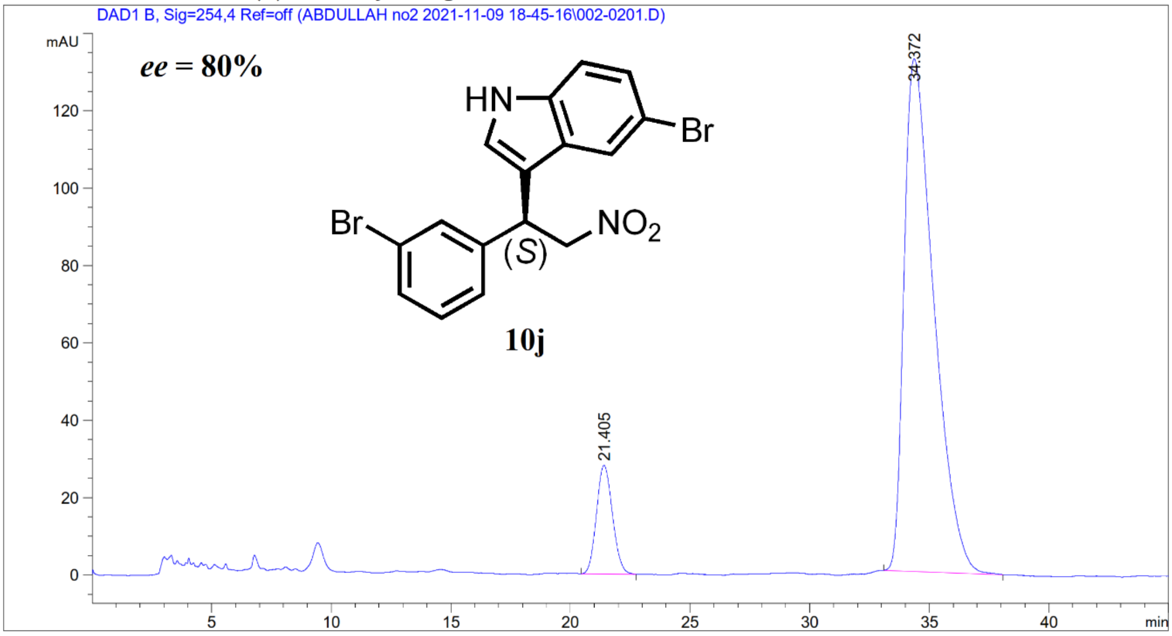

Peak #	RetTime [min]	Type	Width [min]	Area [mAU*s]	Height [mAU]	Area %
1	21.405	BB	0.6926	1310.11938	28.16799	10.2498
2	34.372	BB	1.2576	1.14717e4	132.55782	89.7502

Chiral HPLC for Friedel Craft product-10k

Racemic-10k

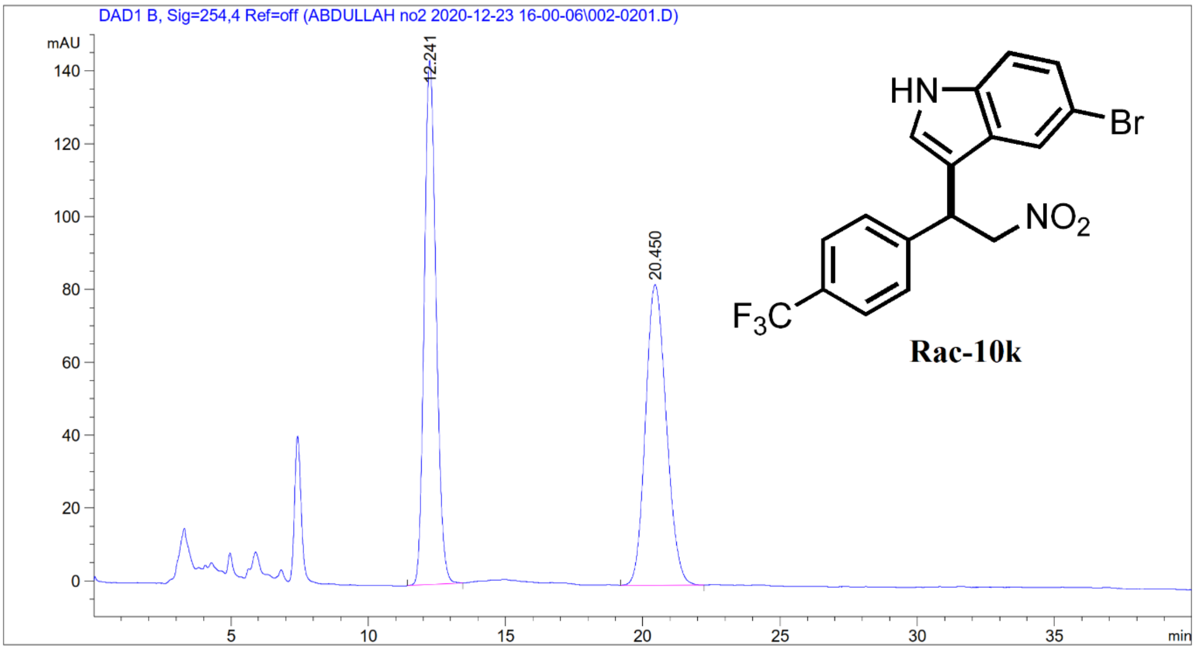

Peak #	RetTime [min]	Type	Width [min]	Area [mAU*s]	Height [mAU]	Area %
1	12.241	BB	0.4668	4303.59326	143.93999	49.6780
2	20.450	BB	0.8105	4359.37695	82.50190	50.3220

Chiral-10k

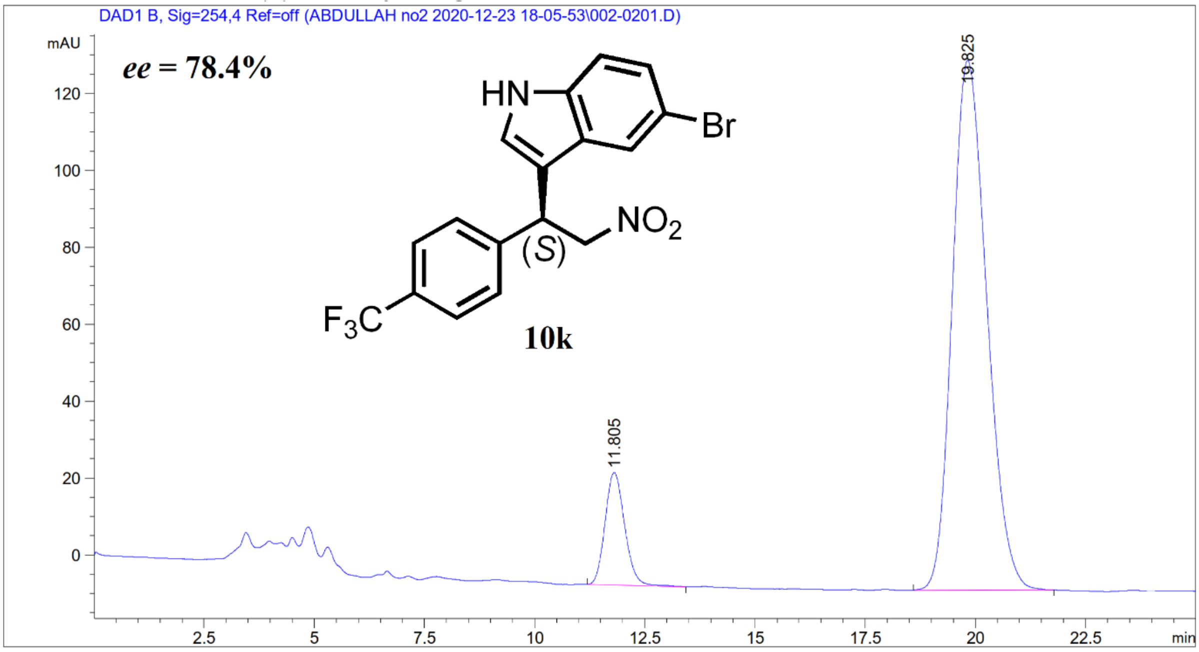

Peak #	RetTime [min]	Type	Width [min]	Area [mAU*s]	Height [mAU]	Area %
1	11.805	BB	0.4774	905.49304	29.23283	10.7856
2	19.825	BB	0.8485	7489.90381	137.71338	89.2144

Chiral HPLC for Friedel Craft product-10l

Racemic-10l

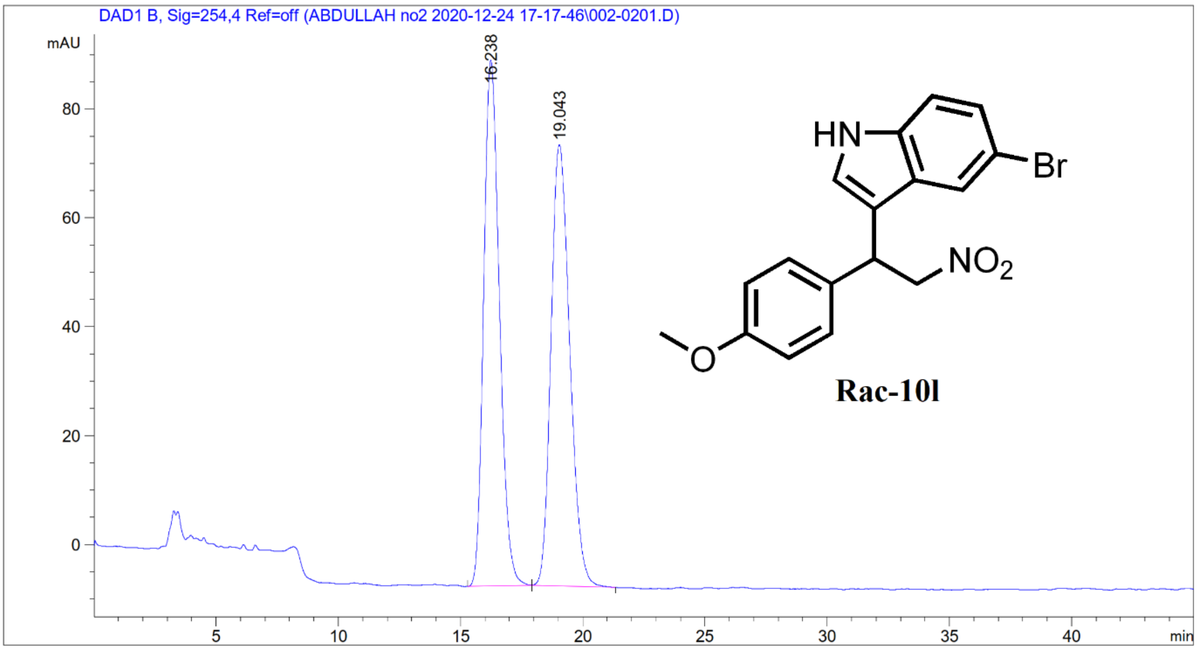

Peak #	RetTime [min]	Type	Width [min]	Area [mAU*s]	Height [mAU]	Area %
1	16.238	BB	0.6922	4322.55420	96.56606	49.9699
2	19.043	BB	0.8251	4327.76709	81.03493	50.0301

Chiral-10l

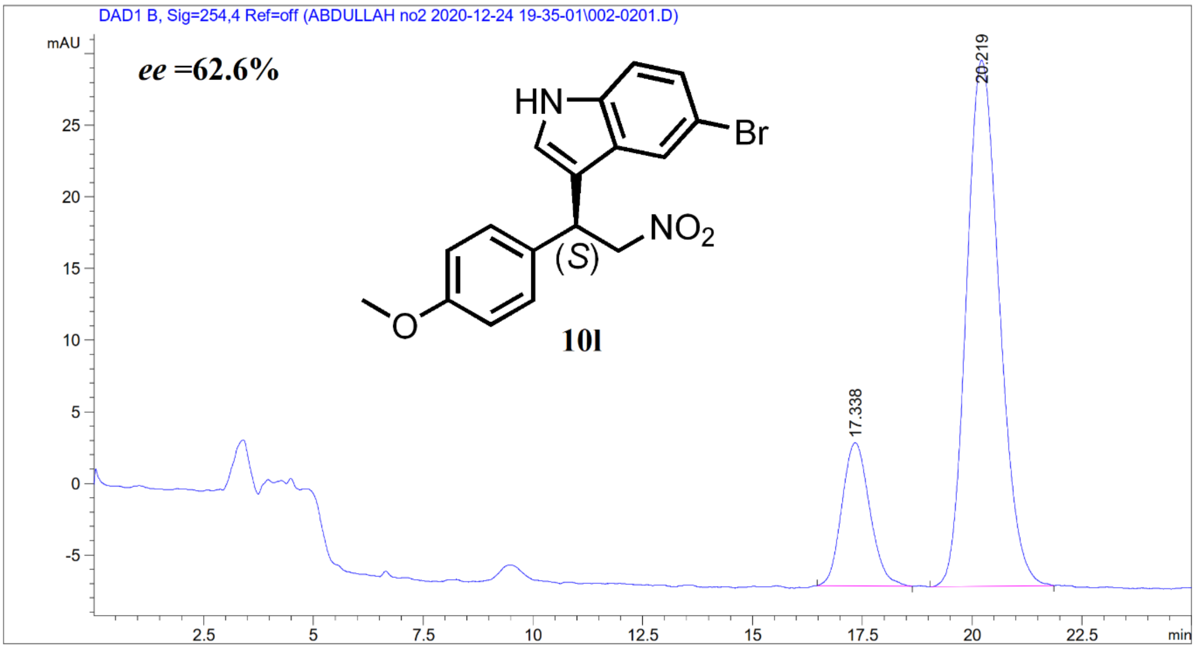

Peak #	RetTime [min]	Type	Width [min]	Area [mAU*s]	Height [mAU]	Area %
1	17.338	BB	0.5546	441.22220	10.00259	18.7052
2	20.219	BB	0.7809	1917.59949	36.74110	81.2948

Chiral HPLC for Friedel Craft product-10m

Racemic-10m

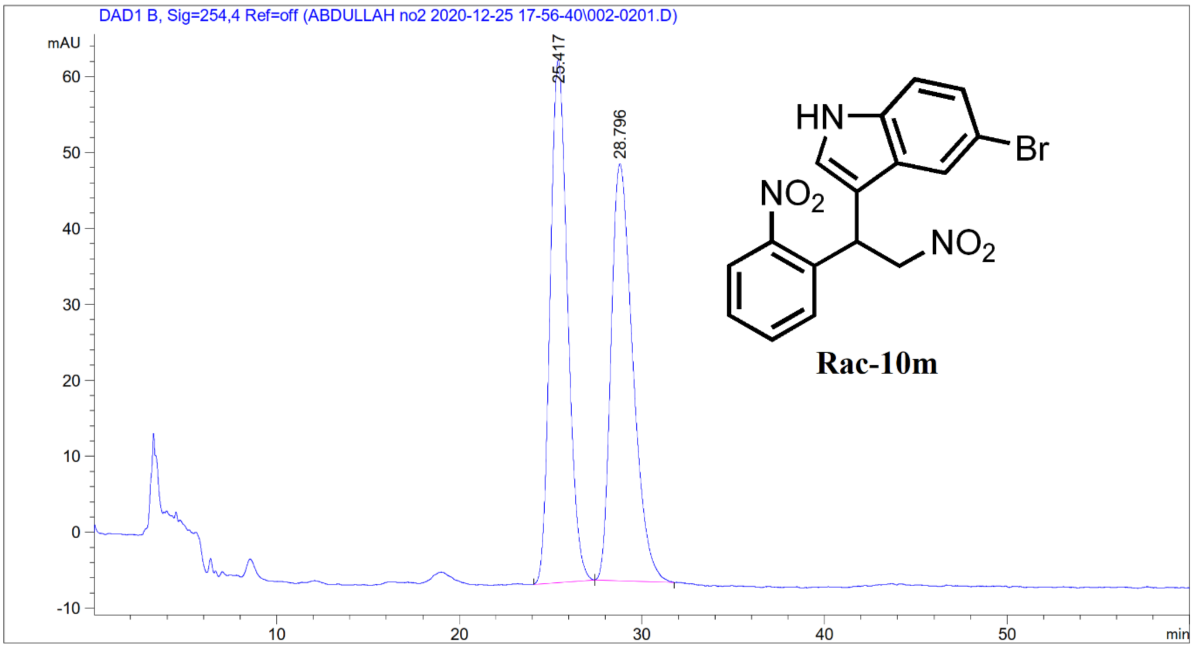

Peak #	RetTime [min]	Type	Width [min]	Area [mAU*s]	Height [mAU]	Area %
1	25.417	BB	1.0100	4564.61914	68.80080	50.0962
2	28.796	BB	1.1500	4547.08154	54.91659	49.9038

Chiral-10m

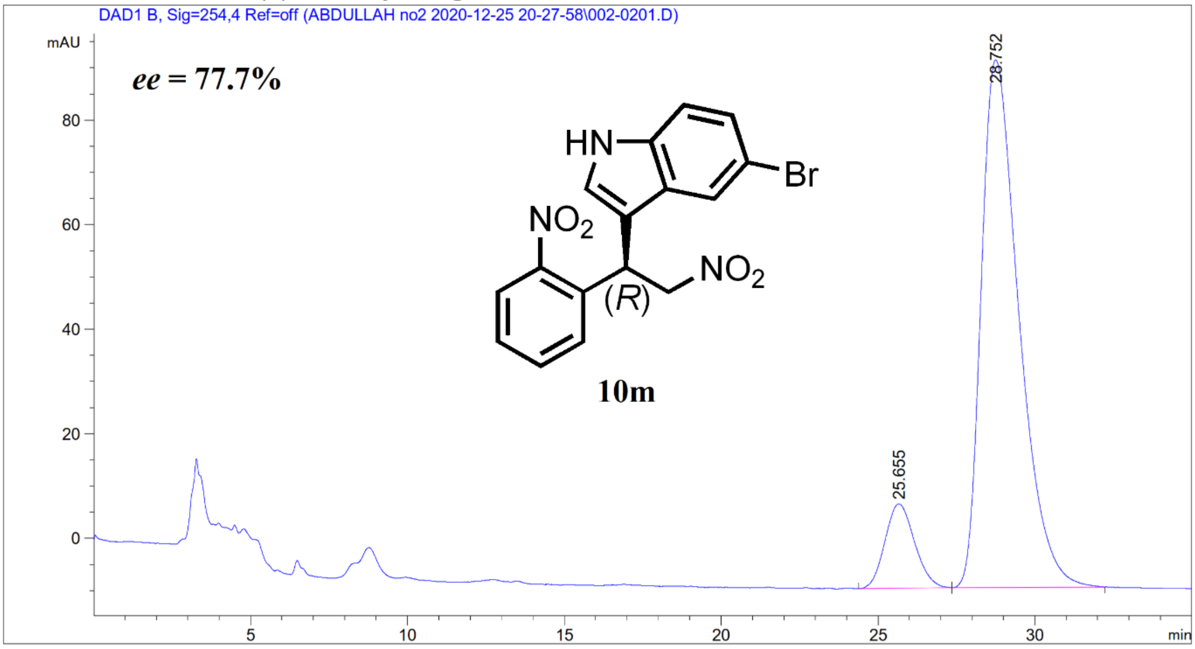

Peak #	RetTime [min]	Type	Width [min]	Area [mAU*s]	Height [mAU]	Area %
1	25.655	BB	0.7815	1056.78052	16.14812	11.1581
2	28.752	BB	1.2202	8414.19238	100.96750	88.8419

Chiral HPLC for Friedel Craft product-10n

Racemic-10n

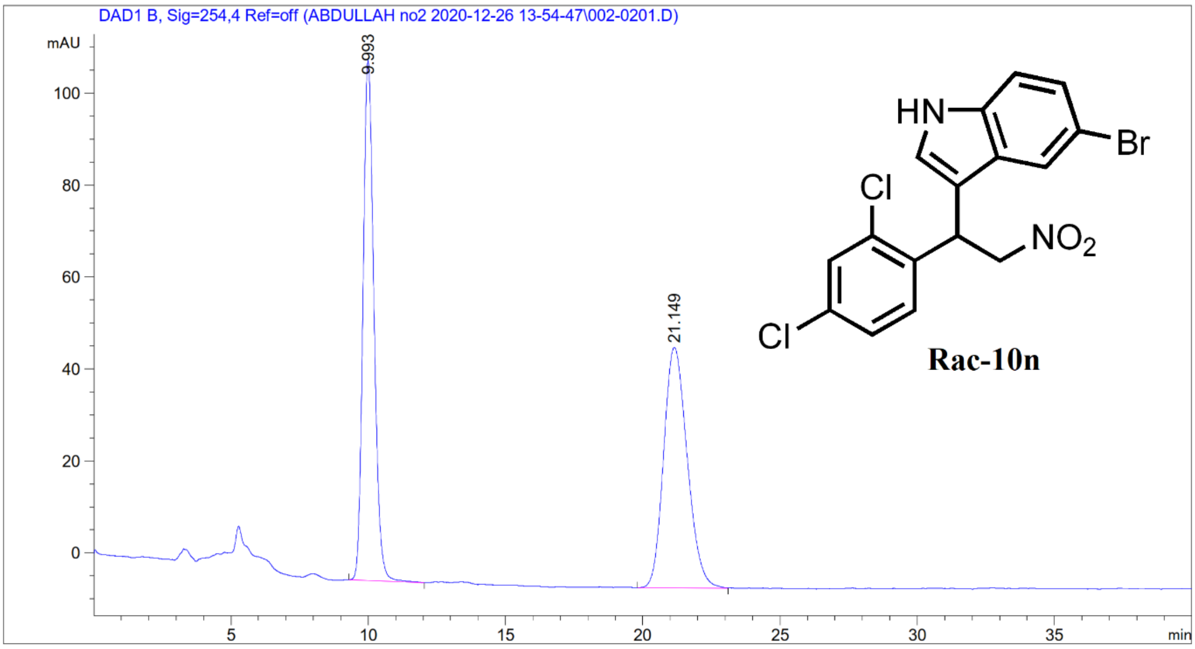

Peak #	RetTime [min]	Type	Width [min]	Area [mAU*s]	Height [mAU]	Area %
1	9.993	BB	0.4262	3124.27808	113.13499	50.0180
2	21.149	BB	0.8819	3122.03369	52.34394	49.9820

Chiral-10n

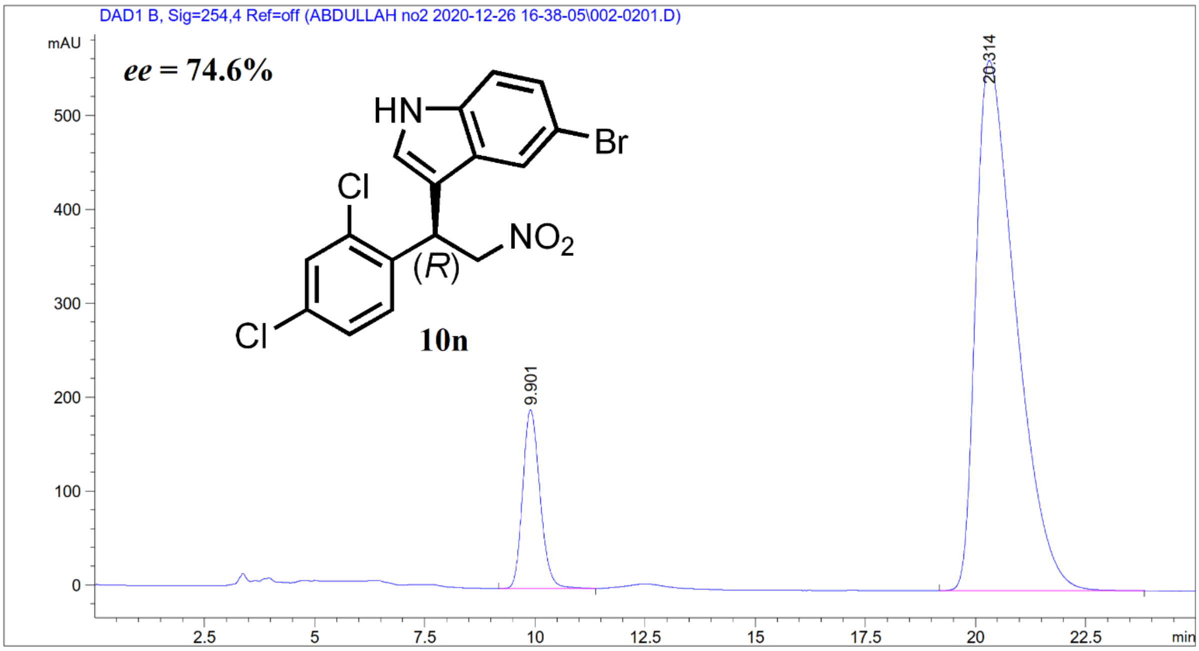

Peak #	RetTime [min]	Type	Width [min]	Area [mAU*s]	Height [mAU]	Area %
1	9.901	BB	0.4207	5200.08838	190.38452	12.7269
2	20.314	BB	0.9552	3.56591e4	564.30743	87.2731

Chiral HPLC for Friedel Craft product-10o

Racemic-10o

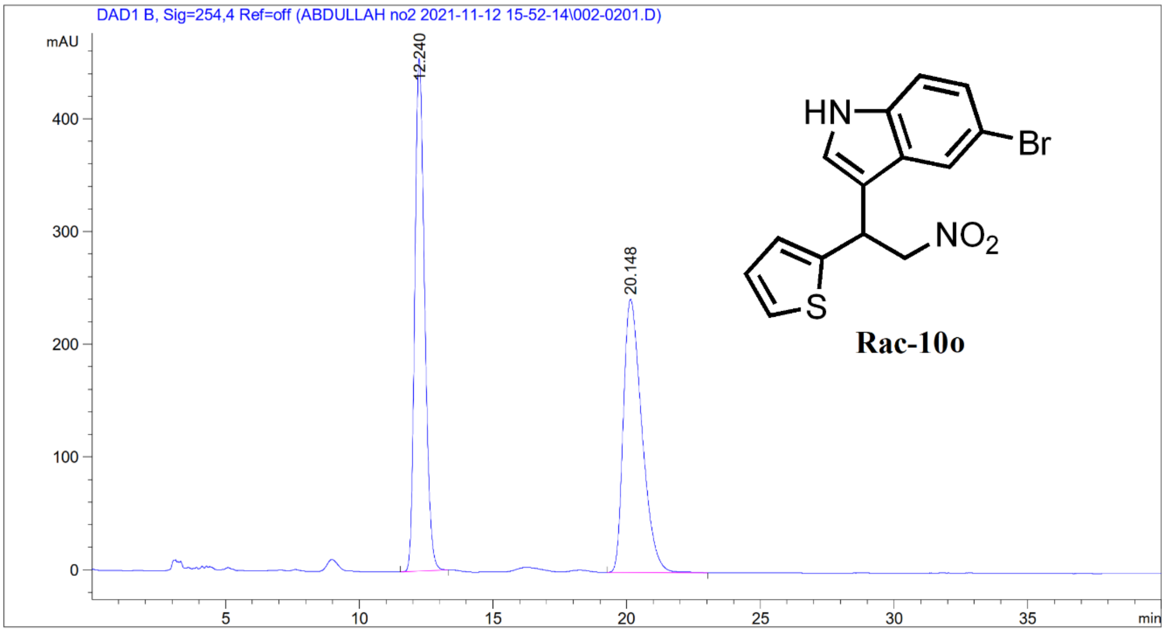

Peak #	RetTime [min]	Type	Width [min]	Area [mAU*s]	Height [mAU]	Area %
1	12.240	BB	0.3965	1.16338e4	454.75760	49.8582
2	20.148	BB	0.7352	1.16999e4	242.36816	50.1418

Chiral-10o

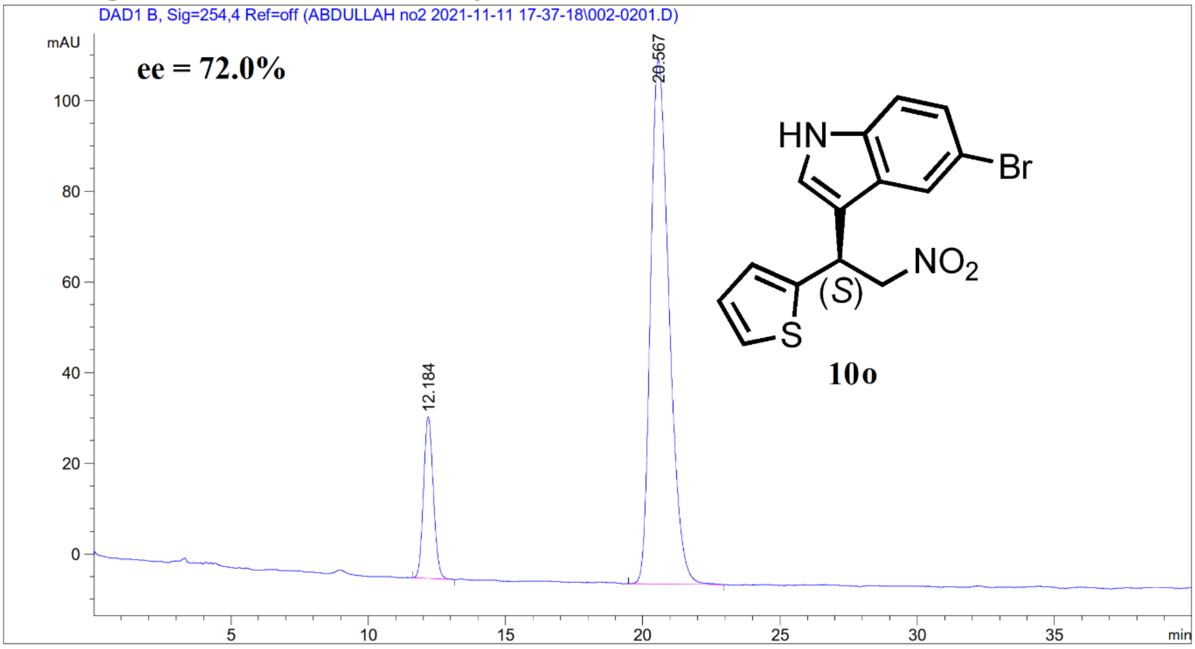

Peak #	RetTime [min]	Type	Width [min]	Area [mAU*s]	Height [mAU]	Area %
1	12.184	BB	0.3866	882.45984	35.67549	13.9947
2	20.567	BB	0.7173	5423.21973	115.61095	86.0053

Chiral HPLC for Friedel Craft product-10p

Racemic-10p

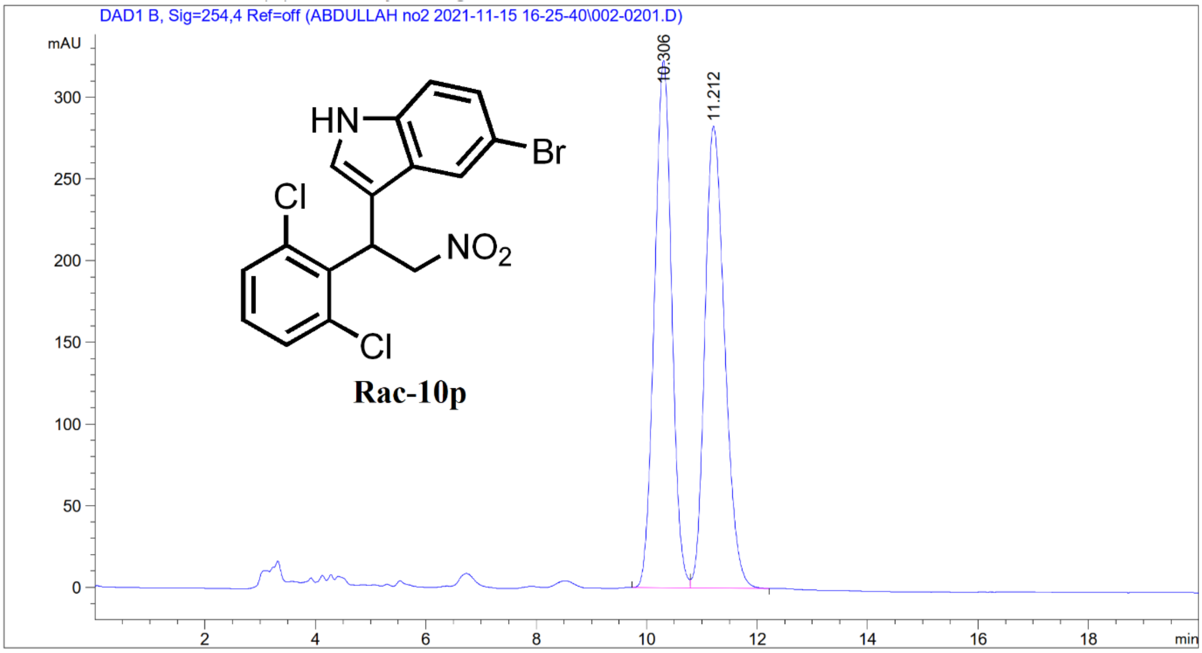

Peak #	RetTime [min]	Type	Width [min]	Area [mAU*s]	Height [mAU]	Area %
1	10.306	BV	0.3372	7007.77344	322.83759	49.8200
2	11.212	VB	0.3870	7058.40771	282.95175	50.1800

Chiral-10p

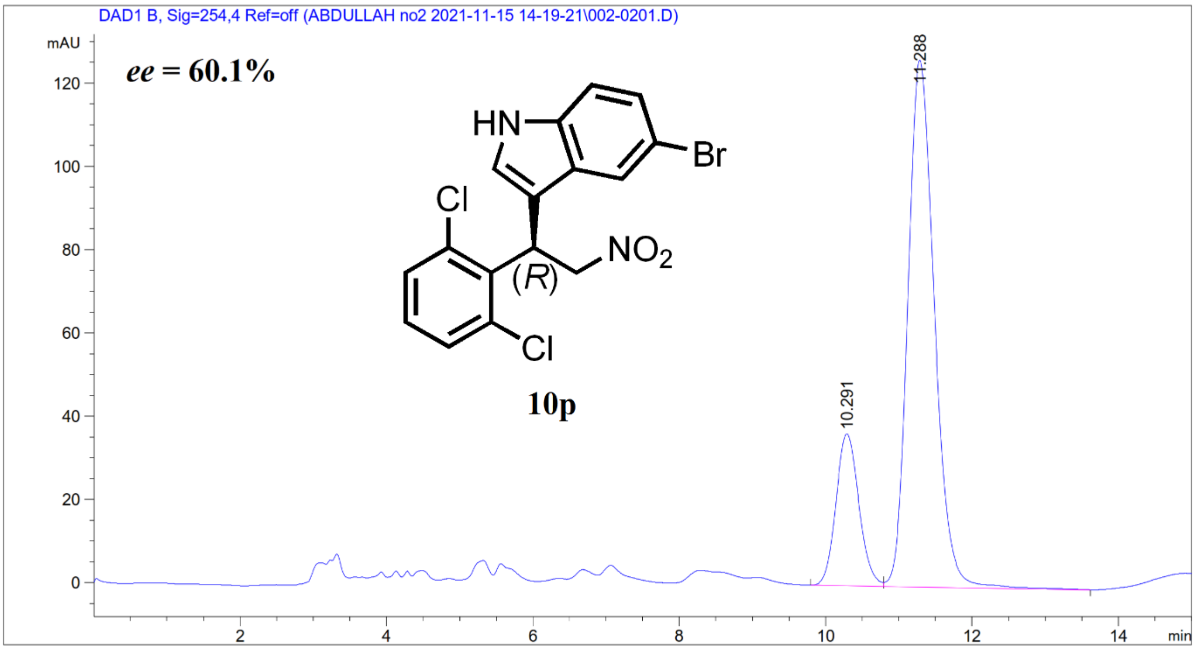

Peak #	RetTime [min]	Type	Width [min]	Area [mAU*s]	Height [mAU]	Area %
1	10.291	BV	0.3381	794.60425	36.47799	19.9554
2	11.288	VB	0.3880	3187.30347	126.49233	80.0446

Chiral HPLC for Friedel Craft product-10q

Racemic-10q

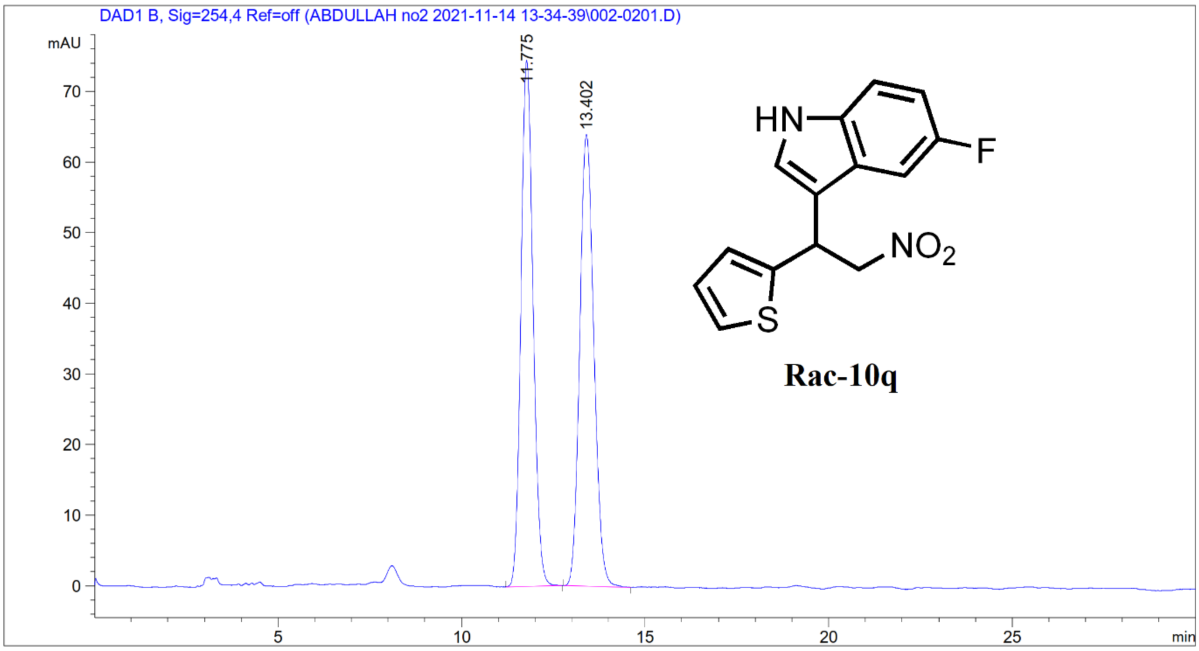

Peak #	RetTime [min]	Type	Width [min]	Area [mAU*s]	Height [mAU]	Area %
1	11.775	BB	0.3599	1710.29626	74.47244	49.9485
2	13.402	BB	0.4165	1713.82349	63.97464	50.0515

Chiral-10q

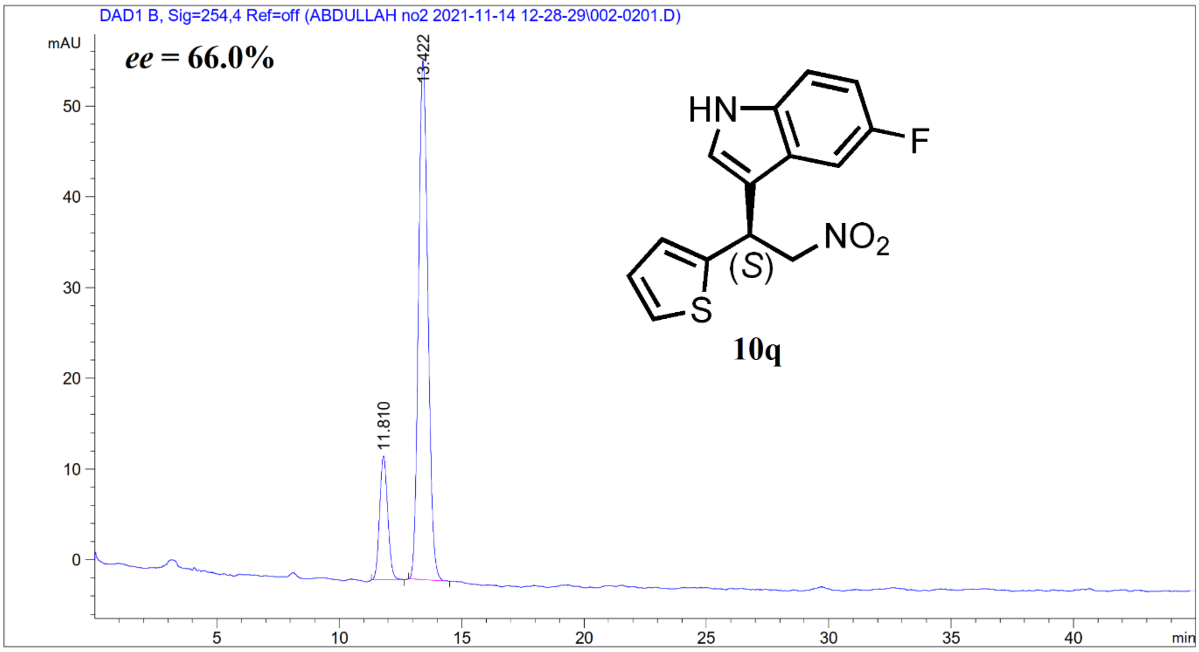

Peak #	RetTime [min]	Type	Width [min]	Area [mAU*s]	Height [mAU]	Area %
1	11.810	BB	0.3515	313.25510	13.66288	17.0040
2	13.422	BB	0.4158	1528.98450	57.20486	82.9960

Chiral HPLC for Friedel Craft product-10r

Racemic-10r

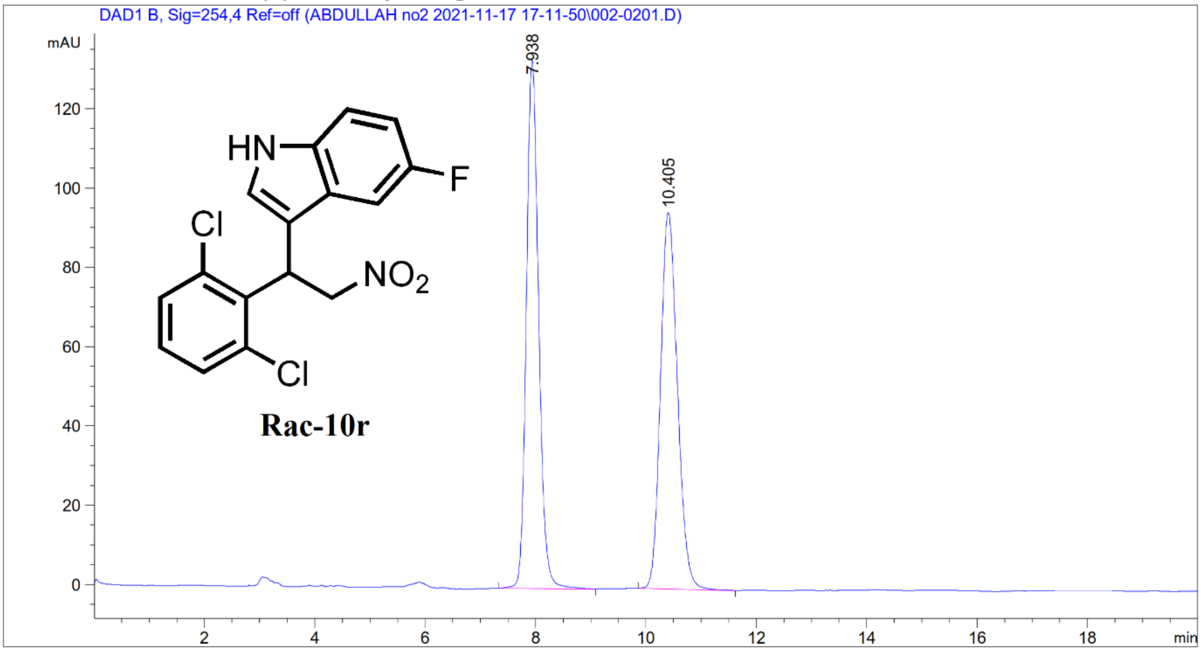

Peak #	RetTime [min]	Type	Width [min]	Area [mAU*s]	Height [mAU]	Area %
1	7.938	BB	0.2377	2052.45142	133.27838	50.3214
2	10.405	BB	0.3330	2026.23706	94.94862	49.6786

Chiral-10r

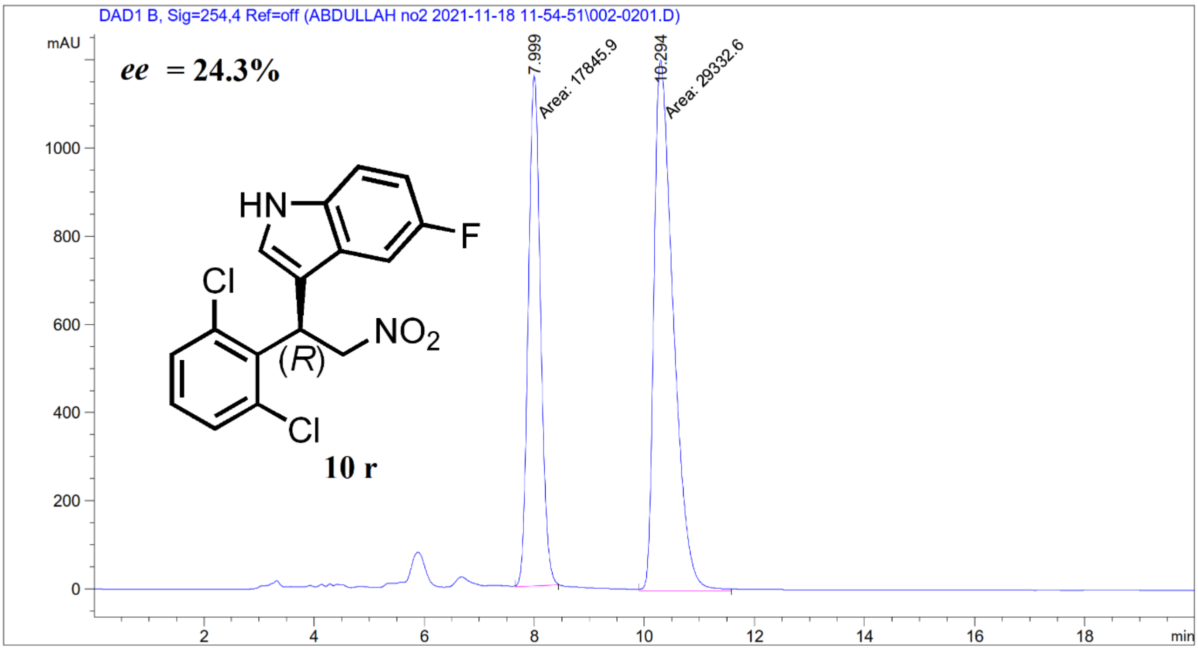

Peak #	RetTime [min]	Type	Width [min]	Area [mAU*s]	Height [mAU]	Area %
1	7.999	MM	0.2572	1.78459e4	1156.26660	37.8263
2	10.294	MM	0.4064	2.93326e4	1202.98206	62.1737

Chiral HPLC for Friedel Craft product-10s

Racemic-10s

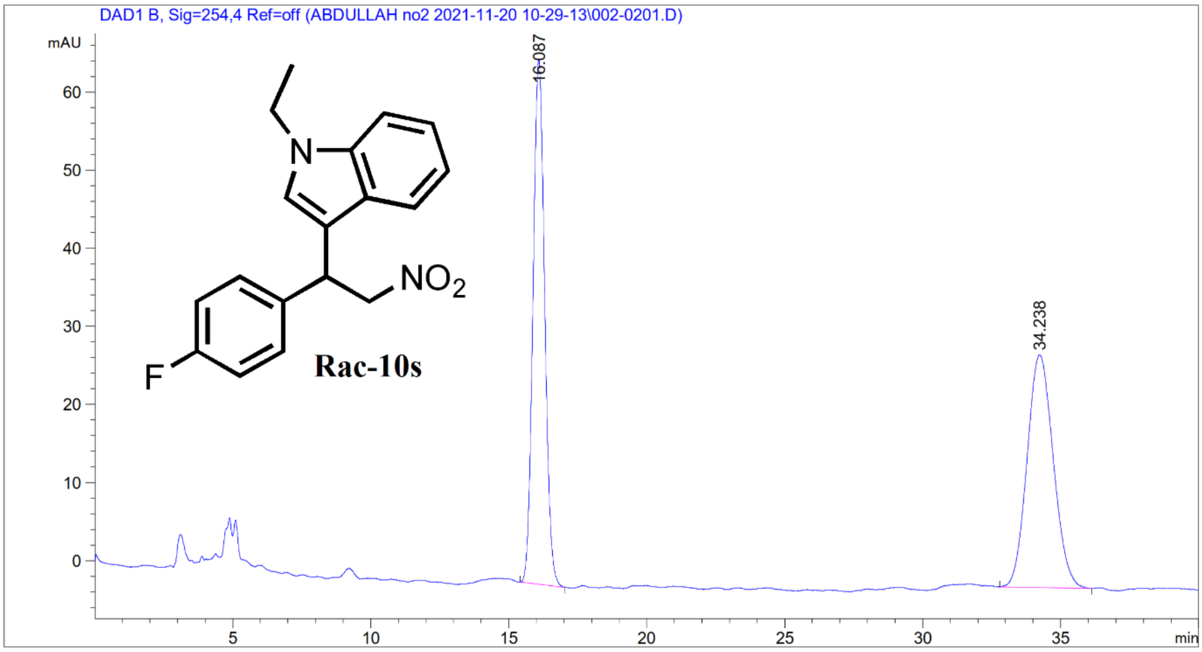

Peak #	RetTime [min]	Type	Width [min]	Area [mAU*s]	Height [mAU]	Area %
1	16.087	BB	0.4542	1969.31995	67.14613	50.0263
2	34.238	BB	0.9215	1967.24878	29.82510	49.9737

Chiral-10s

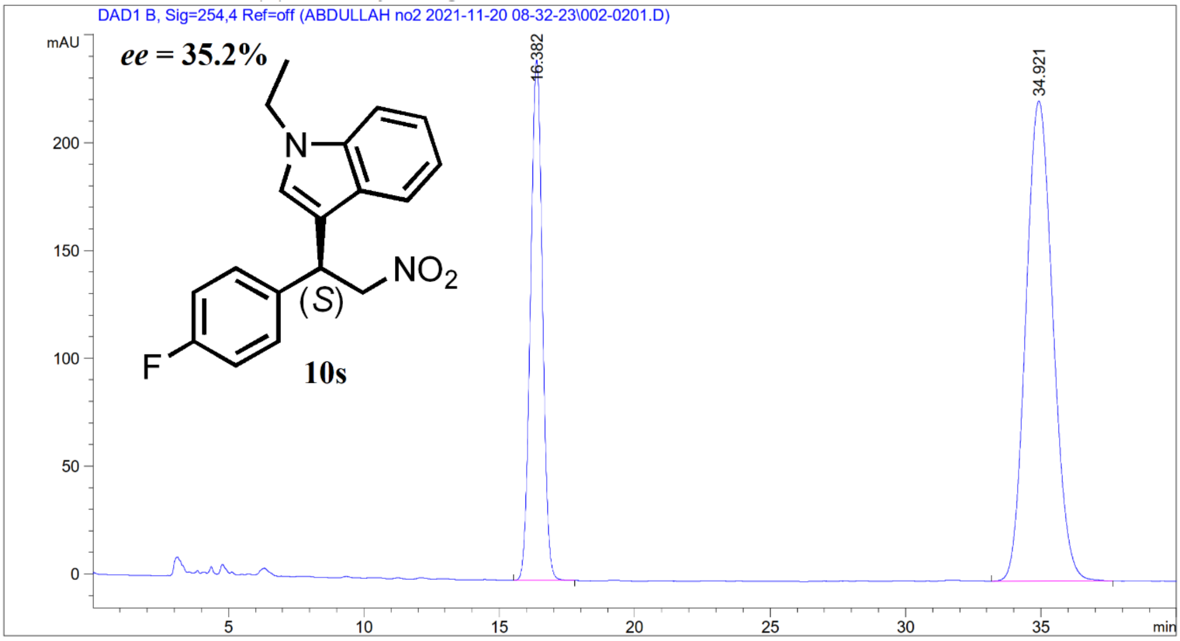

Peak #	RetTime [min]	Type	Width [min]	Area [mAU*s]	Height [mAU]	Area %
1	16.382	BB	0.4797	7407.38721	241.62900	32.3954
2	34.921	BB	1.0800	1.54582e4	222.64345	67.6046

Chiral HPLC for Friedel Craft product-10t

Racemic-10t

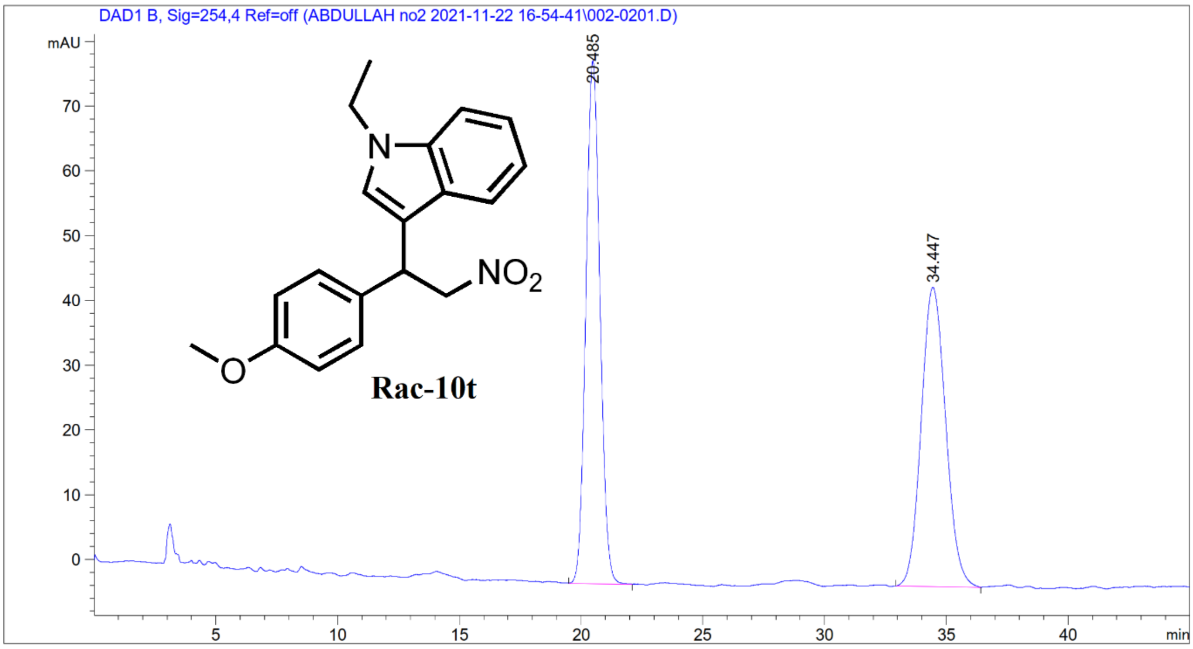

Peak #	RetTime [min]	Type	Width [min]	Area [mAU*s]	Height [mAU]	Area %
1	20.485	BB	0.6213	3237.59497	80.82733	50.0897
2	34.447	BB	1.0082	3225.99780	46.22754	49.9103

Chiral-10t

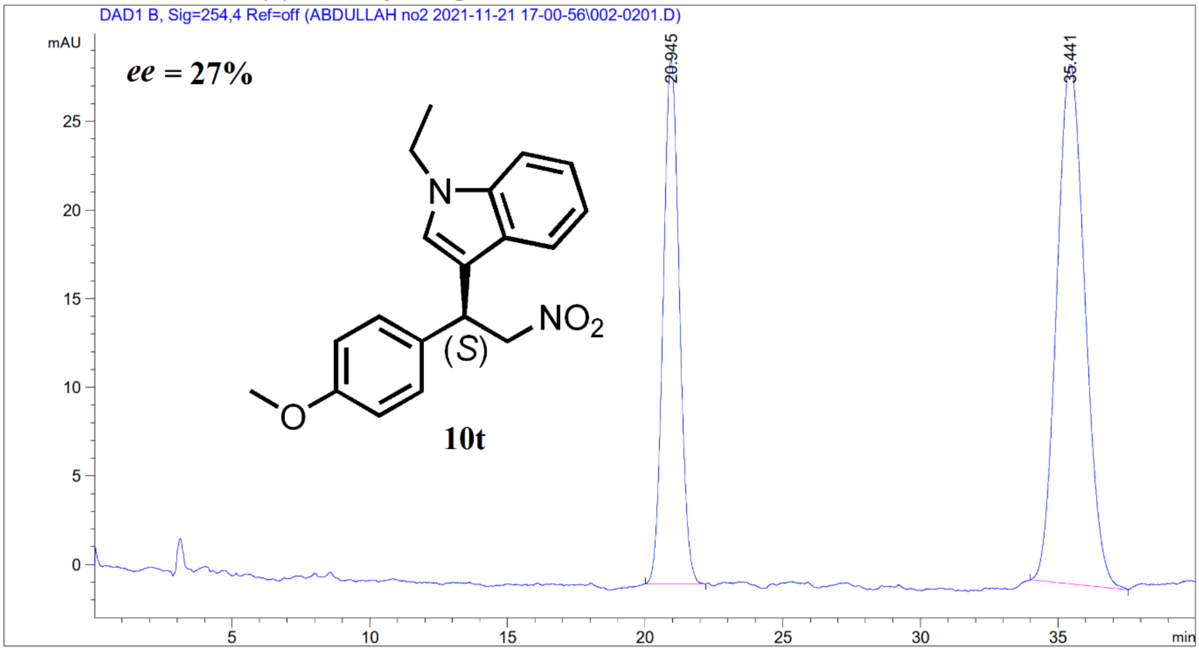

Peak #	RetTime [min]	Type	Width [min]	Area [mAU*s]	Height [mAU]	Area %
1	20.945	BB	0.6260	1205.34802	29.54569	36.6300
2	35.441	BB	0.9818	2085.25098	29.31736	63.3700

4. X-Ray structure determinations for SI

The crystal of **9i** and **9j** were immersed in cryo-oil, mounted in a loop, and measured at a temperature of 150 K. The X-ray diffraction data was collected on a Rigaku Oxford Diffraction Supernova diffractometer using Mo Kα radiation. The *CrysAlisPro* [2] software package was used for cell refinement and data reduction. A gaussian (**9i**) or analytical (**9j**) absorption correction (*CrysAlisPro [2]*) was applied to the intensities before structure solution. The structures were solved by intrinsic phasing (*SHELXT* [3]) method. Structural refinement was carried out using *SHELXL* [4] software with *SHELXLE* [5] graphical user interface. The NH hydrogen atom in **9i** was located from the difference Fourier map and refined isotropically. All other hydrogen atoms were positioned geometrically and constrained to ride on their parent atoms, with C-H = 0.95-0.98 Å and U_{iso} = 1.2-1.5·U_{eq} (parent atom).

Crystal data for compounds 9i and 9j

Compounds, **9i** and **9j** are solved in centrosymmetric space groups P21/n and P21/c respectively. Both molecules are nearly planar. Replacement of NH hydrogen with a methyl group does not have significant impact on the structural details of the rest of the molecules. Compound, **9i** is connected to the neighboring molecule *via* N(1)H(1)···O(1) hydrogen bond (N(1)-H1: 0.86(3) Å, H(1)···O(1)$^{\#1}$: 2.25 Å, N(1)H(1)···O(1)$^{\#1}$: 149°). Due to the lack of NH hydrogen atom, **9j** does not form conventional H-bonds but the methyl hydrogens and C(1)-H(1) as well as C(3)-H(3) form weak hydrogen bonds with O2 and O1 atoms of the neighboring molecules (C(11)-H(11A) 0.98 Å, H(11A)···O(2)$^{\#2}$: 2.521 Å, C(11)-H(11A)···O(2)$^{\#2}$: 153.46°, C(11)-H(11C) 0.98 Å, H(11C)···O(1)$^{\#3}$: 2.427 Å, C(11)-H(11C)···O(1)$^{\#3}$: 158.86°, #1: x-1/2, -y+1/2, z+1/2, #2: -x+1, -y, -z+1, #3: x-1, -y+1/2, z-1/2, #4: -x+1, -y, -z+, #5: x-1, y, z-1).

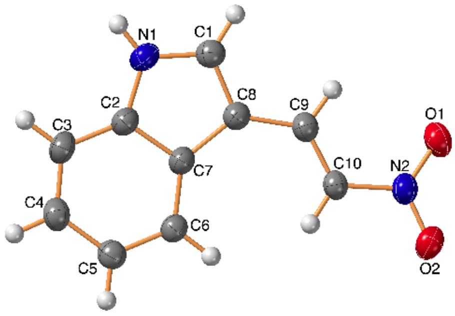

9i

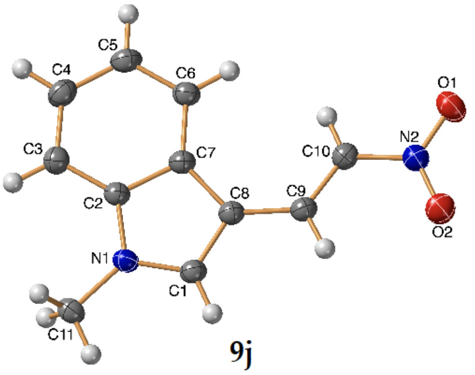

9j

Figure 3: ORTEP of compounds **9i** (left) and **9j** (right).

Table 4. Crystal Data of (**9i** and **9j**)

	**9i**	**9j**
CCDC	2115146	2115377
empirical formula	$C_{10}H_8N_2O_2$	$C_{11}H_{10}N_2O_2$
fw	188.18	202.21
temp (K)	150(2)	150(2)
λ(Å)	0.71073	0.71073
cryst syst	Monoclinic	Monoclinic
space group	$P2_1/n$	$P2_1/c$
a (Å)	8.2983(8)	9.6703(3)
b (Å)	10.6235(10)	13.1040(4)
c (Å)	10.1697(12)	7.6970(3)
α (deg)	90	90
β (deg)	106.030(11)	102.012(4)
γ (deg)	90	90
V (Å^3)	861.67(16)	954.00(6)

Z	4	4
ρ_{calc} (Mg/m^3)	1.451	1.408
μ(Mo Kα) (mm^{-1})	0.104	0.099
No. reflns.	17532	8331
Unique reflns.	2132	2366
Completeness to θ=25.242°	99.9%	99.7%
GOOF (F^2)	1.103	1.028
R_{int}	0.0910	0.0361
R_1[a] ($I \geq 2\sigma$)	0.0556	0.0486
wR_2[b] ($I \geq 2\sigma$)	0.1497	0.1294

[a] $R_1 = \Sigma||F_o| - |F_c||/\Sigma|F_o|$. [b] $wR_2 = [\Sigma[w(F_o^2 - F_c^2)^2]/ \Sigma[w(F_o^2)^2]]^{1/2}$.

References

1. Liang, L.; Liu, Q.; Zhang, J.; Wang, F.; Yuan, Y. Efficient iron-catalyzed Michael addition of indole to nitroolefins under solvent-free conditions. *Res. Chem. Intermed.* **2013**, *39*, 1957-1962.
2. Rikagu Oxford Diffraction, CrysAlisPro, Agilent Technologies inc., 2018, Yarnton, Oxfordshire, England.
3. Sheldrick, G.M. SHELXT–Integrated space-group and crystal-structure determination. *Acta Crystallogr. A* **2015**, *71*, 3-8.
4. Sheldrick, G.M. Crystal structure refinement with SHELXL. *Acta Crystallogr. C Struct. Chem.* **2015**, *71*, 3-8.
5. Hübschle, C.B.; Sheldrick, G.M.; Dittrich, B. ShelXle: a Qt graphical user interface for SHELXL. *J. Appl. Crystallogr.* **2011**, *44*, 1281-1284.
